# Predicting 30-day readmission following total knee arthroplasty using machine learning and clinical expertise applied to clinical administrative and research registry data in an Australian cohort

**DOI:** 10.1186/s42836-023-00186-3

**Published:** 2023-06-01

**Authors:** Daniel J. Gould, James A. Bailey, Tim Spelman, Samantha Bunzli, Michelle M. Dowsey, Peter F. M. Choong

**Affiliations:** 1 Department of Surgery, St Vincent’s Hospital Melbourne, University of Melbourne, Level 2 Clinical Sciences Building, 29 Regent Street, Fitzroy, VIC 3065 Australia; 2 School of Computing and Information Systems, University of Melbourne, Doug McDonell Building, Parkville, VIC 3052 Australia; 3 School of Health Sciences and Social Work, Griffith University, Nathan Campus, Nathan, QLD 4111 Australia; 4 Department of Orthopaedics, St. Vincent’s Hospital Melbourne, Level 3/35 Victoria Parade, Fitzroy, VIC 3065 Australia

**Keywords:** Readmission, Total knee arthroplasty, Machine learning, Registry data

## Abstract

**Background:**

Thirty-day readmission is an increasingly important problem for total knee arthroplasty (TKA) patients. The aim of this study was to develop a risk prediction model using machine learning and clinical insight for 30-day readmission in primary TKA patients.

**Method:**

Data used to train and internally validate a multivariable predictive model were obtained from a single tertiary referral centre for TKA located in Victoria, Australia. Hospital administrative data and clinical registry data were utilised, and predictors were selected through systematic review and subsequent consultation with clinicians caring for TKA patients. Logistic regression and random forest models were compared to one another. Calibration was evaluated by visual inspection of calibration curves and calculation of the integrated calibration index (ICI). Discriminative performance was evaluated using the area under the receiver operating characteristic curve (AUC-ROC).

**Results:**

The models developed in this study demonstrated adequate calibration for use in the clinical setting, despite having poor discriminative performance. The best-calibrated readmission prediction model was a logistic regression model trained on administrative data using risk factors identified from systematic review and meta-analysis, which are available at the initial consultation (ICI = 0.012, AUC-ROC = 0.589). Models developed to predict complications associated with readmission also had reasonable calibration (ICI = 0.012, AUC-ROC = 0.658).

**Conclusion:**

Discriminative performance of the prediction models was poor, although machine learning provided a slight improvement. The models were reasonably well calibrated, meaning they provide accurate patient-specific probabilities of these outcomes. This information can be used in shared clinical decision-making for discharge planning and post-discharge follow up.

**Supplementary Information:**

The online version contains supplementary material available at 10.1186/s42836-023-00186-3.

## Introduction

Unplanned hospital readmission following total knee arthroplasty (TKA) disrupts the patient’s recovery and incurs high costs to the healthcare system [1–3]. Readmission can be predicted according to the patient’s characteristics and a broad range of risk factors [4]. Although reasonable accuracy can be achieved in certain clinical settings [5, 6], predicting outcomes, especially readmission, is often difficult in TKA patients [4, 5]. This is due to many factors, including a lack of data of sufficient granularity to discriminate between TKA patients who experience deleterious outcomes and those who have an uncomplicated postoperative course [4, 7].

Machine learning algorithms offer an avenue for potential predictive performance gain given their ability to capture complex patterns in the data [4, 7]. This technique does not rely on pre-specified relationships between predictors and outcomes, instead utilising computational and statistical principles to derive patterns without direct human input. Clinical insight into predictor selection can be used in conjunction with machine learning to increase the clinical relevance and interpretability of the model [8].

The aim of this study was to develop a clinically applicable multivariable predictive model for 30-day readmission following TKA, compliant with best practice guidelines [9], to be used in shared decision-making between patient and surgeon.

## Materials and methods

### Patient selection

Inclusion criteria: all primary TKA patients identified in the administrative database at the study hospital for whom data were available in the St Vincent’s Melbourne Arthroplasty Outcomes (SMART) registry, including simultaneous bilateral procedures, TKA for inflammatory arthropathies, and TKA for traumatic aetiologies. Administrative data are available for use in the live clinical environment, whereas the SMART registry contains additional clinically relevant information not available in the live clinical setting which might improve predictive performance. The SMART registry is a prospective registry comprising longitudinal data for TKA and total hip arthroplasty patients at the study hospital, with 100% capture of elective procedures. It has been described in detail previously [10]. Unplanned 30-day readmission was defined as readmission to the hospital for a complication, or monitoring for a suspected complication, within 30 days following discharge from the orthopaedic unit after TKA surgery, for any cause. This included admission to non-orthopaedic units for any reason that was not part of the routine postoperative course or was not planned for any other reason related to the patient’s comorbidities. Exclusion criteria: revision, unicondylar, and patellofemoral arthroplasty, planned readmissions including admissions to the hospital for other procedures such as chemotherapy or other planned surgical procedures. Admissions to the rehabilitation unit or “hospital in the home” service was also excluded.

### Data processing

Data were randomly split into training (75%) and testing (25%) sets, with testing set data kept separate from training set data in every step of the model development and evaluation processes. A seed was set to ensure reproducibility in random number generation for reproducibility of results and to ensure the test set was not used in any stage of the model training process. A primary TKA procedure was considered a case, with both primary TKA surgeries for each individual patient with a bilateral TKA grouped into either the training set or testing set. All models were trained using fivefold cross-validation with 10 repeats [11] to obtain a more stable estimate of a training set performance before evaluating models on the testing set.

The value of machine learning was also explored, in terms of its ability to be used in conjunction with evidence from the literature and clinical insight on readmission risk factors to enhance predictive performance and clinical applicability, and to determine how well these population-level risk factors translated into a risk prediction model for individualised patient prognostication based on specific risk profiles. Knowledge of risk factors provides population-level information regarding patient characteristics that are associated with readmission, whereas predictive models provide individualised patient-level probability estimates for the patient’s risk [12]. The value of information available at discharge from the hospital following TKA surgery was also evaluated in terms of its ability to enhance predictive performance. The model was developed with the future intention of being implemented in a hospital’s existing information technology infrastructure, facilitating automatic information retrieval from the patient’s medical record. However, the added value of information available in a research registry was also explored.

There were four considerations for models developed in this study:

1. Temporal availability of predictors: initial consultation with the orthopaedic surgeon, specifically when TKA surgery is offered to the patient, or immediately prior to discharge. The rationale for this was that a model using data available at the initial consultation would allow the maximum amount of time to implement risk mitigation strategies and discharge planning.
2. Model architecture: logistic regression or random forest. Logistic regression is commonly used for the prediction of binary outcomes in healthcare, and it is a familiar and intuitive approach for clinicians, but machine learning has the potential to improve predictive performance in the orthopaedics [13]. Random forest and logistic regression have been used in prior literature on the readmission prediction [5].
3. Dataset availability of predictors: administrative database, or only in the registry. The rationale for this was that a model using administrative data could potentially be integrated into the hospital’s information technology system for automatic data processing.
4. Variable selection method: high importance in Delphi and focus group, high and moderate importance in Delphi and focus group, or systematic review predictors. A comparison of the different predictor selection approaches was included because readmission is a complex phenomenon with many potentially influential predictors [14]. Therefore, comparing a variety of approaches increased the likelihood of developing a predictive model with strong predictive performance as well as clinical relevance [8].

To reduce overfitting, strategies were employed such that there were less than 10 events (readmissions) per variable [15]. For logistic regression, the least absolute shrinkage and selection operator was used. For random forest, models were retrained using only the highest-ranked predictors according to variable importance factors.

Missing data were considered missing at random except for Veteran’s RAND 12-item health survey (VR-12) [16] scores, which were only collected routinely from 1 January 2006. Variables with more than 20% missing data were excluded [17]. For the remaining variables, k-nearest neighbours imputation (*k* = 5 nearest neighbours) was used because it is considered adequate for the purpose of prediction [18] and performs well for ≤ 20% missing data [17].

To test the impact of *k*-nearest neighbours’ imputation on model performance, two logistic regression models, with the least absolute shrinkage and selection operator, were trained to evaluate alternative strategies for handling missingness in the VR-12 variables. One model was trained with all predictors except for the VR-12 variables. The other model was trained with all predictors using data from 1 January 2006 onwards. Again, testing set data were kept separate from training set data in every step of the model development and evaluation processes.

A version of the registry dataset was generated without merging with the administrative dataset to determine whether the greater number of events (readmissions) available in the registry improved the predictive performance [19]. Whereas the administrative database only includes data from 1 July 2002 onwards, the registry contains data from 1998. The predictor selection method and model architecture for the best-performing model overall were applied to this registry-only dataset.

Risk factors were selected from the systematic review and meta-analysis [14] carried out by the authors of this study for which there was moderate- or high-quality evidence and which correlated with the readmission. To utilise the knowledge of clinicians [8], a modified Delphi survey and focus group study was carried out [20]. Variables selected for the model were those with a high-importance vote by a simple majority of ≥ 50%. Predictors voted as high-importance in the Delphi survey, despite lack of systematic review evidence for it being a readmission risk factor, included the following: preoperative patient-reported pain level, dementia, intensive care unit/high dependency unit admission prior to discharge, and return to theatre prior to discharge. Dementia was the only one of these predictors which has been investigated in the literature, and it did not increase the risk of readmission. The following predictors were correlated with readmission in the literature but did not receive a high-importance vote in the Delphi survey: number of prior emergency department presentations (12 months), age, sex, low socioeconomic status, historical knee procedures, depression, diabetes, history of cancer, hypertension, chronic kidney disease, anaemia, coagulopathy, body mass index, arrhythmia, and peripheral vascular disease. There is also evidence that length of stay is correlated with the readmission risk [21], but it did not receive a majority high-importance vote.

The Supplementary file, which has its own table of contents for ease of navigation, contains the full list of predictors (Tables S1–S3). Table S4 contains a list of all readmission prediction models developed in the primary analysis stage of this study. Table S5 depicts the amount of missingness in each variable.

### Outcome evaluation

The majority of captured readmissions were to the index hospital where the TKA procedure took place. However, the registry captures some non-index institution readmissions based on patient self-report at the routine six-week follow-up appointment. Details of the data collection and quality control processes carried out to ensure accurate capture of readmissions in accordance with these criteria have been described previously [10].

Model discrimination was measured using the area under the receiver operating characteristic curve (AUC-ROC) [22]. A perfect classifier has an AUC-ROC of 1, while random guessing yields an AUC-ROC of 0.5.

Calibration was evaluated on the test set by visual inspection of the calibration curve [23] and numerical evaluation using the Integrated Calibration Index (ICI) [24]. A perfectly calibrated model has an ICI of 0.

Two existing 30-day readmission risk prediction models, LACE + score [25] and Ali et al. [26], were compared to the bespoke models developed in this study.

A logistic regression model, and a random forest model, trained on all predictors considered throughout the model development process were also developed and fully evaluated.

The predictability of the most common causes of readmission was also compared to the prediction of readmission as an independent outcome.

Patients with and without missing data for variables which had ≥ 10% missing data were also compared according to baseline demographics and readmission rate.

The best-performing model, in terms of discriminative performance, at initial consultation and discharge was a random forest model trained on systematic review predictors using the combined (registry + administrative) dataset. These models were fully evaluated in the Results section.

### Analysis

All statistical analyses were performed using R (v4.1.1) [27]. The packages used are listed in Table S6 (Supplementary file).

To test the impact of the selected strategy for handling missing data on model performance, sensitivity analyses were conducted using different strategies for variables with a large proportion of missing data. The initial consultation logistic regression model using systematic review variables had the best calibration of all readmission prediction models in this study.

## Results

**Fig. 1:**
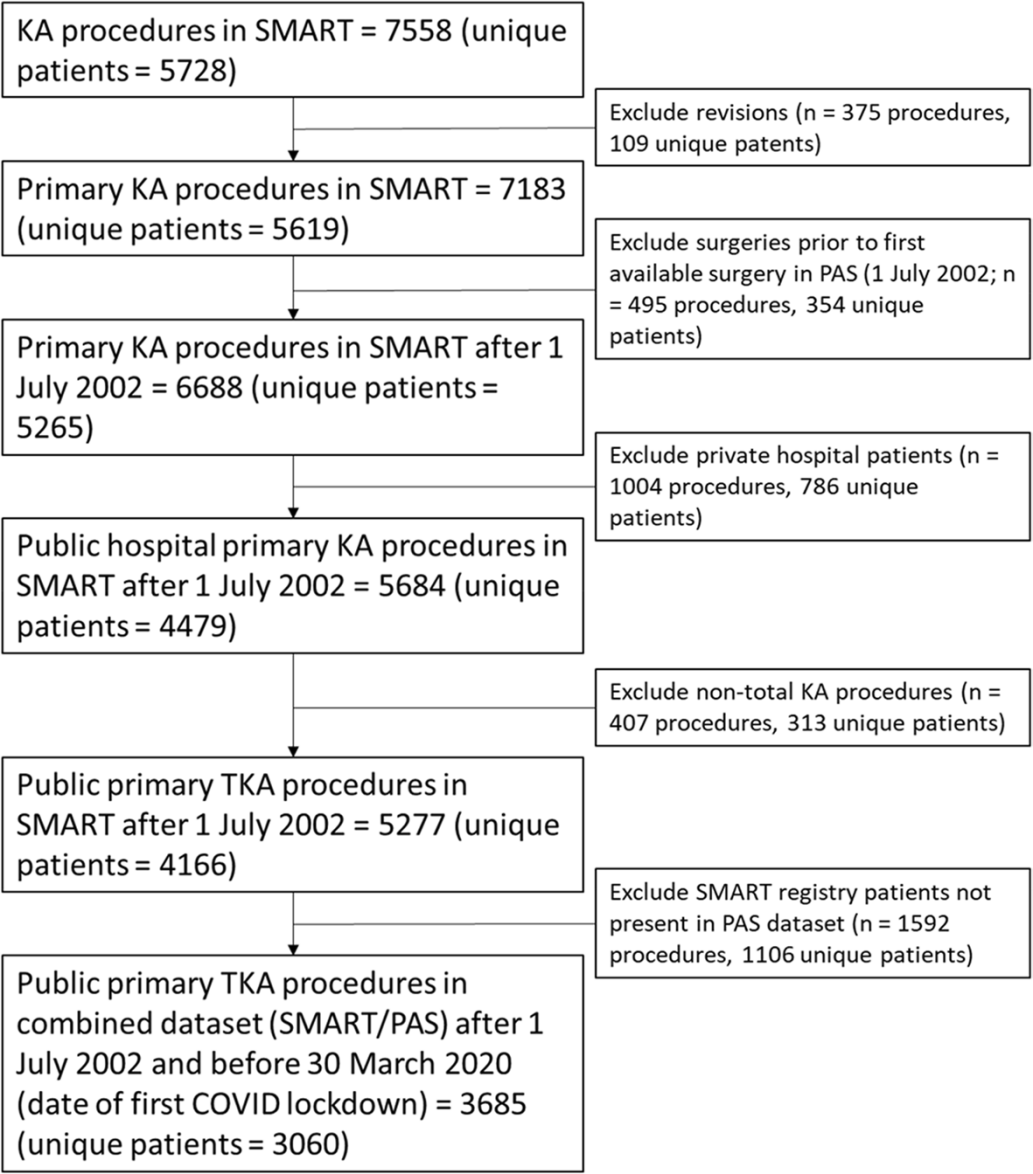
Cohort generation flow diagram. (SMART registry: St Vincent’s Melbourne Arthroplasty Outcomes registry; KA: Knee Arthroplasty; TKA: Total Knee Arthroplasty; PAS: Patient Administration System)

Figure 1 depicts a flowchart of patients included in the final analysis cohort. The date range was restricted to surgeries performed prior to 30 March 2020.

The readmission rate was 6.811%. Tables 1, 2 and 3 contain summary statistics for predictors included in this study. Table 1 contains demographics and patient-reported variables, Table 2 contains comorbidities, and Table 3 contains variables related to healthcare utilisation and the index of hospital admission.

**Table 1:** Summary statistics and comparison between readmitted and non-readmitted patients: Demographics and patient-reported variables

**Feature**	**Non-readmitted cases (*n* = 3434)**	**Readmitted cases (*n* = 251)**	***P*-value**^**d**^
**Demographics**			
Age^a^, mean (SD)	69.539 (8.8)	69.956 (8.9)	0.472
Sex^a^, % female	63.8%	61.0%	0.397
BMI^a^, mean (SD)	33.1 (6.4)	34.3 (7.8)	0.017
Smoking^a^	269 (7.8%)	21 (8.4%)	0.856
**Low SES**			
Pensioner card	1702 (49.6%)^b^	152 (60.6%)	0.001
SEIFA score			0.045
1	374 (10.9%)^a^	19 (7.6%)	
2	228 (6.6%)^a^	18 (7.2%)	
3	273 (8.0%)^a^	11 (4.4%)	
4	289 (8.4%)^a^	17 (6.8%)	
5	404 (11.8%)^a^	26 (10.4%)	
6	264 (7.7%)^a^	19 (7.6%)	
7	589 (17.2%)^a^	45 (17.9%)	
8	371 (10.8%)^a^	30 (12.0%)	
9	450 (13.1%)^a^	41 (16.3%)	
10	192 (5.6%)^a^	25 (10.0%)	
**Poor access to post-op care: lives far from hospital, lack of access to allied health support, lack of access to telehealth support**^**a**^			0.049
Major cities in Australia	2645 (77.0%)	208 (82.9%)	
Inner regional Australia	649 (18.9%)	38 (15.1%)	
Outer regional or remote Australia	131 (3.8%)	4 (1.6%)	
Missing	9 (0.3%)	1 (0.4%)	
**Patient-related biopsychosocial: lower education level, poor health literacy, non-English speaking**			0.567
Interpreter required	567 (16.5%)^a^	37 (14.7%)	
Missing	31 (0.9%)	5 (2.0%)	
**Patient-reported variables**			
Preoperative patient-reported level of function^a^, mean (SD)			
Mental function	44.4 (15.1)	42.7 (16.3)	0.133
Physical function	24.7 (7.8)	23.9 (7.5)	0.157
Preoperative patient-reported pain levels^c^			0.330
One	22 (0.6%)	1 (0.4%)	
Two	125 (3.6%)	6 (2.4%)	
Three	450 (13.1%)	26 (10.4%)	
Four	1375 (40.0%)	106 (42.2%)	
Five	960 (28.0%)	84 (33.5%)	
Missing	502 (14.6%)	28 (11.2%)	

Categorical variables were compared using the chi-squared test, or Fisher’s exact test in cases of counts below 10

*SD* Standard deviation, *BMI* Body mass index, *SEIFA* Socioeconomic Indexes for Areas [28], *SES* socioeconomic status

^a^Variable derived from SMART registry

^b^Variable derived from the administrative database

^c^ “How much did pain interfere with your normal work?”: One = Not at all; Two = A little bit; Three = Moderately; Four = Quite a bit; Five = Extremely

^d^Continuous variables were compared using Student’s *t*-test

**Table 2:** Summary statistics and comparison between readmitted and non-readmitted patients: Comorbidities

**Feature**	**Non-readmitted cases (*n* = 3434)**	**Readmitted cases (*n* = 251)**	***P*-value**^**b**^
**Comorbidities**			
Hypertension^a^	2289 (66.7%)	165 (65.7%)	0.819
Peripheral vascular disease^a^	129 (3.8%)	13 (5.2%)	0.337
Diabetes^a^	Diabetes = 757 (22.0%)	Diabetes = 70 (27.9%)	0.080
	Diabetes with end-organ damage = 15 (0.4%)	Diabetes with end-organ damage = 1 (0.4%)	
Coagulopathy^a^	19 (0.6%)	0	0.636
Charlson Comorbidity Index^a^	Zero = 1780 (51.8%)	Zero = 113 (45.0%)	0.026
	One = 956 (27.8%)	One = 70 (27.9%)	
	≥ Two = 698 (20.3%)	≥ Two = 68 (27.1%)	
CHF^a^	100 (2.9%)	10 (4.0%)	0.334
Liver disease^a^	85 (2.5%)	8 (3.2%)	0.528
Depression^a^	394 (11.5%)	36 (14.3%)	0.206
Previous stroke^a^	209 (6.2%)	21 (8.4%)	0.191
Anaemia^a^	59 (1.7%)	9 (3.6%)	0.047
History of cancer^a^	340 (9.9%)	31 (12.4%)	0.256
High risk of infection: immunocompromised state, active IVDU, infection in other primary joint replacement^a^	1466 (42.7%)	126 (50.2%)	0.024
CKD^a^	125 (3.6%)	16 (6.4%)	0.039
Arrhythmia^a^	18 (0.5%)	0	0.630
Pulmonary disease^a^	174 (5.1%)	23 (9.2%)	0.008
Dementia^a^	12 (0.3%)	0	1
Substance abuse^a^	59 (1.7%)	3 (1.2%)	0.798

Categorical variables were compared using the chi-squared test, or Fisher’s exact test in cases of counts below 10

*SD* Standard deviation, *SEIFA* Socioeconomic Indexes for Areas [28], *IVDU* Intravenous drug use, *CKD* Chronic kidney disease

^a^Variable derived from SMART registry

^b^Continuous variables were compared using Student’s* t*-test

**Table 3:** Summary statistics and comparison between readmitted and non-readmitted patients: Healthcare utilisation and index hospital admission

**Feature**	**Non-readmitted cases (*n* = 3434)**	**Readmitted cases (*n* = 251)**	***P*-value**^**c**^
**Prior healthcare utilisation**			
Increasing number of previous admissions^b^	Zero = 3223 (93.9%)	Zero = 230 (91.6%)	0.234
	One = 116 (3.4%)	One = 10 (4.0%)	
	Two = 50 (1.5%)	Two = 4 (1.6%)	
	≥ Three = 45 (1.3%)	≥ Three = 7 (2.8%)	
Number of prior ED presentations (12 months)^b^	Zero = 3298 (96.0%)	Zero = 234 (93.2%)	0.083
	One = 81 (2.4%)	One = 11 (4.4%)	
	≥ Two = 55 (1.6%)	≥ Two = 6 (2.4%)	
Historical knee procedures^b^	Zero = 1234 (35.9%)	Zero = 82 (32.7%)	< 0.001
	One = 1324 (38.6%)	One = 77 (30.7%)	
	Two = 760 (22.1%)	Two = 51 (20.3%)	
	≥ Three = 116 (3.4%)	≥ Three = 41 (16.3%)	
**Variables related to index hospital admission**			
In-hospital complication (any) during index admission^a^	477 (13.9%)	76 (30.3%)	< 0.001
ICU/HDU admission during index admission^b^	Zero = 3303 (96.2%)	Zero = 237 (94.4%)	0.109
	One = 64 (1.9%)	One = 4 (1.6%)	
	≥ Two = 67 (2.0%)	Two = 10 (4.0%)	
Return to theatre during index admission^b^	10 (29.1%)	6 (2.4%)	< 0.001
Length of stay in days, mean (SD)^a^	8.993 (4.4)	11.283 (8.9)	< 0.001
Duration of operation in minutes, mean (SD)^b^	119.796 (34.5)	120.928 (36.3)	0.632
Wound class (not clean)^b^	7 (20.4%)	0	1
Transfusion during surgery in number of packed red blood cells, mean (SD)^a^	Zero = 3110 (90.6%)	Zero = 212 (84.5%)	< 0.001
	One = 63 (1.8%)	One = 5 (1.0%)	
	Two = 206 (6.0%)	Two = 21 (8.4%)	
	≥ Three = 55 (1.6%)	≥ Three = 13 (5.2%)	

Categorical variables were compared using chi-squared test, or Fisher’s exact test in cases of counts below 10

*SD* Standard deviation, *BMI* Body mass index, *SEIFA* Socioeconomic Indexes for Areas [28], *ED* Emergency department, *ICU* Intensive care unit, *HDU* High dependency unit

^a^Variable derived from SMART registry

^b^Variable derived from administrative database

^c^Continuous variables were compared using Student’s *t*-test

### Results for primary readmission prediction models

**Fig. 2:**
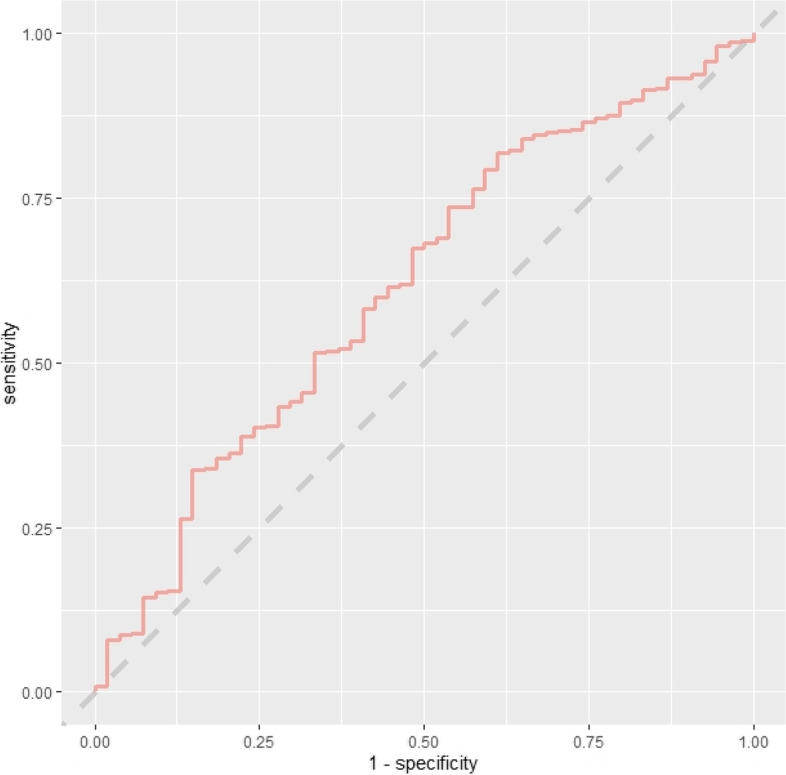
ROC curve—initial consultation random forest model trained on systematic review predictors using the combined (registry + administrative) dataset

**Fig. 3:**
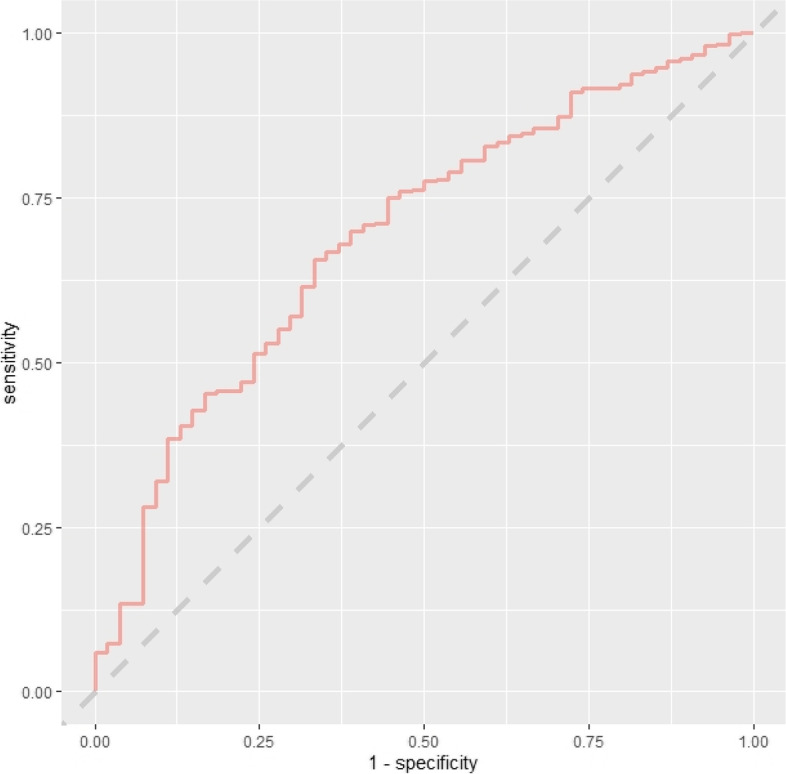
ROC curve—discharge random forest model trained on systematic review predictors using the combined (registry + administrative) dataset

The training set performance of all models developed in the main readmission prediction model development process is contained in Table S7 (Supplementary file). Comparison of baseline demographics and readmission rate for variables with ≥ 10% missingness-missing vs. non-missing are contained in Tables S8–S10. No variables had > 20% missing data. The initial consultation random forest model achieved an AUC-ROC of 0.617 (95% CI 0.538–0.696). The discharge random forest model achieved an AUC-ROC of 0.692 (95% CI 0.621–0.764). ROC curves for these models evaluated on the test set are presented below (Figs. 2 and 3). Variable importance factors for these models are contained in Tables S11 and S12, along with training set ROC curves in Figs. S1 and S2 (Supplementary file).

**Fig. 4:**
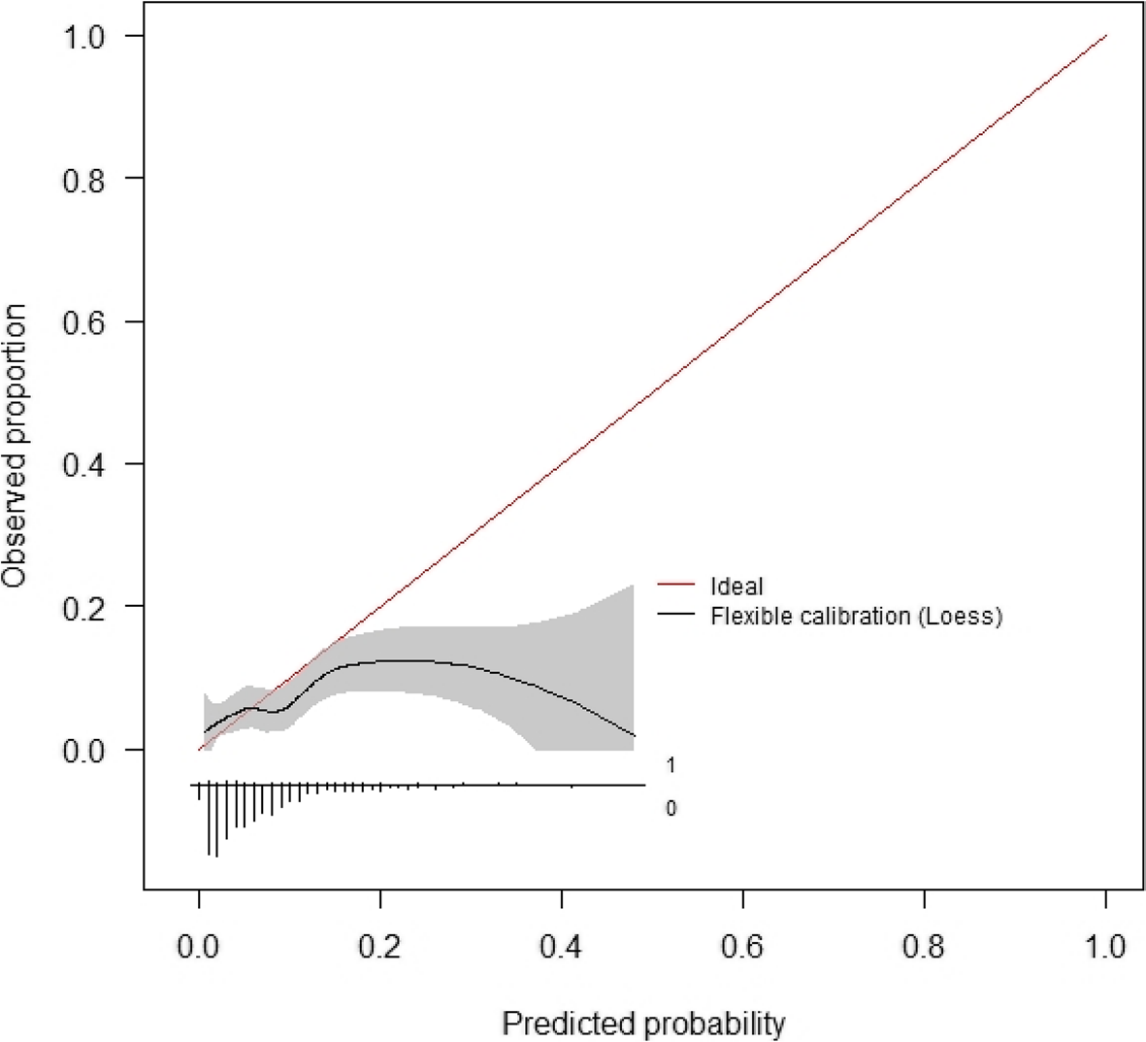
Calibration curve—initial consultation random forest model trained on systematic review predictors using the combined (registry + administrative) dataset

**Fig. 5:**
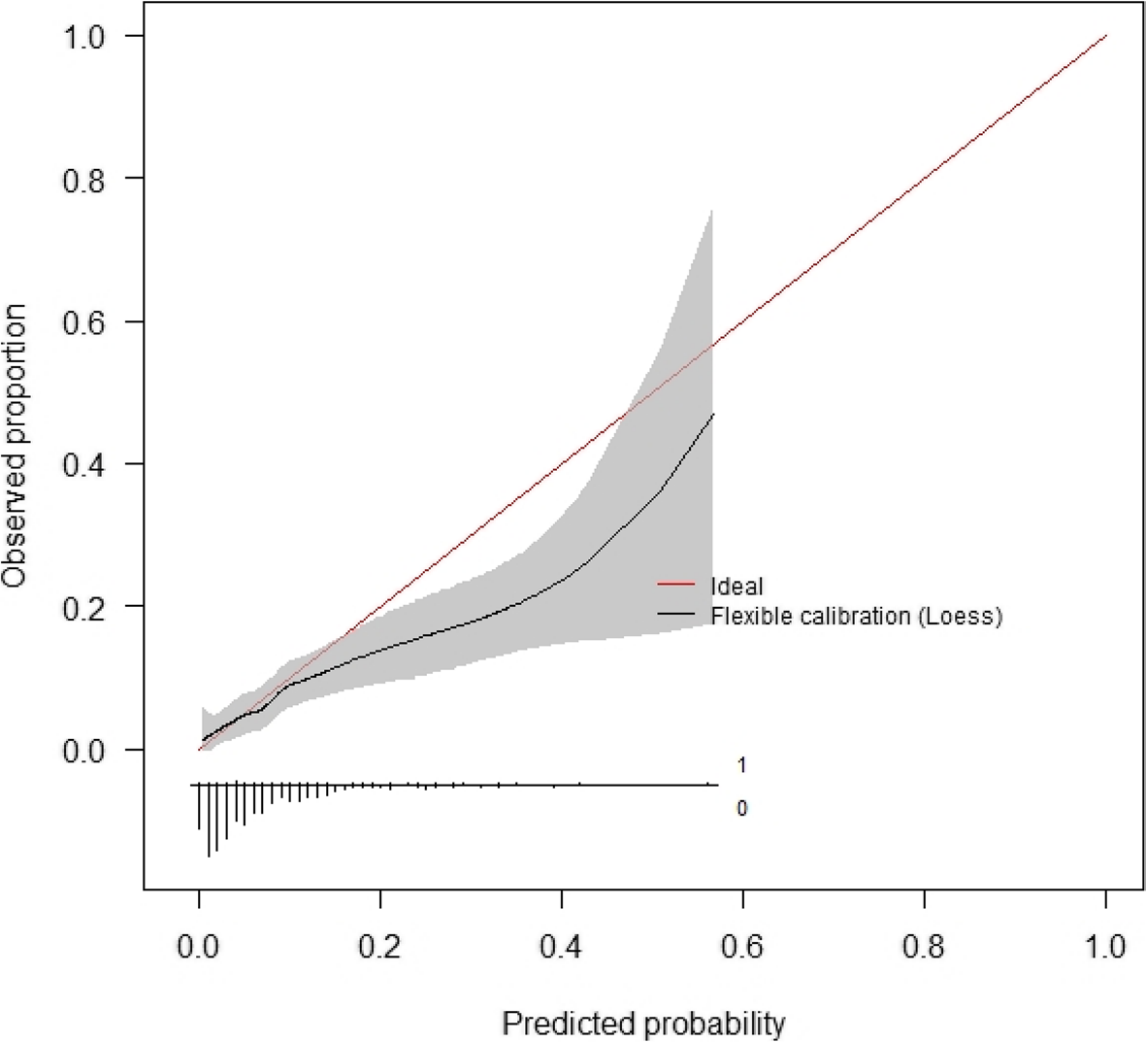
Calibration curve—discharge random forest model trained on systematic review predictors using the combined (registry + administrative) dataset

**Fig. 6:**
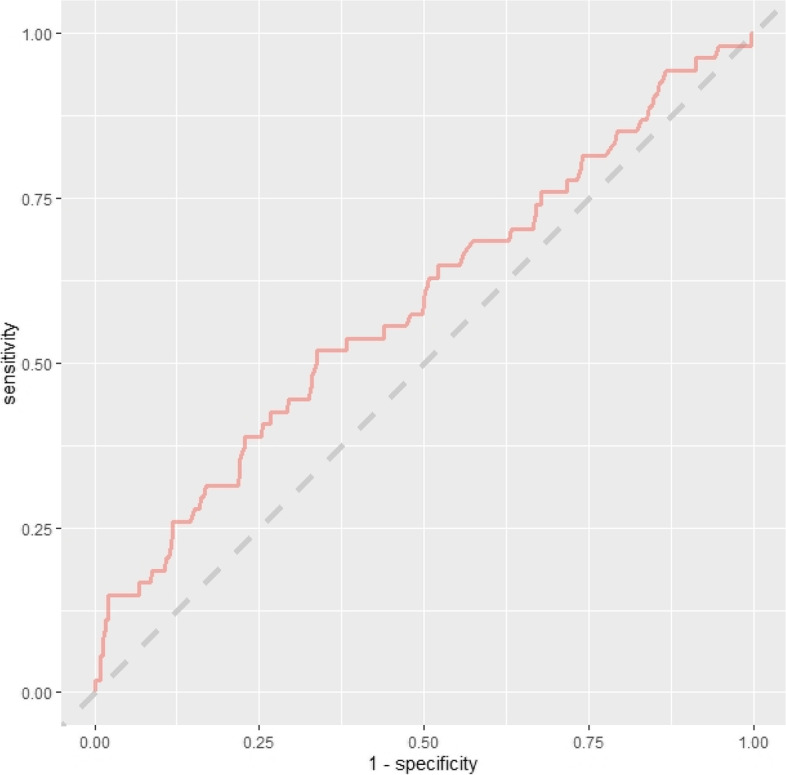
ROC curve—initial consultation logistic regression model using systematic review predictors in the administrative dataset

**Fig. 7:**
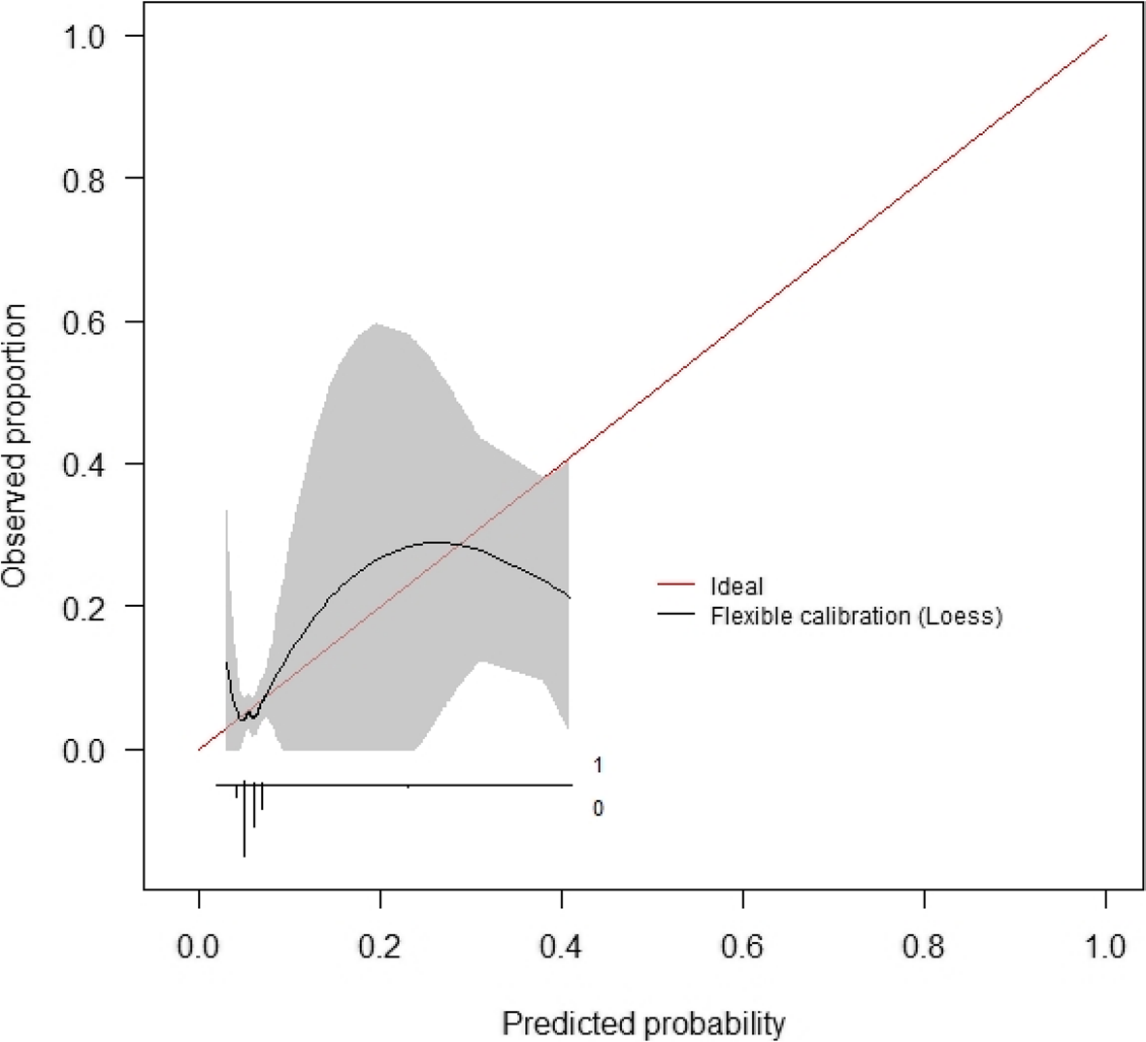
Calibration curve—initial consultation logistic regression model using systematic review predictors in the administrative dataset

Calibration curves for these models are presented below (Figs. 4 and 5). The initial consultation random forest model achieved an ICI of 0.031. The discharge random forest model achieved an ICI of 0.019. The appearance of these calibration curves indicates an overestimation of risk. Precision-recall curves (Figs. S3–S4) and additional performance metrics (Table S13) are available for these models in the Supplementary file. The best-calibrated readmission prediction model was a logistic regression model trained on variables available in the administrative dataset at the initial consultation. These predictors were age, sex, hospital admissions and emergency presentations in the past 12 months, socioeconomic status, and the number of prior knee procedures. The ROC curve and calibration curve for this model are presented below (Figs. 6 and 7, respectively). AUC-ROC was 0.589 (95% CI 0.506–0.673), and ICI was 0.012. The full performance evaluation of this model is available in the Supplementary file (Figs. S5–S10, and Tables S14–S17).

AUC-ROC of 0.583 (0.545–0.620) was attributed to LACE + , while 0.563 (0.525–0.602) for its presented in Ali et al. [26]. ICI was exhibited in LACE + and Ali et al. [26] at 0.642 and 0.100, respectively. A full performance evaluation has also been performed (in Supplementary file): these previously developed models from prior literature (Figs. S11–S16, and Tables S18–S21), the random forest model trained on all predictor’s models (Figs. S17–S28, and Tables S22–S28), and the logistic regression model trained on all predictors (Figs. S29–S36, Tables S29–S34).

Predictor summary statistics for the registry dataset not merged with administrative data are contained in the Supplementary file (Table S35), along with the amount of missingness per variable (Table S36) and the dataset cohort creation flow diagram (Fig. S37). Full performance evaluation of the random forest models trained on this dataset is also contained (Figs. S38–S45, and Tables S37–S42). The complication-specific models demonstrated comparable performance to the readmission prediction models, albeit generally with slightly better discriminative performance.

We also evaluated the outcome definitions and predictors for each readmission-related complication from the literature (Table S43), causes of readmission in this study cohort (Table S44), further information on outcome variable generation (Table S45) and predictor variable generation (Tables S46–S51), predictor variable preparation and missingness (Table S52), comparison of baseline characteristics for participants with ≥ 10% missing data for given variable (Table S53). Baseline characteristics were also compared for patients who experienced each complication, and those who did not (Tables S54–S60). Full performance evaluations for all complication-specific models are also contained (Figs. S46–S83, and Tables S61–S81).

**Fig. 8:**
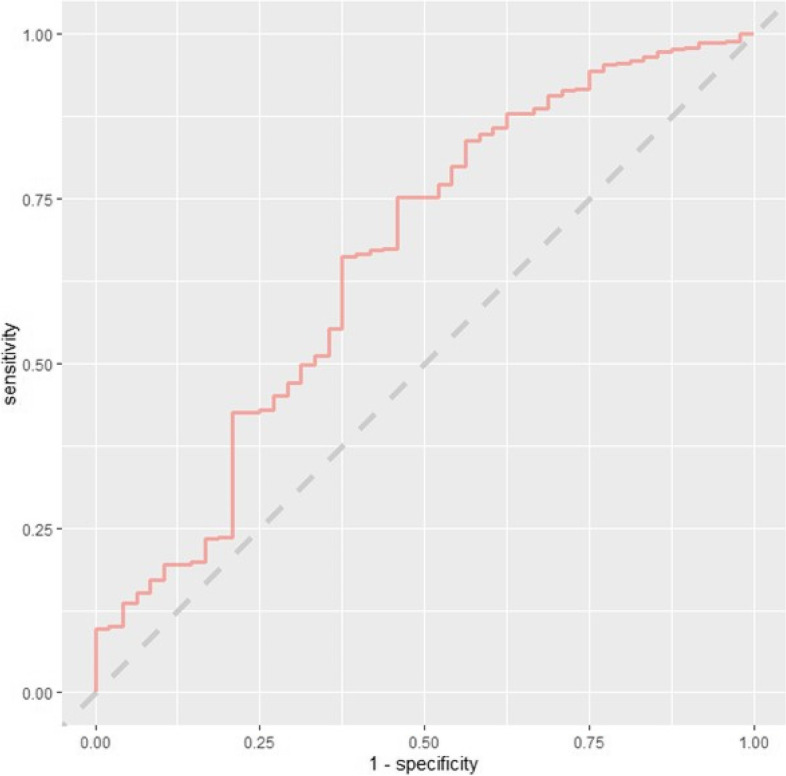
ROC curve – discharge random forest model using all study predictors in the combined dataset to predict any complication associated with readmission

**Fig. 9:**
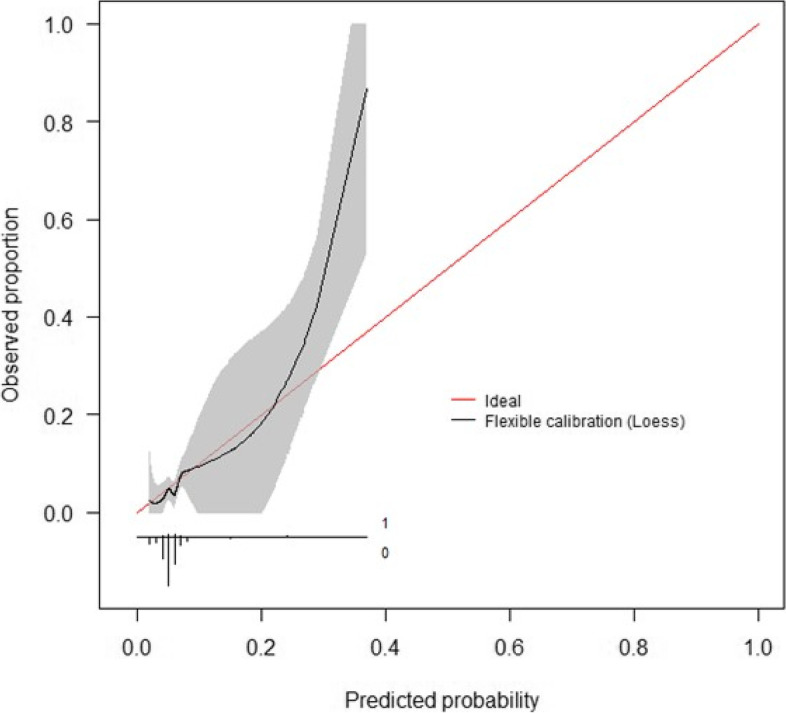
Calibration curve – initial consultation logistic regression model using all study predictors in the administrative dataset to predict any complication associated with readmission

The model developed using all study predictors for the combined outcome variable indicating any complication associated with readmission achieved an AUC-ROC of 0.658 (0.570–0.746). This was an improvement over the readmission prediction models, however, discriminative performance still falls short of the commonly accepted AUC-ROC threshold of 0.7 [29]. The ROC curve for this model is presented below (Fig. 8). The best-calibrated complication-specific model was a logistic regression model which achieved an ICI of 0.012, indicating good calibration overall, but the calibration curve clearly shows an underestimation of risk at higher predicted probabilities. The calibration curve for this model is presented below (Fig. 9).

The training set AUC-ROC for the logistic regression model with all predictors was 0.677 with* k*-nearest neighbours’ imputation, 0.655 for all predictors using data from 1 January 2006 onwards, and 0.677 for all predictors except for VR-12 scores.

## Discussion

In summary, the discriminative performance of all models was poor, although machine learning models outperformed logistic regression to a small degree. However, the logistic regression model trained on administrative data available in the clinical environment, using systematic review predictors available at an initial consultation, was reasonably well calibrated. This is useful because it suggests that interventions to mitigate or respond to readmission risk could be implemented at a much earlier point in time than at discharge following TKA surgery [30]. These findings are in keeping with prior literature demonstrating the difficulty of developing predictive models capable of distinguishing between readmitted and non-readmitted patients in various clinical populations, especially following surgery and specifically TKA [5]. Comparable performance to the primary model development procedure was achieved in the sensitivity analysis pertaining to different strategies for handling missingness in the VR-12 data, providing support for the use of *k*-nearest neighbours imputation.

One particular type of machine learning which has received a large amount of attention in the literature pertaining to the prediction of surgical outcomes, including in orthopaedics and knee arthroplasty, specifically, is the deep learning [4]. This type of machine learning has demonstrated potential in terms of improved discriminative performance for outcomes post-TKA [4], however, it generally requires a high volume of complex data to fully unlock its potential [7]. In many cases, deep learning is not guaranteed to improve predictive performance compared with other modelling techniques [31]. As data capture continues to expand in orthopaedics, it is possible there will be improvements in predictive performance, which in turn could improve the quality of a shared clinical decision-making [32]. One thing is clear: artificial intelligence and machine learning are here to stay in the orthopaedic field [32, 33]. It is important to temper expectations [34] and focus more on the human interaction between patient and clinician as they work together to achieve the best possible surgical outcome [33].

Some risk factors were consistently associated with readmission. Presented in this section are the predictors with the largest regression coefficients in the LACE + model and the model developed by Ali et al. [26], compared with the strongest predictors in the bespoke models developed in this study. In both of these models, length of stay and number of prior emergency department visits were among the top five strongest predictors. Length of stay was also consistently among the top five strongest predictors in the models developed in the current study, while the number of prior emergency department visits was one of the strongest predictors in the initial consultation administrative database model that exhibited the best overall calibration. Older age was the other strongest predictor in the Ali et al. model, and age as a continuous predictor was also among the strongest predictors in the random forest models developed in the current study which demonstrated the best discriminative performance. On the other hand, the remaining top predictors in the LACE + model were urgent admissions in the previous year, Charlson Comorbidity Index, and the male sex. Charlson Comorbidity Index was also among the strongest predictors in the main random forest model developed using data available at the initial consultation in this study, with length of stay replacing it in the model developed using the same model architecture using predictors available at discharge. Admissions in the past 12 months, though not specifically urgent admissions, was one of the strongest predictors in the initial consultation administrative database model that achieved the best overall calibration. Male sex was not a strong predictor in any of the models developed in this study. There were also newly identified predictors for readmission: number of historical knee procedures, socioeconomic status, and body mass index (BMI). There was evidence from the systematic review and meta-analysis [14] that these risk factors correlated with readmission, however, BMI and low socioeconomic status only received a majority vote of moderate importance in the Delphi survey, and the number of historical knee procedures received a majority low importance vote [20].

Models trained on all predictors had similar performance to primary study models. This suggests that using clinical insight instead of purely relying on statistical or machine learning predictor selection has value in terms of increasing clinical relevance/applicability without sacrificing predictive performance. The model trained only on clinical registry data also performed similarly to the primary models developed using both administrative and registry data.

The models developed in prior studies did not perform well on the datasets used in this study. These were the LACE + score [25] and the model developed by Ali et al. [26]. In accordance with Stessel et al. [35], compromises had to be made when applying these models because not all variables were available in the dataset used for this study and some proxy variables had to be generated based on what was available in the datasets used in this study. These models performed poorly on discrimination and calibration. These findings are in keeping with prior literature in which bespoke models have outperformed existing models such as LACE [36]. Important considerations when interpreting the poor performance of these models include the following: the current study was not a formal external validation study, there was incomplete variable availability, and both models were developed outside Australia (Ali et al. in the UK, LACE + in Canada), the Ali et al.’s model was developed for risk factor identification rather than prediction, and the LACE + model was not developed specifically for TKA patients.

The most common causes of readmission were identified from prior literature [37, 38]. These were surgical site infection, venous thromboembolism, joint-specific complications, gastrointestinal complications, cardiac complications, and infection (non-surgical site). Causes of readmission in this study cohort are listed in Table S44 (Supplementary file). These outcome variables were generated based on definitions derived from the literature and the variables available in the data for each outcome category. There are multiple advantages to using a general readmission prediction model implemented alongside complication-specific models. It enables the identification of patients with high readmission risk and can provide insight into their risk of specific complications. It also facilitates the identification of patients who are at high risk for readmission but not for any specific common cause. These readmissions might be unexpected from a clinical point of view but nonetheless can be anticipated and prepared for through post-discharge follow-up. In line with the readmission prediction model evaluation, the best-calibrated complication prediction model was described. This logistic regression model predicted any complication using all predictors in this study available in the administrative database at the initial consultation: sex, age, rurality, socioeconomic status, number of hospital admissions and emergency presentations in the past 12 months, and number of prior knee procedures.

The most well-calibrated models developed in this study, for both readmission prediction and prediction of complications associated with readmission, were developed using data captured routinely in the live clinical environment available at the initial consultation. This facilitates automated data processing by the predictive model. The result can be displayed to the patients and surgeons alongside the incidence for the whole cohort of patients at the institution to compare the patient’s risk to that of other patients. Well-calibrated models that do not have strong discriminative performance can still be useful in shared decision-making, due to their ability to calculate individualised probabilistic estimates of readmission [39]. Provided here is an example of how the model can be used in the process of shared clinical decision-making. Imagine there is a patient with a predicted probability of 0.33 for readmission, using the best-calibrated model developed for readmission in this study. The highest predicted probability calculated by this model is 0.4 (see the *x*-axis of Fig. 7), so a predicted probability of 0.33 is towards the higher end of possible individualised predicted probabilities. The clinician might opt to provide the percentage value, 33%, or a natural frequency, in this case, 1 in 3, to describe the predicted probability and explain that this is the proportion of patients just like them who would be readmitted following TKA surgery. They can inform the patient that this is almost five times as high as the average readmission rate for the cohort in this study, which was 6.8% or approximately 1 in 15. The patient and clinician can then decide whether they believe the patient’s discharge planning should include flagging them for additional follow-up at one or more checkpoints within the 30 days following discharge after TKA surgery [40]. The output of calibrated predictive models such as that developed in this study should not dictate decisions made between patient and clinician, but should instead empower both parties in the shared clinical decision-making process which still requires intuition and consideration of the human elements that cannot be captured by a statistical tool [41].

Strengths of this study include a comprehensive predictor selection strategy which involved clinical input and machine learning while prioritising model parsimony. The model development, internal validation, and evaluation processes were in line with the guidelines [9]. The models were bespoke [36] and developed on a well-described and diverse clinical population which is demographically representative of the broader Australian TKA population [10]. Comprehensive information on the data used, as well as information required by readers to apply the models in different clinical settings or replicate this process to develop their own bespoke model [42], was provided. The corresponding author can also be contacted for information and clarification if necessary. The limitations of this study include that this was a single-institution study. The only way to fully capture non-index institution readmissions would be through linkage to external datasets. The main limitation was that the model does not have strong discriminative performance, therefore it should not be used to distinguish between patients perceived to be at high risk of readmission in a binary manner. Rather, it can be used to inform decision-making given it was well-calibrated.

In order to improve the discriminative performance of the model, future work could focus on expanding data capture to facilitate the utilisation of strong predictors for readmission or associated complications in this patient population that are currently not captured in the databases available for the development of predictive models. Before being deployed, the model will need to be pilot tested in the clinical environment to determine whether it can be implemented into existing workflows.

## Conclusions

The discriminative performance of the readmission prediction and complication prediction models was poor, although machine learning models had slightly better discriminative performance than logistic regression models. The model developed using administrative data available at the initial consultation between the patient and orthopaedic surgeon was reasonably well calibrated. Models developed to predict complications commonly associated with readmission were also reasonably well-calibrated and can be used in conjunction with readmission prediction models in shared clinical decision-making.

## Supplementary Information

**Additional file 1.**

## Data Availability

Individual patient data are not publicly available. Requests for additional information can be sent to the corresponding author.

## Abbreviations

TKA: Total knee arthroplasty
SMART: St Vincent’s Melbourne Arthroplasty Outcomes
VR-12: Veteran’s RAND 12-item health survey
AUC-ROC: Area under the receiver operating characteristic curve
ICI: Integrated Calibration Index
HDU: High dependency unit
LACE: Length of stay (L), acuity of the admission (A), comorbidity of the patient (C), and emergency department use in the duration of 6 months before admission (E)
BMI: Body mass index

## References

[1] Jencks SF, Williams MV, Coleman EA (2009). Rehospitalizations among patients in the Medicare fee-for-service program. N Engl J Med.

[2] ACSQHC (2019). Avoidable Hospital Readmissions: report on Australian and International indicators, their use and the efficacy of interventions to reduce readmissions.

[3] McIlvennan CK, Eapen ZJ, Allen LA (2015). Hospital readmissions reduction program. Circulation.

[4] Lopez CD, Gazgalis A, Boddapati V, Shah RP, Cooper HJ, Geller JA (2021). Artificial learning and machine learning decision guidance applications in total hip and knee arthroplasty: a systematic review. Arthroplast Today.

[5] Futoma J, Morris J, Lucas J (2015). A comparison of models for predicting early hospital readmissions. J Biomed Inform.

[6] Ashfaq A, Sant’Anna A, Lingman M, Nowaczyk S (2019). Readmission prediction using deep learning on electronic health records. J Biomed Inform.

[7] Hinterwimmer F, Lazic I, Suren C, Hirschmann MT, Pohlig F, Rueckert D, Burgkart R, von Eisenhart-Rothe R. Machine learning in knee arthroplasty: specific data are key—a systematic review. Knee Surgery, Sports Traumatology, Arthroscopy. 2022;30(2):376-88. 10.1007/s00167-021-06848-6 PMC8866371 35006281

[8] Steyerberg EW. Clinical prediction models. CH (Switzerland): Springer Nature Switzerland AG; 2019. 10.1007/978-3-030-16399-0.

[9] Collins GS, Reitsma JB, Altman DG, Moons KG (2015). Transparent reporting of a multivariable prediction model for individual prognosis or diagnosis (TRIPOD): the TRIPOD statement. J Br Surg.

[10] Gould D, Thuraisingam S, Shadbolt C, Knight J, Young J, Schilling C (2021). Cohort profile: the St Vincent’s Melbourne Arthroplasty Outcomes (SMART) Registry, a pragmatic prospective database defining outcomes in total hip and knee replacement patients. BMJ Open.

[11] Refaeilzadeh P, Tang L, Liu H (2009). Cross-validation. Encycl Database Syst.

[12] Manning DW, Edelstein AI, Alvi HM (2016). Risk prediction tools for hip and knee arthroplasty. J Am Acad Orthop Surg.

[13] Oosterhoff JH, Gravesteijn BY, Karhade AV, Jaarsma RL, Kerkhoffs GM, Ring D (2022). Feasibility of machine learning and logistic regression algorithms to predict outcome in orthopaedic trauma surgery. JBJS.

[14] Gould D, Dowsey MM, Spelman T, Jo O, Kabir W, Trieu J (2021). Patient-related risk factors for unplanned 30-day hospital readmission following primary and revision total knee arthroplasty: a systematic review and meta-analysis. J Clin Med.

[15] Pavlou M, Ambler G, Seaman SR, Guttmann O, Elliott P, King M (2015). How to develop a more accurate risk prediction model when there are few events. BMJ (Clinical research ed).

[16] Kazis LE, Miller DR, Skinner KM, Lee A, Ren XS, Clark JA (2006). Applications of methodologies of the Veterans Health Study in the VA healthcare system: conclusions and summary. J Ambul Care Manag.

[17] Emmanuel T, Maupong T, Mpoeleng D, Semong T, Mphago B, Tabona O (2021). A survey on missing data in machine learning. J Big Data.

[18] Choudhury A, Kosorok MR. Missing data imputation for classification problems. arXiv preprint arXiv:200210709. 2020.

[19] Van Calster B, Nieboer D, Vergouwe Y, De Cock B, Pencina MJ, Steyerberg EW (2016). A calibration hierarchy for risk models was defined: from utopia to empirical data. J Clin Epidemiol.

[20] Gould D, Dowsey M, Spelman T, Bailey J, Bunzli S, Rele S (2023). Established and novel risk factors for 30-day readmission following total knee arthroplasty: a modified Delphi and focus group study to identify clinically important predictors. J Clin Med.

[21] Mahajan SM, Nguyen C, Bui J, Kunde E, Abbott BT, Mahajan AS (2020). Risk factors for readmission after knee arthroplasty based on predictive models: a systematic review. Arthroplast Today.

[22] Steyerberg EW, Vergouwe Y (2014). Towards better clinical prediction models: seven steps for development and an ABCD for validation. Eur Heart J.

[23] Austin PC, Steyerberg EW (2014). Graphical assessment of internal and external calibration of logistic regression models by using loess smoothers. Stat Med.

[24] Austin PC, Steyerberg EW (2019). The Integrated Calibration Index (ICI) and related metrics for quantifying the calibration of logistic regression models. Stat Med.

[25] van Walraven C, Wong J, Forster AJ (2012). LACE+ index: extension of a validated index to predict early death or urgent readmission after hospital discharge using administrative data. Open Med.

[26] Ali AM, Loeffler MD, Aylin P, Bottle A (2019). Predictors of 30-day readmission after total knee arthroplasty: analysis of 566,323 procedures in the United Kingdom. J Arthroplasty.

[27] R Core Team. R: A language and environment for statistical computing. Vienna, Austria: R Foundation for Statistical Computing; 2013. http://www.R-project.org/.

[28] Statistics ABO (2011). Socio-economic indexes for areas (SEIFA).

[29] Yang S, Berdine G (2017). The receiver operating characteristic (ROC) curve. Southwest Respir Crit Care Chron.

[30] Amarasingham R, Moore BJ, Tabak YP, Drazner MH, Clark CA, Zhang S (2010). An automated model to identify heart failure patients at risk for 30-day readmission or death using electronic medical record data. Med Care.

[31] Christodoulou E, Ma J, Collins GS, Steyerberg EW, Verbakel JY, Van Calster B (2019). A systematic review shows no performance benefit of machine learning over logistic regression for clinical prediction models. J Clin Epidemiol.

[32] Younis MU (2021). Impact of artificial intelligence integration on surgical outcome. J Dow Univ Health Sci.

[33] Kumar V, Patel S, Baburaj V, Vardhan A, Singh PK, Vaishya R (2022). Current understanding on artificial intelligence and machine learning in orthopaedics–a scoping review. J Orthop.

[34] Wellington IJ, Cote MP. Editorial Commentary: Machine Learning in Orthopaedics: Venturing Into the Valley of Despair. Arthroscopy: The Journal of Arthroscopic & Related Surgery. 2022;38(9):2767-8. 10.1016/j.arthro.2022.05.010 36064282

[35] Stessel B, Fiddelers AA, Marcus MA, van Kuijk SM, Joosten EA, Peters ML (2017). External validation and modification of a predictive model for acute postsurgical pain at home after day surgery. Clin J Pain.

[36] Yu S, Farooq F, Van Esbroeck A, Fung G, Anand V, Krishnapuram B (2015). Predicting readmission risk with institution-specific prediction models. Artif Intell Med.

[37] Curtis GL, Jawad M, Samuel LT, George J, Higuera-Rueda CA, Little BE (2019). Incidence, causes, and timing of 30-day readmission following total knee arthroplasty. J Arthroplasty.

[38] Ramkumar PN, Chu C, Harris J, Athiviraham A, Harrington M, White D (2015). Causes and rates of unplanned readmissions after elective primary total joint arthroplasty: a systematic review and meta-analysis. Am J Orthop.

[39] Munn JS, Lanting BA, MacDonald SJ, Somerville LE, Marsh JD, Bryant DM (2022). Logistic regression and machine learning models cannot discriminate between satisfied and dissatisfied total knee arthroplasty patients. J Arthroplasty.

[40] Hamar GB, Coberley C, Pope JE, Cottrill A, Verrall S, Larkin S (2017). Effect of post-hospital discharge telephonic intervention on hospital readmissions in a privately insured population in Australia. Aust Health Rev.

[41] Bonner C, Trevena LJ, Gaissmaier W, Han PK, Okan Y, Ozanne E (2021). Current best practice for presenting probabilities in patient decision aids: fundamental principles. Med Decis Making.

[42] Fujimori R, Liu K, Soeno S, Naraba H, Ogura K, Hara K (2022). Acceptance, barriers, and facilitators to implementing artificial intelligence-based decision support systems in emergency departments: quantitative and qualitative evaluation. JMIR Form Res.

