# Supplementary material for: Predicting 30-day readmission following total knee arthroplasty using machine learning and clinical expertise applied to clinical administrative and research registry data in an Australian cohort
Source: Arthroplasty. 2023 Jun 1;5:30. doi: 10.1186/s42836-023-00186-3 (PMC10234041; doi:10.1186/s42836-023-00186-3)
Supplement: Supplementary file 1 — Additional file 1. [file 42836_2023_186_MOESM1_ESM.docx]

Table of Contents

[Variable availability – Table S1 5](#_Toc131461433)

[Variable generation – Table S2 7](#_Toc131461434)

[Variable preparation – Table S3 10](#_Toc131461435)

[Models – Table S4 12](#_Toc131461436)

[Missingness per variable – Table S5 17](#_Toc131461437)

[Statistical Packages and Version Numbers – Table S6 19](#_Toc131461438)

[Training set performance of all models developed in primary analysis – Table S7 20](#_Toc131461439)

[Comparison of baseline demographics and readmission rate for variables with ≥10% missingness - missing vs non-missing (Tables S8-S10) 22](#_Toc131461440)

[Table S8: Comparison of patients missing vs not missing VR-12 mental score 22](#_Toc131461441)

[Table S9: Comparison of patients missing vs not missing VR-12 physical score 22](#_Toc131461442)

[Table S10: Comparison of patients missing vs not missing VR-12 pain score 22](#_Toc131461443)

[Main models variable importance factors (Tables S11-S12) 23](#_Toc131461444)

[Table S11: Model type: Random forest; Dataset: Combined; Timepoint: Initial consultation 23](#_Toc131461445)

[Table S12: Model type: Random forest; Dataset: Combined; Timepoint: Discharge 23](#_Toc131461446)

[Main models training set receiver operating characteristic (ROC) curves (Figures S1-S2) 25](#_Toc131461447)

[Figure S1: Model type: Random forest; Dataset: Combined; Timepoint: Initial consultation 25](#_Toc131461448)

[Figure S2: Model type: Random forest; Dataset: Combined; Timepoint: Discharge 26](#_Toc131461449)

[Main models precision-recall (PPV-sensitivity) curves (Figures S3-S4) 27](#_Toc131461450)

[Figure S3: Model type: Random forest; Dataset: Combined; Timepoint: Initial consultation 27](#_Toc131461451)

[Figure S4: Model type: Random ftablorest; Dataset: Combined; Timepoint: Discharge 28](#_Toc131461452)

[Table S13: Main models performance metrics – Initial consultation and discharge (Model type: Random forest; Dataset: Combined) 28](#_Toc131461453)

[Best administrative database models – full performance evaluation (Figures S5-S6, Tables S14-S15, Figures S7-S9, Table S16, Figure S10, Table S17) 29](#_Toc131461454)

[Figure S5: Training set ROC curve – Model type: Logistic regression; Dataset: Administrative database; Timepoint: Initial consultation; Predictors: Systematic review predictors 30](#_Toc131461455)

[Figure S6: Precision-recall (PPV-sensitivity) curve – Model type: Logistic regression; Dataset: Administrative database; Timepoint: Initial consultation; Predictors: Systematic review predictors 31](#_Toc131461456)

[Table S14: Performance metrics 31](#_Toc131461457)

[Table S15: Coefficients – Model type: Logistic regression; Dataset: Administrative database; Timepoint: Initial consultation; Predictors: Systematic review predictors 32](#_Toc131461458)

[Figure S7: Training set ROC curve – Model type: Random forest; Dataset: Administrative database; Timepoint: Discharge; Predictors: Systematic review predictors 33](#_Toc131461459)

[Figure S8: Test set ROC curve – Model type: Random forest; Dataset: Administrative database; Timepoint: Discharge; Predictors: Systematic review predictors 34](#_Toc131461460)

[Figure S9: Precision-recall (PPV-sensitivity) curve – Model type: Random forest; Dataset: Administrative database; Timepoint: Discharge; Predictors: Systematic review predictors 35](#_Toc131461461)

[Table S16: Performance metrics – Model type: Random forest; Dataset: Administrative database; Timepoint: Discharge; Predictors: Systematic review predictors 35](#_Toc131461462)

[Figure S10: Calibration curve – Model type: Random forest; Dataset: Administrative database; Timepoint: Discharge; Predictors: Systematic review predictors 37](#_Toc131461463)

[Table S17: Variable importance factors – Model type: Random forest; Dataset: Administrative database; Timepoint: Discharge; Predictors: Systematic review predictors 37](#_Toc131461464)

[Results of previously developed models from prior literature (Table S18, Figures S11-S12, Table S19, Figure S13, Table S20, Figures S14-S15, Table S21, Figure S16) 38](#_Toc131461465)

[Table S18: LACE+ Variable availability 38](#_Toc131461466)

[Figure S11: ROC curve for LACE+ 39](#_Toc131461467)

[Figure S12: Precision-recall (PPV-sensitivity) curve for LACE+ 40](#_Toc131461468)

[Table S19: Performance metrics for LACE+ 40](#_Toc131461469)

[Figure S13: Calibration curve for LACE+ 42](#_Toc131461470)

[Table S20: Ali 2019 Variable availability 42](#_Toc131461471)

[Figure S14: ROC curve for Ali 2019 44](#_Toc131461472)

[Figure S15: Precision-recall (PPV-sensitivity) curve for Ali 2019 45](#_Toc131461473)

[Table S21: Performance metrics for Ali 2019 45](#_Toc131461474)

[Figure S16: Calibration curve for Ali 2019 47](#_Toc131461475)

[Performance of Random Forest model trained on all predictors model (Table S22, Figures S17-S19, Table S23, Figure S20, Table S24) 48](#_Toc131461476)

[Table S22: Model specifications and AUC ROC performance for random forest model trained on all available predictors 48](#_Toc131461477)

[Figure S17: Training set ROC curve for random forest model trained on all available predictors 49](#_Toc131461478)

[Figure S18: Test set ROC curve for random forest model trained on all available predictors 50](#_Toc131461479)

[Figure S19: Precision-recall (PPV-sensitivity) curve for random forest model trained on all available predictors 51](#_Toc131461480)

[Table S23: Performance metrics for random forest model trained on all available predictors 51](#_Toc131461481)

[Figure S20: Calibration curve for random forest model trained on all available predictors 53](#_Toc131461482)

[Table S24: Variable importance factors for random forest model trained on all available predictors 54](#_Toc131461483)

[Performance of random forest model trained on k1-k25 highest importance variables (Table S25) 55](#_Toc131461484)

[Performance of random forest model trained on k12 highest importance variables – (Figures S21-S23, Table S26, Figure S24) 64](#_Toc131461485)

[Figure S21: Training set ROC curve for k25 random forest model 64](#_Toc131461486)

[Figure S22: Test set ROC curve for k12 random forest model 65](#_Toc131461487)

[Figure S23: Precision-recall (PPV-sensitivity) curve for k12 random forest model 66](#_Toc131461488)

[Table S26: Performance metrics for k12 random forest model 66](#_Toc131461489)

[Figure S24: Calibration curve for k12 random forest model 68](#_Toc131461490)

[Performance of k12 Random Forest model using only Initial consultation variables (Table S27, Figures S25-S27, Table S28, Figure S28) 69](#_Toc131461491)

[Table S27: Predictors and model specification for k12 model using only Initial consultation variables 69](#_Toc131461492)

[Figure S25: Training set ROC curve for k12 random forest model using only Initial consultation variables 70](#_Toc131461493)

[Figure S26: Test set ROC curve for k12 random forest model using only Initial consultation variables 71](#_Toc131461494)

[Figure S27: Precision-recall (PPV-sensitivity) curve for k12 random forest model using only Initial consultation variables 72](#_Toc131461495)

[Table S28: Performance metrics for k12 random forest model using only Initial consultation variables 72](#_Toc131461496)

[Figure S28: Calibration curve for k12 random forest model using only Initial consultation variables 74](#_Toc131461497)

[Performance of Logistic Regression model trained on all predictors (Table S29, Figures S29-S31, Table S30, Figure S32, Table S31) 75](#_Toc131461498)

[Table S29: Model specifications and AUC ROC performance for logistic regression model trained on all available predictors 75](#_Toc131461499)

[Figure S29: Training set ROC curve for logistic regression model trained on all available predictors 76](#_Toc131461500)

[Figure S30: Test set ROC curve for logistic regression model trained on all available predictors 77](#_Toc131461501)

[Figure S31: Precision-recall (PPV-sensitivity) curve for random forest model trained on all available predictors 78](#_Toc131461502)

[Table S30: Performance metrics for logistic regression model trained on all available predictors 78](#_Toc131461503)

[Figure S32: Calibration curve for logistic regression model trained on all available predictors 80](#_Toc131461504)

[Table S31: Coefficients for logistic regression model trained on all available predictors 80](#_Toc131461505)

[Performance of Logistic Regression model trained on all predictors available at Initial consultation (Table S32, Figures S33-S35, Table S33, Figure S36, Table S34) 82](#_Toc131461506)

[Table S32: Model specifications and AUC ROC performance for logistic regression model trained on all predictors available at Initial consultation 82](#_Toc131461507)

[Figure S33: Training set ROC curve for logistic regression model trained on all predictors available at Initial consultation 84](#_Toc131461508)

[Figure S34: Test set ROC curve for logistic regression model trained on all predictors available at Initial consultation 85](#_Toc131461509)

[Figure S37: Precision-recall (PPV-sensitivity) curve for random forest model trained on all predictors available at Initial consultation 86](#_Toc131461510)

[Table S33: Performance metrics for logistic regression model trained on all predictors available at Initial consultation 86](#_Toc131461511)

[Figure S36: Calibration curve for logistic regression model trained on all predictors available at Initial consultation 88](#_Toc131461512)

[Table S34: Coefficients for logistic regression model trained on all available predictors 88](#_Toc131461513)

[Predictor summary statistics SMART-only dataset (Table S35) 90](#_Toc131461514)

[Missingness per variable - SMART-only dataset (Table S36) 91](#_Toc131461515)

[SMART-only dataset cohort creation flow diagram – Figure S37 92](#_Toc131461516)

[Random Forest model performance - SMART discharge model (Table S37, Figures S38-S40, Table S38, Figure S41, Table S39) 93](#_Toc131461517)

[Table S37: Variables, model specifications, and AUC for SMART-only model 93](#_Toc131461518)

[Figure S38: Training set ROC curve for SMART-only random forest discharge model 94](#_Toc131461519)

[Figure S39: Test set ROC curve for SMART-only random forest discharge model 95](#_Toc131461520)

[Figure S40: Precision-recall (PPV-sensitivity) curve for SMART-only random forest discharge model 96](#_Toc131461521)

[Table S38: Performance metrics for SMART-only random forest discharge model 97](#_Toc131461522)

[Figure S41: Calibration curve for SMART-only random forest discharge model 98](#_Toc131461523)

[Table S39: Variable importance factors for SMART-only random forest discharge model 99](#_Toc131461524)

[Random Forest model performance - SMART Initial consultation model (Table S40, Figures S42-S44, Table S41, Figure S45, Table S42) 100](#_Toc131461525)

[Table S40: Variables, model specifications, and AUC for SMART-only random forest Initial consultation model 100](#_Toc131461526)

[Figure S42: Training set ROC curve for SMART-only random forest Initial consultation model 101](#_Toc131461527)

[Figure S43: Test set ROC curve for SMART-only random forest Initial consultation model 102](#_Toc131461528)

[Figure S44: Precision-recall (PPV-sensitivity) curve for SMART-only random forest Initial consultation model 103](#_Toc131461529)

[Table S41: Performance metrics for SMART-only random forest Initial consultation model 103](#_Toc131461530)

[Figure S45: Calibration curve for SMART-only random forest Initial consultation model 105](#_Toc131461531)

[Table S42: Variable importance factors for SMART-only random forest Initial consultation model 106](#_Toc131461532)

[Cause-specific models 106](#_Toc131461533)

[Table S43: Outcome definitions and predictors for each outcome from the literature 106](#_Toc131461534)

[Table S44: Causes of readmission in this study cohort 110](#_Toc131461535)

[Table S45: Variable generation – outcomes 110](#_Toc131461536)

[Variable generation – predictors 122](#_Toc131461537)

[Baseline characteristics 129](#_Toc131461538)

[Table S60: Cause-specific models developed 138](#_Toc131461539)

[Full model evaluation 141](#_Toc131461540)

[References 190](#_Toc131461541)

## Variable availability – Table S1

| **Variable** | **Availability in SMART** | **Availability in administrative database** |
| --- | --- | --- |
| **Majority high-importance vote in Delphi survey** | | |
| CHF | Yes | No |
| Liver disease | Yes | No |
| *Dementia | Yes | No |
| Charlson Comorbidity Index ≥1 | Yes | No |
| Substance abuse | Yes | No |
| Dependent functional status | No | No |
| *Preoperative patient-reported pain level | Yes | No |
| Increasing number of previous admissions (number of prior admissions (administrative databaset 12 months)) | No | Yes |
| In-hospital complication (any) | Yes | No |
| *ICU/HDU admission | Yes | Yes |
| *Return to theatre | Yes | Yes |
| **Majority moderate-importance vote in Delphi survey** | | |
| Preoperative patient-reported level of function | Yes | No |
| **Peripheral vascular disease | Yes | No |
| **Diabetes | Yes | No |
| **Coagulopathy | Yes | No |
| **BMI | Yes | No |
| **Age | Yes | Yes |
| Preoperative patient-reported quality of life | No | No |
| Paralysis | No | No |
| **Length of stay | Yes | Yes |
| **CKD | Yes | No |
| **Arrhythmia | Yes | No |
| Transfusion during surgery | Yes | No |
| Smoking | Yes | No |
| Pulmonary disease | Yes | No |
| Duration of operation | Yes | Yes |
| **Low SES | Yes | Yes |
| Wound class | No | Yes |
| **Depression | Yes | No |
| Previous stroke | Yes | No |
| Rehab stay | No | No |
| **Anaemia | Yes | No |
| **History of cancer | Yes | No |
| **Majority high-importance vote in focus group** | | |
| High risk of infection: High risk of infectiond state, active IVDU, infection in other primary joint replacement | Yes | No |
| Inadequate pain management at discharge | No | No |
| Surgical factors: prolonged/complex/difficult surgery, surgical misadventure | Yes | Yes |
| Pain catastrophizing; analgesia intolerance/catastrophic pain | No | No |
| Threshold for readmission – e.g. the specific ED, or whether there is a junior registrar reviewing the patient | No | No |
| Transplant recipient (better at self-managing medications, better screened for other comorbidities, etc.) | No | No |
| **Majority moderate-importance vote in focus group** | | |
| Poor access to post-op care: lives far from hospital, lack of access to allied health support, lack of access to telehealth support | Yes | Yes |
| Patient-related biopsychosocial: lower education level, poor health literacy, non-English speaking | Yes (used ‘Interpreter required’ as proxy variable) | No |
| Resilience | No | No |
| Personality disorders | No (poorly captured so did not use it) | No |
| Poor skin condition | No | No |
| **Systematic review variables** | | |
| **Number of prior ED presentations (12 months) | No | Yes |
| **Sex | Yes | Yes |
| **Hypertension | Yes | No |
| **Historical knee procedures | No | Yes |
| **Ethnicity | Yes (used ‘Interpreter required’ as proxy for ethnicity) | No |
| CHF = congestive heart failure; ICU/HDU = intensive care unit/high dependency unit; BMI = body mass index; CKD = chronic kidney disease; SES = socioeconomic status; IVDU = intravenous drug use; emergency department = ED; * = voted as high-importance despite lack of systematic review evidence; ** = not voted as high-importance despite systematic review evidence available indicating this is a risk factor which correlates with readmission | | |

## Variable generation – Table S2

| **Variable** | **Variable generation process** |
| --- | --- |
| CHF | Variable exists in Registry |
| Liver disease | Variable exists in Registry |
| Dementia | Variable exists in Registry |
| Charlson Comorbidity Index | Variable exists in Registry |
| Substance abuse | Combined two Registry variables:   - Alcohol Abuse - Drug addiction |
| Preoperative patient-reported pain level | Variable exists in Registry. Used VR-12 Pain Interference |
| Increasing number of previous admissions (number of prior admissions (administrative databaset 12 months)) | Variable exists in administrative database |
| In-hospital complication (any) | Indicator variable for the occurrence of any complication captured in the Registry. Refer to the Registry Cohort Profile supplementary file for the full list of complications captured. |
| ICU/HDU admission | Variable exists in administrative database, and Registry |
| Return to theatre | Variable exists in administrative database, and Registry |
| Preoperative patient-reported level of function | Variable exists in Registry. Used VR-12 mental score and VR-12 physical score |
| Peripheral vascular disease | Variable exists in Registry |
| Diabetes | Variable exists in Registry |
| Coagulopathy | Variable exists in Registry. Used the comorbidity variable ‘thrombocytopenia’ |
| BMI | Variable exists in Registry |
| Age | Variable exists in administrative database, and Registry |
| Length of stay | Variable exists in administrative database, and Registry |
| CKD | Variable exists in Registry |
| Arrhythmia | Variable exists in Registry |
| Smoking | Variable (current smoker) exists in Registry |
| Pulmonary disease | Variable exists in Registry |
| Surgical factors: prolonged/complex/difficult surgery, surgical misadventure | Variable exists in administrative database, and Registry. Used duration of operation (minutes) as a proxy for this |
| Low SES | Used SEIFA score in Registry, and Pensioner Card Holder in administrative database. |
| Wound class | Variable exists in administrative database. |
| Depression | Variable exists in SMART |
| Previous stroke | Variable exists in SMART |
| Anaemia | Variable exists in SMART |
| History of cancer | Indicator variable for the occurrence of any of the following:   - Ovarian cancer - Uterine cancer - Thyroid cancer - Lung cancer - Endometrial cancer - Cervical cancer - Bladder cancer - Renal cancer - Melanoma - Prostate cancer - Bowel cancer - Breast cancer - Solid tumour (Charlson comorbidity) - Leukaemia (Charlson comorbidity) - Lymphoma (Charlson comorbidity) - Liposarcoma - Oesophageal cancer - Osteosarcoma - Cancer of the vocal cords - Hepatic cancer - Squamous cell carcinoma - Stomach cancer - Soft tissue cancer - Testicular cancer - Throat cancer |
| High risk of infection: High risk of infectiond state, active IVDU, infection in other primary joint replacement | Indicator variable for the occurrence of any variable available in the Registry which could increase risk of infection (either through the comorbidity itself, its treatment, and/or its sequelae):   - Rheumatoid arthritis - Connective tissue disorder - Ankylosing spondylitis - Autoimmune hepatitis - Crohn’s disease - Juvenile arthritis - Psoriasis - Psoriatic arthritis - Sarcoidosis - Scleroderma - Sjogren’s syndrome - Systemic lupus erythematosus - Temporal arteritis - Ulcerative colitis - Vasculitis - History of cancer (see previous variable) - Diabetes - AIDS - Neutropenia - Multiple myeloma - Osteomyelitis - Septic arthritis - Splenectomy - Smoker - Prosthetic joint infection |
| Poor access to post-op care: lives far from hospital, lack of access to allied health support, lack of access to telehealth support | In administrative database and SMART, used ASGC remoteness code. |
| Patient-related biopsychosocial: lower education level, poor health literacy, non-English speaking | Used Interpreter required from SMART as a proxy variable. |
| Number of prior ED presentations (12 months) | Variable exists in administrative database |
| Sex | Variable exists in Registry and administrative database |
| Hypertension | Variable exists in Registry |
| Historical knee procedures | Variable exists in administrative database |
| Transfusion during surgery | Variable exists in SMART |
| Ethnicity | Variable exists in Registry (I used Interpreter required as a proxy variable) |
| CHF = congestive heart failure; ICU/HDU = intensive care unit/high dependency unit; BMI = body mass index; CKD = chronic kidney disease; SES = socioeconomic status; SEIFA = Socioeconomic Indexes for Areas; IVDU = intravenous drug use; emergency department = ED; SMART = St Vincent’s Melbourne Arthroplasty Outcomes Registry; VR-12 = Veteran’s RAND 12-item Health survey | |

## Variable preparation – Table S3

| **Variable** | **Type of variable** | **Pre-processing** |
| --- | --- | --- |
| CHF | Binary indicator (dummy) | None |
| Liver disease | Dummy | Collapsed two categories of severity into ‘present’ (vs ‘absent’) |
| Dementia | Dummy | None |
| Charlson Comorbidity Index | Categorical | Collapsed into three categories:  Zero  One  ≥Two |
| Substance abuse | Dummy | None |
| Preoperative patient-reported pain level | Continuous | Normalised (centred and scaled) |
| Increasing number of previous admissions (number of prior admissions (administrative databaset 12 months)) | Categorical | Collapsed into four categories:  Zero  One  Two  ≥Three |
| In-hospital complication (any) | Dummy | None |
| ICU/HDU admission | Categorical | Collapsed into four categories for number of days in ICU:  Zero  One  ≥Two |
| Return to theatre | Dummy | None |
| Preoperative patient-reported level of function | Continuous | Normalised (centred and scaled) |
| Peripheral vascular disease | Dummy | None |
| Diabetes | Categorical | None |
| Coagulopathy | Dummy | None |
| BMI | Continuous | Normalised (centred and scaled) |
| Age | Continuous | Normalised (centred and scaled) |
| Length of stay | Continuous | None |
| CKD | Dummy | None |
| Arrhythmia | Dummy | None |
| Smoking | Dummy | None |
| Pulmonary disease | Dummy | None |
| Surgical factors: prolonged/complex/difficult surgery, surgical misadventure (duration of operation – minutes) | Continuous | Normalised (centred and scaled) |
| Low SES | Dummy (pensioner card status)  Categorical (SEIFA) | None |
| Wound class | Dummy | Collapsed into two categories:  Clean  Not clean |
| Depression | Dummy | None |
| Previous stroke | Dummy | None |
| Anaemia | Dummy | None |
| History of cancer | Dummy | None |
| High risk of infection: High risk of infectiond state, active IVDU, infection in other primary joint replacement | Dummy | None |
| Poor access to post-op care: lives far from hospital, lack of access to allied health support, lack of access to telehealth support | Categorical | Collapsed into three categories:  Inner regional Australia  Major cities of Australia  Outer regional or remote Australia |
| Patient-related biopsychosocial: lower education level, poor health literacy, non-English speaking | Dummy | None |
| Number of prior ED presentations (12 months) | Categorical | Collapsed into three categories:  Zero  One  ≥Two |
| Sex | Dummy | None |
| Hypertension | Dummy | None |
| Historical knee procedures | Categorical | Collapsed into four categories:  Zero  One  Two  ≥Three |
| Transfusion during surgery | Categorical | Collapsed into four categories:  Zero  One  Two  ≥Three |
| Ethnicity | Dummy | None |
| CHF = congestive heart failure; ICU/HDU = intensive care unit/high dependency unit; BMI = body mass index; CKD = chronic kidney disease; SES = socioeconomic status; SEIFA = Socioeconomic Indexes for Areas; IVDU = intravenous drug use; emergency department = ED; All numeric (continuous) variables were normalised using z-standardisation prior to training models. | | |

## Models – Table S4

| **Model specifications** | **Predictor selection** | **Predictors** |
| --- | --- | --- |
| Model type: Logistic regression  Dataset: administrative database  Timepoint: Initial consultation | High-importance in Delphi and focus group | Increasing number of previous admissions |
| Model type: Logistic regression  Dataset: administrative database  Timepoint: Initial consultation | High-importance and moderate-importance in Delphi and focus group | Increasing number of previous admissions; Age; Low SES; Poor access to post-op care: lives far from hospital, lack of access to allied health support, lack of access to telehealth support |
| Model type: Logistic regression  Dataset: administrative database  Timepoint: Initial consultation | Systematic review predictors | Number of prior ED presentations (12 months); Increasing number of previous admissions; Age; Sex; Low SES; Historical knee procedures |
| Model type: Random forest  Dataset: administrative database  Timepoint: Initial consultation | High-importance in Delphi and focus group | Increasing number of previous admissions |
| Model type: Random forest  Dataset: administrative database  Timepoint: Initial consultation | High-importance and moderate-importance in Delphi and focus group | Increasing number of previous admissions; Age; Low SES; Poor access to post-op care: lives far from hospital, lack of access to allied health support, lack of access to telehealth support |
| Model type: Random forest  Dataset: administrative database  Timepoint: Initial consultation | Systematic review predictors | Number of prior ED presentations (12 months); Increasing number of previous admissions; Age; Sex; Low SES; Historical knee procedures |
| Model type: Logistic regression  Dataset: administrative database  Timepoint: Discharge | High-importance in Delphi and focus group | Increasing number of previous admissions; ICU/HDU admission; Return to theatre; Duration of operation/Surgical factors: prolonged/complex/difficult surgery, surgical misadventure |
| Model type: Logistic regression  Dataset: administrative database  Timepoint: Discharge | High-importance and moderate-importance in Delphi and focus group | Increasing number of previous admissions; ICU/HDU admission; Return to theatre; Age; Length of stay; Duration of operation/Surgical factors: prolonged/complex/difficult surgery, surgical misadventure; Low SES; Poor access to post-op care: lives far from hospital, lack of access to allied health support, lack of access to telehealth support |
| Model type: Logistic regression  Dataset: administrative database  Timepoint: Discharge | Systematic review predictors | Length of stay; Number of prior ED presentations (12 months); Increasing number of previous admissions; Age; Sex; Low SES; Historical knee procedures |
| Model type: Random forest  Dataset: administrative database  Timepoint: Discharge | High-importance in Delphi and focus group | Increasing number of previous admissions; ICU/HDU admission; Return to theatre; Duration of operation/Surgical factors: prolonged/complex/difficult surgery, surgical misadventure |
| Model type: Random forest  Dataset: administrative database  Timepoint: Discharge | High-importance and moderate-importance in Delphi and focus group | Increasing number of previous admissions; ICU/HDU admission; Return to theatre; Age; Length of stay; Duration of operation/Surgical factors: prolonged/complex/difficult surgery, surgical misadventure; Low SES; Poor access to post-op care: lives far from hospital, lack of access to allied health support, lack of access to telehealth support |
| Model type: Random forest  Dataset: administrative database  Timepoint: Discharge | Systematic review predictors | Length of stay; Number of prior ED presentations (12 months); Increasing number of previous admissions; Age; Sex; Low SES; Historical knee procedures |
| Model type: Logistic regression  Dataset: Combined  Timepoint: Initial consultation | High-importance in Delphi and focus group | CHF; Liver disease; Dementia; Charlson Comorbidity Index; Substance abuse; Increasing number of previous admissions; High risk of infection |
| Model type: Logistic regression  Dataset: Combined  Timepoint: Initial consultation | High-importance and moderate-importance in Delphi and focus group  LASSO | CHF; Liver disease; Dementia; Charlson Comorbidity Index; Substance abuse; Increasing number of previous admissions; Peripheral vascular disease; Diabetes; Coagulopathy; BMI; Age; CKD; Arrhythmias; Smoking; Pulmonary disease; Low SES; Depression; Previous stroke; Anaemia; History of cancer; High risk of infection; Poor access to post-op care: lives far from hospital, lack of access to allied health support, lack of access to telehealth support; Patient-related biopsychosocial: lower education level, poor health literacy, non-English speaking |
| Model type: Logistic regression  Dataset: Combined  Timepoint: Initial consultation | Systematic review predictors  LASSO | Number of prior ED presentations (12 months); Increasing number of previous admissions; Age; Sex; Charlson Comorbidity Index; Low SES; Patient-related biopsychosocial: lower education level, poor health literacy, non-English speaking; Historical knee procedures; Depression; Diabetes; History of cancer; Hypertension; CKD; Anaemia; Coagulopathy; Liver disease; BMI; Substance abuse; CHF; Arrhythmias; Peripheral vascular disease |
| Model type: Random forest  Dataset: Combined  Timepoint: Initial consultation | High-importance in Delphi and focus group | CHF; Liver disease; Dementia; Charlson Comorbidity Index; Substance abuse; Increasing number of previous admissions; High risk of infection |
| Model type: Random forest  Dataset: Combined  Timepoint: Initial consultation | High-importance and moderate-importance in Delphi and focus group | CHF; Liver disease; Dementia; Charlson Comorbidity Index; Substance abuse; Increasing number of previous admissions; Peripheral vascular disease; Diabetes; Thrombocytopenia; BMI; Age; CKD; Arrhythmias; Smoking; Pulmonary disease; Low SES; Depression; Previous stroke; Anaemia; History of cancer; High risk of infection; Poor access to post-op care: lives far from hospital, lack of access to allied health support, lack of access to telehealth support; Patient-related biopsychosocial: lower education level, poor health literacy, non-English speaking |
| Model type: Random forest  Dataset: Combined  Timepoint: Initial consultation | Systematic review predictors | Number of prior ED presentations (12 months); Increasing number of previous admissions; Age; Sex; Low SES; Historical knee procedures |
| Model type: Logistic regression  Dataset: Combined  Timepoint: Discharge | High-importance in Delphi and focus group | Preoperative patient-reported pain level; CHF; Liver disease; Dementia; Charlson Comorbidity Index; Substance abuse; Increasing number of previous admissions; In-hospital complication (any); ICU/HDU admission; Return to theatre; High risk of infection; Duration of operation/Surgical factors: prolonged/complex/difficult surgery, surgical misadventure |
| Model type: Logistic regression  Dataset: Combined  Timepoint: Discharge | High-importance and moderate-importance in Delphi and focus group  LASSO | CHF; Liver disease; Dementia; Charlson Comorbidity Index; Substance abuse; Increasing number of previous admissions; In-hospital complication (any); ICU/HDU admission; Return to theatre; Preoperative patient-reported level of function; Preoperative patient-reported pain level; Peripheral vascular disease; Diabetes; Coagulopathy; BMI; Age; Length of stay; CKD; Arrhythmias; Smoking; Pulmonary disease; Duration of operation/Surgical factors: prolonged/complex/difficult surgery, surgical misadventure; Low SES; Depression; Previous stroke; Anaemia; History of cancer; High risk of infection; Patient-related biopsychosocial: lower education level, poor health literacy, non-English speaking; Poor access to post-op care: lives far from hospital, lack of access to allied health support, lack of access to telehealth support; Transfusion during surgery |
| Model type: Logistic regression  Dataset: Combined  Timepoint: Discharge | Systematic review predictors  LASSO | Length of stay; Number of prior ED presentations (12 months);  Increasing number of previous admissions; Age; Sex; Charlson Comorbidity Index; Low SES; Patient-related biopsychosocial: lower education level, poor health literacy, non-English speaking; In-hospital complication (any); Historical knee procedures; Depression; Diabetes; History of cancer; Hypertension; CKD; Anaemia; Coagulopathy; Liver disease; BMI; Substance abuse; CHF; Arrhythmias; Peripheral vascular disease |
| Model type: Random forest  Dataset: Combined  Timepoint: Discharge | High-importance in Delphi and focus group | Preoperative patient-reported pain level; CHF; Liver disease; Dementia; Charlson Comorbidity Index; Substance abuse; Increasing number of previous admissions; In-hospital complication (any); ICU/HDU admission; Return to theatre; High risk of infection; Duration of operation/Surgical factors: prolonged/complex/difficult surgery, surgical misadventure |
| Model type: Random forest  Dataset: Combined  Timepoint: Discharge | High-importance and moderate-importance in Delphi and focus group | Congestive heart failure (CHF); Liver disease; Dementia; Charlson Comorbidity Index; Substance abuse; Increasing number of previous admissions; In-hospital complication (any); ICU/HDU admission; Return to theatre; Preoperative patient-reported level of function; Preoperative patient-reported pain level; Peripheral vascular disease; Diabetes; Coagulopathy; BMI; Age; Length of stay; CKD; Arrhythmias; Smoking; Pulmonary disease; Duration of operation/Surgical factors: prolonged/complex/difficult surgery, surgical misadventure; Low SES; Depression; Previous stroke; Anaemia; History of cancer; High risk of infection; Patient-related biopsychosocial: lower education level, poor health literacy, non-English speaking; Poor access to post-op care: lives far from hospital, lack of access to allied health support, lack of access to telehealth support; Transfusion during surgery |
| Model type: Random forest  Dataset: Combined  Timepoint: Discharge | Systematic review predictors | Length of stay; Number of prior ED presentations (12 months);  Increasing number of previous admissions; Age; Sex; Charlson Comorbidity Index; Low SES; Patient-related biopsychosocial: lower education level, poor health literacy, non-English speaking; In-hospital complication (any); Historical knee procedures; Depression; Diabetes; History of cancer; Hypertension; CKD; Anaemia; Coagulopathy; Liver disease; BMI; Substance abuse; CHF; Arrhythmias; Peripheral vascular disease |
| CHF = congestive heart failure; ICU/HDU = intensive care unit/high dependency unit; BMI = body mass index; CKD = chronic kidney disease; SES = socioeconomic status; IVDU = intravenous drug use; emergency department = ED; LASSO = Least Absolute Shrinkage and Selection Operator | | |

## Missingness per variable – Table S5

| **Variable** | **Missingness in SMART** | **Missingness in ADMINISTRATIVE DATABASE** |
| --- | --- | --- |
| **Majority high-importance vote in Delphi survey** | | |
| CHF | 0 | N/A |
| Liver disease | 0 | N/A |
| Dementia | 0 | N/A |
| Charlson Comorbidity Index ≥1 | 0 | N/A |
| Substance abuse | 0 | N/A |
| Dependent functional status | N/A | N/A |
| Preoperative patient-reported pain level | 530 (14.383%) | N/A |
| Increasing number of previous admissions | N/A | 0 |
| In-hospital complication (any) | 0 | N/A |
| ICU/HDU admission | N/A (administrative database variable was used) | 0 |
| Return to theatre | N/A (administrative database variable was used) | 0 |
| **Majority moderate-importance vote in Delphi survey** | | |
| Preoperative patient-reported level of function | VR-12 mental score = 525 (14.247%)  VR-12 physical score = 525 (14.247%) | N/A |
| Peripheral vascular disease | 0 | N/A |
| Diabetes | 0 | N/A |
| Coagulopathy | 0 | N/A |
| BMI | 1 (0.027%) | N/A |
| Age | 0 | 0 |
| Preoperative patient-reported quality of life | N/A | N/A |
| Paralysis | N/A | N/A |
| Length of stay | 0 | 0 |
| CKD | 0 | N/A |
| Arrhythmia | 0 | N/A |
| Transfusion during surgery | 0 | N/A |
| Smoking | 0 | N/A |
| Pulmonary disease | 0 | N/A |
| Duration of operation | 0 | 0 |
| Low socioeconomic status (SES) | 0 | Pensioner card = 0 |
| Wound class | N/A | 0 |
| Depression | 0 | N/A |
| Previous stroke | 0 | N/A |
| Anaemia | 0 | N/A |
| History of cancer | 0 | N/A |
| **Majority high-importance vote in focus group** | | |
| High risk of infection: High risk of infectiond state, active IVDU, infection in other primary joint replacement | 0 | N/A |
| Inadequate pain management at discharge | N/A | N/A |
| Surgical factors: prolonged/complex/difficult surgery, surgical misadventure | 0 | 0 |
| Pain catastrophizing; analgesia intolerance/catastrophic pain | N/A | N/A |
| Threshold for readmission - e.g. the specific ED, or whether there is a junior registrar reviewing the patient | N/A | N/A |
| Transplant recipient (better at self-managing medications, better screened for other comorbidities, etc.) | 0 | N/A |
| **Majority moderate-importance vote in focus group** | | |
| Poor access to post-op care: lives far from hospital, lack of access to allied health support, lack of access to telehealth support | 0 | 10 (0.271%) |
| Poor understanding of disease and post-op course | N/A | N/A |
| Patient-related biopsychosocial: lower education level, poor health literacy, non-English speaking | 36 (0.633%) | N/A |
| Resilience | N/A | N/A |
| **Systematic review predictors** | | |
| Number of prior ED presentations (12 months, 6 months) | N/A | 0 |
| Sex | 0 | 0 |
| Hypertension | 0 | N/A |
| Historical knee procedures | N/A | 0 |
| Ethnicity | 36 (0.633%) | N/A |
| CHF = congestive heart failure; ICU/HDU = intensive care unit/high dependency unit; BMI = body mass index; CKD = chronic kidney disease; SES = socioeconomic status; SEIFA = Socioeconomic Indexes for Areas; IVDU = intravenous drug use; emergency department = ED; SMART = St Vincent’s Melbourne Arthroplasty Outcomes Registry | | |

## Statistical Packages and Version Numbers – Table S6

| **Package** | **Version number (reference)** |
| --- | --- |
| tidyverse | version 1.3.1 (Wickham H, Averick M, Bryan J, Chang W, McGowan LD, François R, Grolemund G, Hayes A, Henry L, Hester J, Kuhn M, Pedersen TL, Miller E, Bache SM, Müller K, Ooms J, Robinson D, Seidel DP, Spinu V, Takahashi K, Vaughan D, Wilke C, Woo K, Yutani H (2019). “Welcome to the tidyverse.” Journal of Open Source Software, 4(43), 1686. doi:10.21105/joss.01686.) |
| tidymodels | version 1.0.0 (Kuhn M, Wickham H (2020). Tidymodels: a collection of packages for modeling and machine learning using tidyverse principles.. https://www.tidymodels.org.) |
| ranger | version 0.13.1 (Wright MN, Ziegler A (2017). “ranger: A Fast Implementation of Random Forests for High Dimensional Data in C++ and R.” Journal of Statistical Software, 77(1), 1–17. doi:10.18637/jss.v077.i01.) |
| glmnet | version 4.1.3 (Friedman J, Hastie T, Tibshirani R (2010). “Regularization Paths for Generalized Linear Models via Coordinate Descent.” Journal of Statistical Software, 33(1), 1–22. doi: 10.18637/jss.v033.i01, https://www.jstatsoft.org/v33/i01/.) |
| naniar | version 0.6.1 (Tierney NJ, Cook DH. Expanding tidy data principles to facilitate missing data exploration, visualization and assessment of imputations. arXiv preprint arXiv:1809.02264. 2018 Sep 7.) |
| haven | version 2.4.3 (https://haven.tidyverse.org, https://github.com/tidyverse/haven |
| pROC | version 1.17.0.1 (Robin X, Turck N, Hainard A, Tiberti N, Lisacek F, Sanchez J, Müller M (2011). “pROC: an open-source package for R and S+ to analyze and compare ROC curves.” BMC Bioinformatics, 12, 77.) |
| CalibrationCurves | version 0.1.2 (De Cock B, Nieboer D, Van Calster B, Steyerberg E, Vergouwe Y (2016). “The CalibrationCurves package: validating predicted probabilities against binary events.” https://github.com/BavoDC/CalibrationCurves.) |
| cutpointr | 1.1.2 (Thiele C, Hirschfeld G (2021). “cutpointr: Improved Estimation and Validation of Optimal Cutpoints in R.” Journal of Statistical Software, 98(11), 1–27. doi: 10.18637/jss.v098.i11.) |

## Training set performance of all models developed in primary analysis – Table S7

| Model specifications | Predictor selection | Training set AUC ROC |
| --- | --- | --- |
| Model type: Logistic regression  Dataset: administrative database  Timepoint: Initial consultation | High-importance in Delphi and focus group | 0.499 |
| Model type: Logistic regression  Dataset: administrative database  Timepoint: Initial consultation | High-importance and moderate-importance in Delphi and focus group | 0.554 |
| Model type: Logistic regression  Dataset: administrative database  Timepoint: Initial consultation | Systematic review predictors | 0.563 = best administrative database initial consultation model |
| Model type: Random forest  Dataset: administrative database  Timepoint: Initial consultation | High-importance in Delphi and focus group | 0.498 |
| Model type: Random forest  Dataset: administrative database  Timepoint: Initial consultation | High-importance and moderate-importance in Delphi and focus group | 0.532 |
| Model type: Random forest  Dataset: administrative database  Timepoint: Initial consultation | Systematic review predictors | 0.551 |
| Model type: Logistic regression  Dataset: administrative database  Timepoint: Discharge | High-importance in Delphi and focus group | 0.518 |
| Model type: Logistic regression  Dataset: administrative database  Timepoint: Discharge | High-importance and moderate-importance in Delphi and focus group | 0.579 |
| Model type: Logistic regression  Dataset: administrative database  Timepoint: Discharge | Systematic review predictors | 0.622 |
| Model type: Random forest  Dataset: administrative database  Timepoint: Discharge | High-importance in Delphi and focus group | 0.514 |
| Model type: Random forest  Dataset: administrative database  Timepoint: Discharge | High-importance and moderate-importance in Delphi and focus group | 0.607 |
| Model type: Random forest  Dataset: administrative database  Timepoint: Discharge | Systematic review predictors | 0.657 = best administrative database discharge model |
| Model type: Logistic regression  Dataset: Combined  Timepoint: Initial consultation | High-importance in Delphi and focus group | 0.551 |
| Model type: Logistic regression  Dataset: Combined  Timepoint: Initial consultation | High-importance and moderate-importance in Delphi and focus group | 0.553 |
| Model type: Logistic regression  Dataset: Combined  Timepoint: Initial consultation | Systematic review predictors | 0.602 |
| Model type: Random forest  Dataset: Combined  Timepoint: Initial consultation | High-importance in Delphi and focus group | 0.529 |
| Model type: Random forest  Dataset: Combined  Timepoint: Initial consultation | High-importance and moderate-importance in Delphi and focus group | 0.584 |
| Model type: Random forest  Dataset: Combined  Timepoint: Initial consultation | Systematic review predictors | 0.638* |
| Model type: Logistic regression  Dataset: Combined  Timepoint: Discharge | High-importance in Delphi and focus group | 0.591 |
| Model type: Logistic regression  Dataset: Combined  Timepoint: Discharge | High-importance and moderate-importance in Delphi and focus group | 0.621 |
| Model type: Logistic regression  Dataset: Combined  Timepoint: Discharge | Systematic review predictors | 0.681 |
| Model type: Random forest  Dataset: Combined  Timepoint: Discharge | High-importance in Delphi and focus group | 0.545 |
| Model type: Random forest  Dataset: Combined  Timepoint: Discharge | High-importance and moderate-importance in Delphi and focus group | 0.649 |
| Model type: Random forest  Dataset: Combined  Timepoint: Discharge | Systematic review predictors | 0.701** |
| *highest training set AUC ROC for Initial consultation; **highest training set AUC ROC for discharge | | |

## Comparison of baseline demographics and readmission rate for variables with ≥10% missingness - missing vs non-missing (Tables S8-S10)

### Table S8: Comparison of patients missing vs not missing VR-12 mental score

| **Feature** | **Missing** | **Non-missing** | **P-value** |
| --- | --- | --- | --- |
| Age (mean (SD)) | 71.436 (7.966) | 69.257 (8.939) | <0.001 |
| Sex (% female) | 61.905% | 63.924% | 0.4 |
| BMI (mean (SD)) | 31.671 (6.484) | 33.380 (6.540) | <0.001 |
| Charlson Comorbidity Index | One = 164 (31.238%)  ≥Two = 93 (17.714%) | One = 862 (27.278%)  ≥Two = 673 (21.297%) | 0.070 |
| Readmissions | 28 (5.333%) | 223 (7.057%) | 0.174 |
| BMI = body mass index; SD = standard deviation | | | |

### Table S9: Comparison of patients missing vs not missing VR-12 physical score

| **Feature** | **Missing** | **Non-missing** | **P-value** |
| --- | --- | --- | --- |
| Age (mean (SD)) | 71.436 (7.966) | 69.257 (8.939) | <0.001 |
| Sex (% female) | 61.905% | 63.924% | 0.4 |
| BMI (mean (SD)) | 31.671 (6.484) | 33.380 (6.540) | <0.001 |
| Charlson Comorbidity Index | One = 164 (31.238%)  ≥Two = 93 (17.714%) | One = 862 (27.278%)  ≥Two = 673 (21.297%) | 0.070 |
| Readmissions | 28 (5.333%) | 223 (7.057%) | 0.174 |
| BMI = body mass index; SD = standard deviation | | | |

### Table S10: Comparison of patients missing vs not missing VR-12 pain score

| **Feature** | **Missing** | **Non-missing** | **P-value** |
| --- | --- | --- | --- |
| Age (mean (SD)) | 71.387 (7.954) | 69.261 (8.944) | <0.001 |
| Sex (% female) | 61.887% | 63.930% | 0.392 |
| BMI (mean (SD)) | 31.734 (6.515) | 33.372 (6.538) | <0.001 |
| Charlson Comorbidity Index | One = 164 (30.943%)  ≥Two = 95 (17.925%) | One = 862 (27.322%)  ≥Two = 671 (21.268%) | 0.101 |
| Readmissions | 28 (5.283%) | 223 (7.068%) | 0.157 |
| BMI = body mass index; SD = standard deviation | | | |

## Main models variable importance factors (Tables S11-S12)

### Table S11: Model type: Random forest; Dataset: Combined; Timepoint: Initial consultation

| **Variable** | **Importance (descending order)** |
| --- | --- |
| BMI | 55.359 |
| Age | 33.141 |
| Historical knee procedures | 20.637 |
| SEIFA | 20.465 |
| Charlson Comorbidity Index | 6.892 |
| Hypertension | 4.937 |
| Depression | 4.785 |
| Sex | 4.571 |
| interpreter | 4.372 |
| Cancer (any) | 4.389 |
| Diabetes | 4.335 |
| Admissions in the past 12 months | 3.659 |
| Anaemia | 3.040 |
| Emergency presentations in the past 12 months | 2.834 |
| Chronic kidney disease | 2.679 |
| Congestive heart failure | 2.495 |
| Peripheral vascular disease | 2.431 |
| Liver disease | 1.671 |
| BMI = body mass index; SEIFA = Socioeconomic Indexes for Areas | |

### Table S12: Model type: Random forest; Dataset: Combined; Timepoint: Discharge

| **Variable** | **Importance (descending order)** |
| --- | --- |
| BMI | 47.753 |
| Length of stay | 42.339 |
| Age | 30.672 |
| Historical knee procedures | 18.542 |
| SEIFA | 19.114 |
| Charlson Comorbidity Index | 6.737 |
| Pre-discharge complication | 5.551 |
| Sex | 4.857 |
| Hypertension | 4.455 |
| Depression | 4.300 |
| interpreter | 4.348 |
| Cancer (any) | 4.280 |
| Diabetes | 3.988 |
| Admissions in the past 12 months | 3.569 |
| Emergency presentations in the past 12 months | 2.952 |
| Chronic kidney disease | 2.836 |
| Anaemia | 2.573 |
| Congestive heart failure | 2.320 |
| BMI = body mass index; SEIFA = Socioeconomic Indexes for Areas | |

## Main models training set receiver operating characteristic (ROC) curves (Figures S1-S2)

### Figure S1: Model type: Random forest; Dataset: Combined; Timepoint: Initial consultation


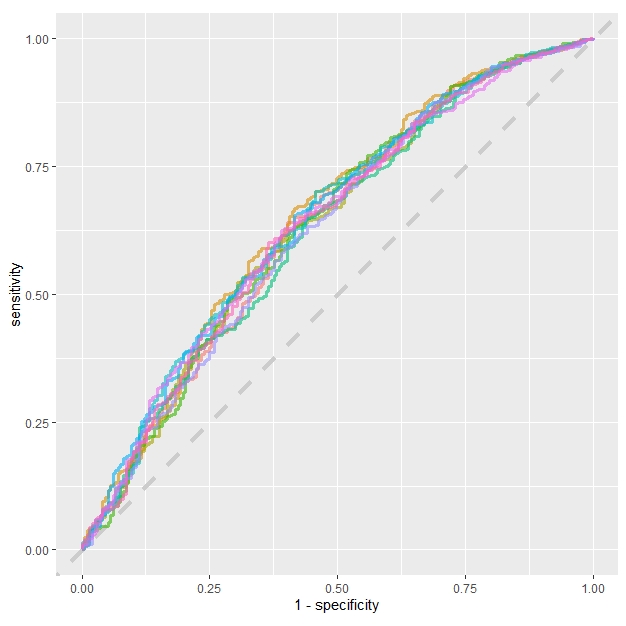


### Figure S2: Model type: Random forest; Dataset: Combined; Timepoint: Discharge


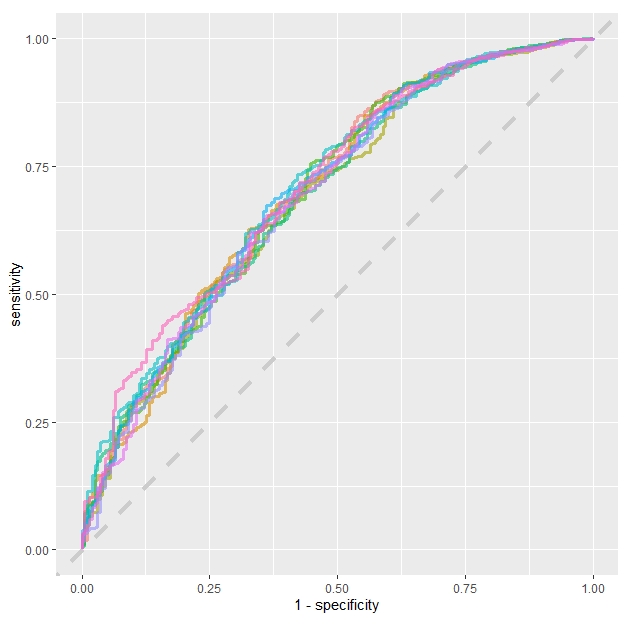


## Main models precision-recall (PPV-sensitivity) curves (Figures S3-S4)

### Figure S3: Model type: Random forest; Dataset: Combined; Timepoint: Initial consultation


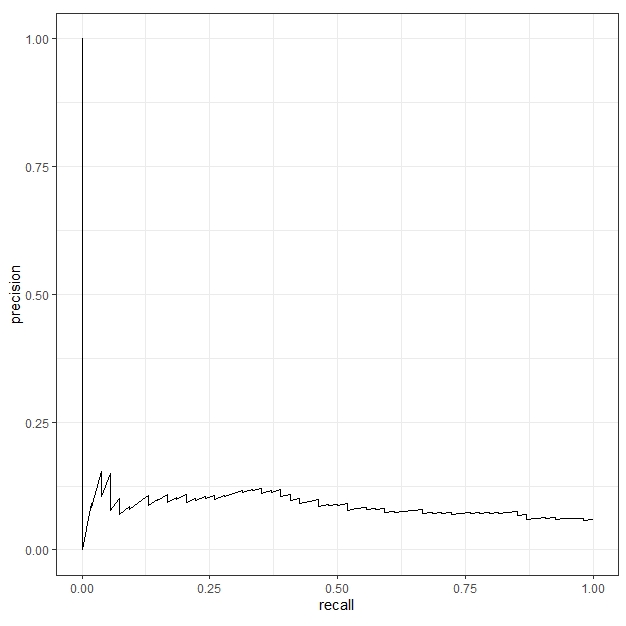


### Figure S4: Model type: Random forest; Dataset: Combined; Timepoint: Discharge


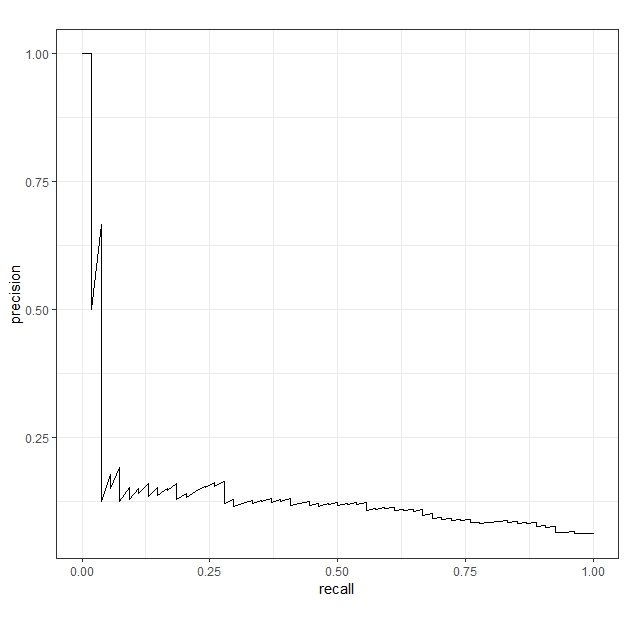


### Table S13: Main models performance metrics – Initial consultation and discharge (Model type: Random forest; Dataset: Combined)

| Probability threshold | Metrics |
| --- | --- |
| Optimal threshold to maximise MCC (Initial consultation random forest model) = 0.116 | True positives = 21  True negatives = 710  False negatives = 33  False positives = 157  MCC = 0.124  Sensitivity (recall) = 0.389  Specificity = 0.819  Youden index = 0.208  F statistic = 0.181  PPV (precision) = 0.118  NPV = 0.956 |
| Threshold-free metrics (Initial consultation model) | Discrimination:  AUC ROC (test set) = 0.617 (0.538 to 0.696)  AUC PR (AUC PPV-sensitivity) = 0.086  Calibration:  Slope = 0.407 (0.123 to 0.691)  Intercept = -0.324 (-0.607 to -0.040)  ICI = 0.031  E50 = 0.017  E90 = 0.056  Emax = 0.461 |
| Optimal threshold to maximise MCC (Discharge random forest model) = 0.093 | True positives = 30  True negatives = 650  False negatives = 24  False positives = 217  MCC = 0.162  Sensitivity (recall) = 0.556  Specificity = 0.750  Youden index = 0.305  F statistic = 0.199  PPV (precision) = 0.121  NPV = 0.964 |
| Threshold-free metrics (discharge model) | Discrimination:  AUC ROC (test set) = 0.692 (0.621 to 0.764)  AUC PR (AUC PPV-sensitivity) = 0.137  Calibration:  Slope = 0.625 (0.364 to 0.885)  Intercept = -0.312 (-0.599 to -0.026)  ICI = 0.019  E50 = 0.008  E90 = 0.044  Emax = 0.167 |
| MCC = Matthews Correlation Coefficient; PPV = positive predictive value; NPV = negative predictive value; AUC ROC = Area under the receiver operating characteristic curve; AUC PR = Area under the precision-recall (PPV-sensitivity) curve; ICI = Integrated calibration index; E50 = median of the absolute difference between observed and predicted probabilities; E90 = 90th percentile of the absolute difference between observed and predicted probabilities; Emax = maximal absolute difference between observed and predicted probabilities of the outcome | |

#### Selection of Probability Threshold for Model Evaluation

There is no recognised probability threshold for readmission in this population. Therefore, we selected the threshold at which Matthews Correlation Coefficient (MCC) was maximised. This metric is useful for the evaluation of models developed on imbalanced datasets as it gives a realistic measure of model performance [1], which is relevant for readmission following TKA because incidence is less than 10% [2, 3].

## Best administrative database models – full performance evaluation (Figures S5-S6, Tables S14-S15, Figures S7-S9, Table S16, Figure S10, Table S17)

### Figure S5: Training set ROC curve – Model type: Logistic regression; Dataset: Administrative database; Timepoint: Initial consultation; Predictors: Systematic review predictors


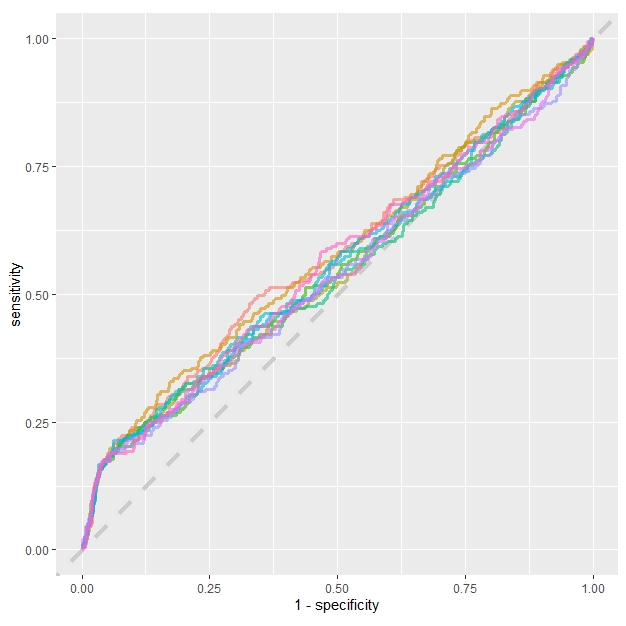


### Figure S6: Precision-recall (PPV-sensitivity) curve – Model type: Logistic regression; Dataset: Administrative database; Timepoint: Initial consultation; Predictors: Systematic review predictors


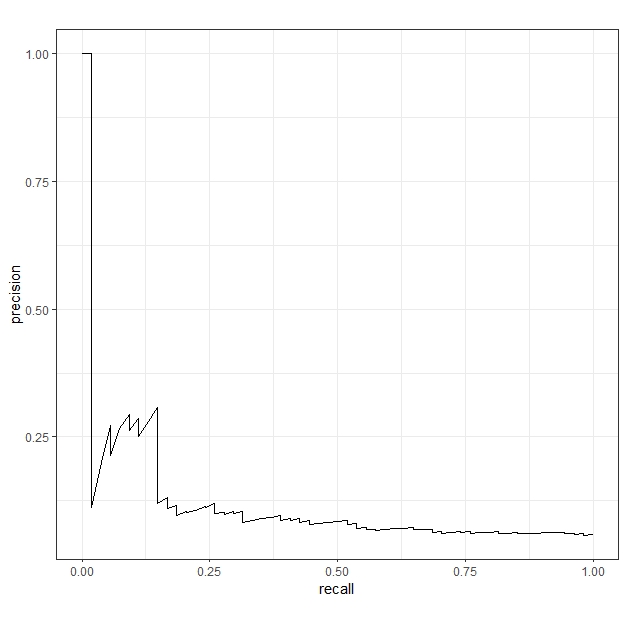


### Table S14: Performance metrics

| Threshold | Metrics |
| --- | --- |
| Optimal threshold to maximise MCC = 0.263 | True positives = 8  True negatives = 849  False negatives = 46  False positives = 18  MCC = 0.181  Sensitivity (recall) = 0.148  Specificity = 0.979  Youden index = 0.127  F statistic = 0.200  PPV (precision) = 0.308  NPV = 0.949 |
| Threshold-free metrics | Discrimination:  AUC ROC (test set) = 0.589 (0.506 to 0.673)  AUC PR = 0.118  Calibration:  Slope = 0.765 (0.327 to 1.202)  Intercept = -0.221 (-0.501 to 0.058)  ICI = 0.012  E50 = 0.008  E90 = 0.016  Emax = 0.195 |
| MCC = Matthews Correlation Coefficient; PPV = positive predictive value; NPV = negative predictive value; AUC ROC = Area under the receiver operating characteristic curve; AUC PR = Area under the precision-recall (PPV-sensitivity) curve; ICI = Integrated calibration index; E50 = median of the absolute difference between observed and predicted probabilities; E90 = 90th percentile of the absolute difference between observed and predicted probabilities; Emax = maximal absolute difference between observed and predicted probabilities of the outcome | |

### Table S15: Coefficients – Model type: Logistic regression; Dataset: Administrative database; Timepoint: Initial consultation; Predictors: Systematic review predictors

| **Variable** | **Coefficient** |
| --- | --- |
| (Intercept) | -2.663 |
| Age | -0.031 |
| Emergency presentations in the past 12 months (one) | 0.186 |
| Emergency presentations in the past 12 months (two or more) | 0.084 |
| Admissions in the past 12 months (one) | -0.062 |
| Admissions in the past 12 months (two) | -0.082 |
| Admissions in the past 12 months (three or more) | -0.031 |
| Sex (male) | 0.043 |
| Pensioner Concession Card | 0.119 |
| Historical knee procedures (one) | -0.058 |
| Historical knee procedures (two) | 0.026 |
| Historical knee procedures (three or more) | 0.339 |

### Figure S7: Training set ROC curve – Model type: Random forest; Dataset: Administrative database; Timepoint: Discharge; Predictors: Systematic review predictors


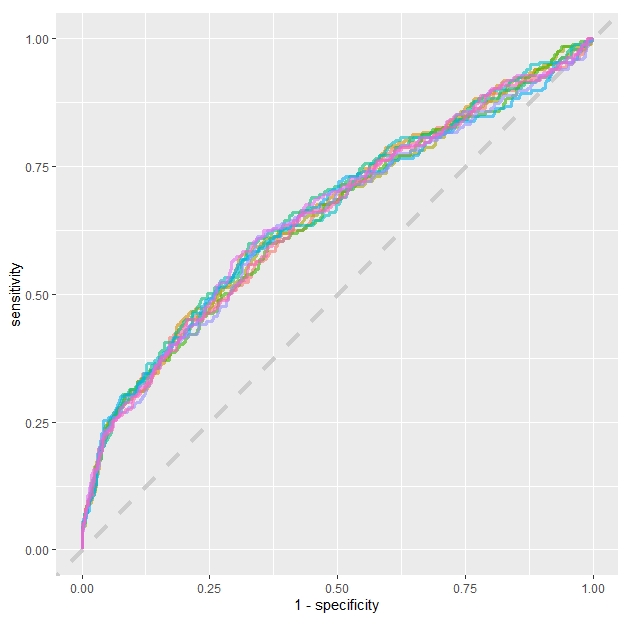


### Figure S8: Test set ROC curve – Model type: Random forest; Dataset: Administrative database; Timepoint: Discharge; Predictors: Systematic review predictors


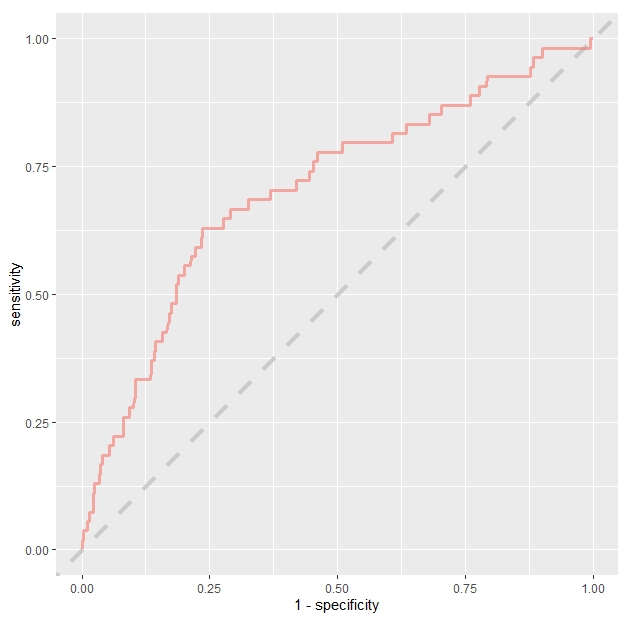


### Figure S9: Precision-recall (PPV-sensitivity) curve – Model type: Random forest; Dataset: Administrative database; Timepoint: Discharge; Predictors: Systematic review predictors


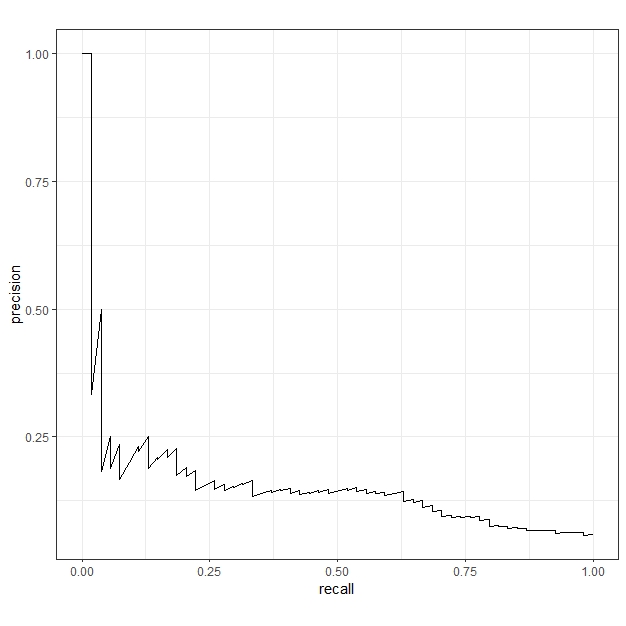


### Table S16: Performance metrics – Model type: Random forest; Dataset: Administrative database; Timepoint: Discharge; Predictors: Systematic review predictors

| **Thresholds** | **Metrics** |
| --- | --- |
| Optimal threshold to maximise MCC = 0.072 | True positives = 34  True negatives = 663  False negatives = 20  False positives = 204  MCC = 0.212  Sensitivity (recall) = 0.629  Specificity = 0.765  Youden index = 0.394  F statistic = 0.233  PPV (precision) = 0.143  NPV = 0.971 |
| Threshold-free metrics | Discrimination:  AUC ROC (test set) = 0.705 (0.628 to 0.782)  AUC PR = 0.155  Calibration:  Slope = 0.870 (0.563 to 1.178)  Intercept = -0.163 (-0.447 to 0.121)  ICI = 0.016  E50 = 0.012  E90 = 0.029  Emax = 0.125 |
| MCC = Matthews Correlation Coefficient; PPV = positive predictive value; NPV = negative predictive value; AUC ROC = Area under the receiver operating characteristic curve; AUC PR = Area under the precision-recall (PPV-sensitivity) curve; ICI = Integrated calibration index; E50 = median of the absolute difference between observed and predicted probabilities; E90 = 90th percentile of the absolute difference between observed and predicted probabilities; Emax = maximal absolute difference between observed and predicted probabilities of the outcome | |

### Figure S10: Calibration curve – Model type: Random forest; Dataset: Administrative database; Timepoint: Discharge; Predictors: Systematic review predictors


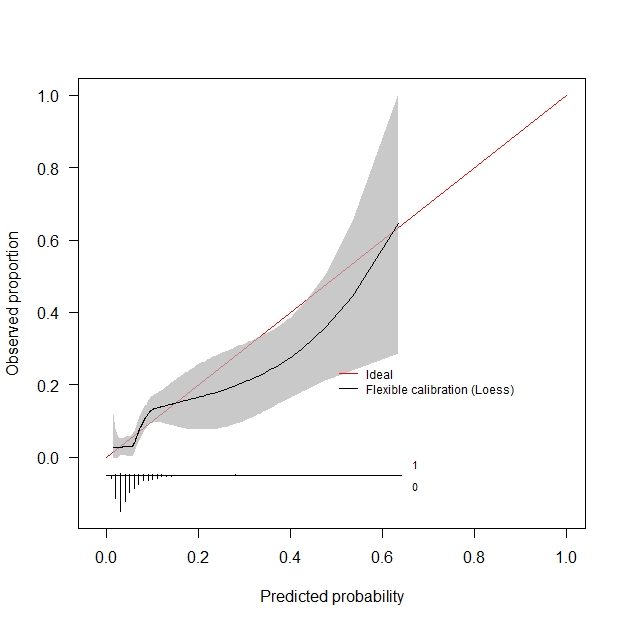


### Table S17: Variable importance factors – Model type: Random forest; Dataset: Administrative database; Timepoint: Discharge; Predictors: Systematic review predictors

| **Variable** | **Variable importance factor** |
| --- | --- |
| Length of stay | 41.938 |
| Age | 29.024 |
| Historical knee procedures | 16.458 |
| Sex | 4.194 |
| Admissions in the past 12 months | 3.340 |
| Pensioner Concession Card | 3.250 |
| Emergency presentations in the past 12 months | 2.906 |

## Results of previously developed models from prior literature (Table S18, Figures S11-S12, Table S19, Figure S13, Table S20, Figures S14-S15, Table S21, Figure S16)

### Table S18: LACE+ Variable availability

| **Variable in LACE+** | **Available in study dataset** |
| --- | --- |
| Sex | Yes |
| Urgency of admission | No (high proportion of missing data) |
| Discharge institution – teaching vs small | No (single-institution dataset) |
| Discharge institution – large vs small | No (single-institution dataset) |
| Age | Yes |
| Length of stay | Yes |
| Charlson Comorbidity Index | Yes |
| Number of ED visits in the past six months | Yes |
| Number of urgent admissions previous year | Yes |
| Number of elective admissions previous year | Yes |
| CMG score | No (however, value of 0 was used as the LACE+ model developers found this made little impact on model performance) |
| Days on alternative level of care status | Yes (used ‘days in ICU’) |
| CMG = (Canadian) Case Mix Group; ICU = intensive care unit | |

### Figure S11: ROC curve for LACE+


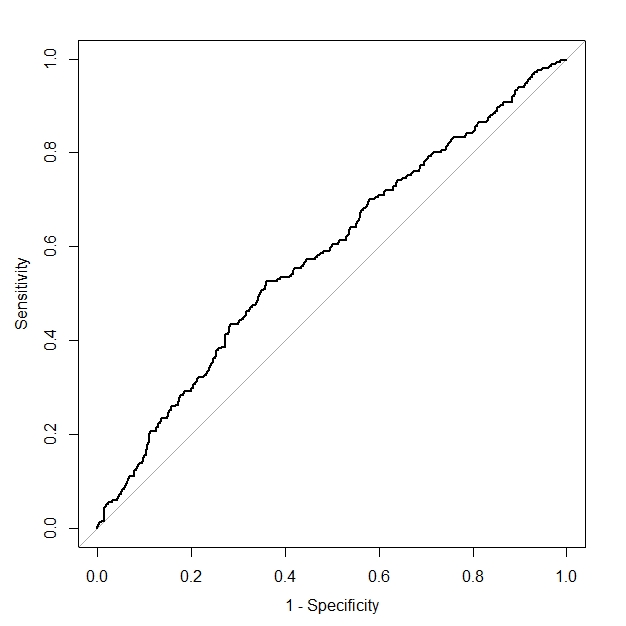


### Figure S12: Precision-recall (PPV-sensitivity) curve for LACE+


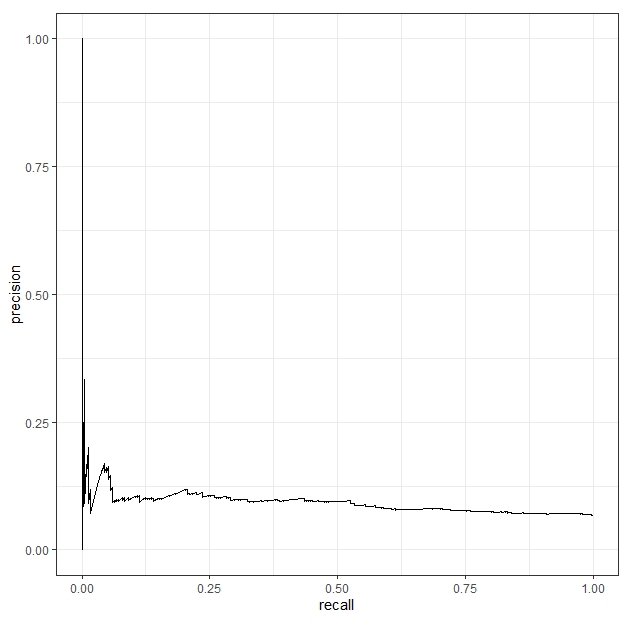


### Table S19: Performance metrics for LACE+

| **Threshold** | **Performance metrics** |
| --- | --- |
| Optimal threshold to maximise MCC = 0.732 | True positives = 132  True negatives = 2198  False negatives = 119  False positives = 1236  MCC = 0.087  Sensitivity (recall) = 0.526  Specificity = 0.640  Youden index = 0.166  F statistic = 0.163  PPV (precision) = 0.097  NPV = 0.949 |
| Threshold-free metrics | Discrimination:  AUC ROC = 0.583 (0.545 to 0.620)  AUC PR = 0.091  Calibration:  Slope = 0.659 (0.370 to 0.947)  Intercept = -3.618 (-3.747 to -3.489)  ICI = 0.642  E50 = 0.649  E90 = 0.711  Emax = 0.846 |
| MCC = Matthews Correlation Coefficient; PPV = positive predictive value; NPV = negative predictive value; AUC ROC = Area under the receiver operating characteristic curve; AUC PR = Area under the precision-recall (PPV-sensitivity) curve; ICI = Integrated calibration index; E50 = median of the absolute difference between observed and predicted probabilities; E90 = 90th percentile of the absolute difference between observed and predicted probabilities; Emax = maximal absolute difference between observed and predicted probabilities of the outcome | |

### Figure S13: Calibration curve for LACE+


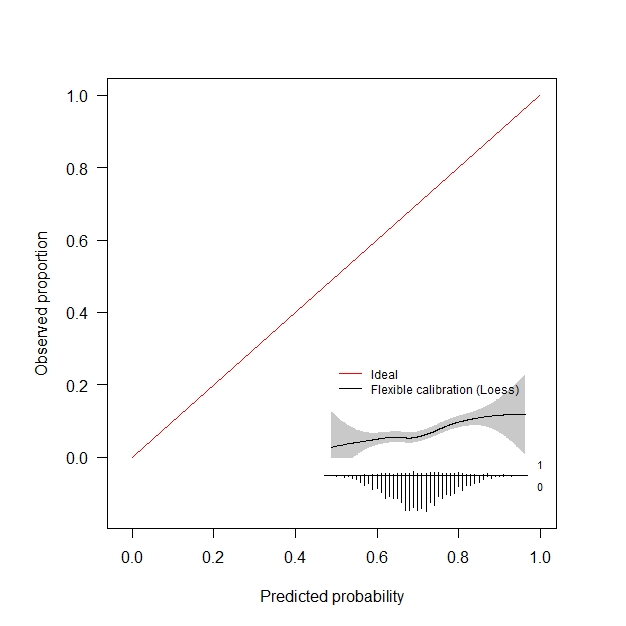


### Table S20: Ali 2019 Variable availability

| **Variable in Ali** | **Variable in combined dataset** |
| --- | --- |
| Age | Yes |
| Sex | Yes |
| Patellofemoral replacement | Not included because this study is on primary TKA |
| Partial (UKA) | Not included because this study is on primary TKA |
| SES | Yes |
| Year of operation | Not included because the model developed in this study is intended for individual patient prediction and there are also years of operation outside the range in Ali 2019 |
| Emergency visits | Yes |
| Ethnicity/race | No |
| Return to theatre | Yes |
| Length of stay | Yes |
| Diabetes mellitus | Yes |
| Hypertension | Yes |
| Arrhythmias | Yes |
| Valvular disease | No |
| Congestive heart failure | Yes |
| Peripheral vascular disease | Yes |
| Chronic lung disease | Yes |
| Lung circulation disorders | No |
| Cancer with metastases | No |
| Renal disease | Yes |
| Dementia (new onset) | Yes (however, not specifically ‘new onset’) |
| Psychoses | Yes |
| Alcohol abuse | Yes |
| Drug abuse | Yes |
| Depression | Yes |
| Other mental health disorder | Yes |
| Living alone | Yes |
| Liver disease | Yes |
| Peptic ulcer | Yes |
| Paraplegia | No |
| Blood loss anaemia | No |
| Deficiency anaemia | Yes (used ‘anaemia’) |
| Coagulopathy | Yes |
| Weight loss | No |
| Fluid and electrolyte disorders | No |
| Hypothyroidism | Yes |
| Obesity | Yes |
| Other neuro diseases | Yes |
| Rheumatic disorders | Yes |
| Other autoimmune disorders | Yes |
| Previous pneumonia | No |
| Previous stroke | Yes |
| Previous AMI | Yes |
| Cancer without metastases | Yes |
| Rheumatoid arthritis aetiology | Yes |
| Traumatic aetiology | Yes |
| Other diagnosis aetiology | Yes |
| UKA = unicompartmental knee arthroplasty; SES = socioeconomic status; TKA = total knee arthroplasty; AMI = acute myocardial infarction | |

### Figure S14: ROC curve for Ali 2019


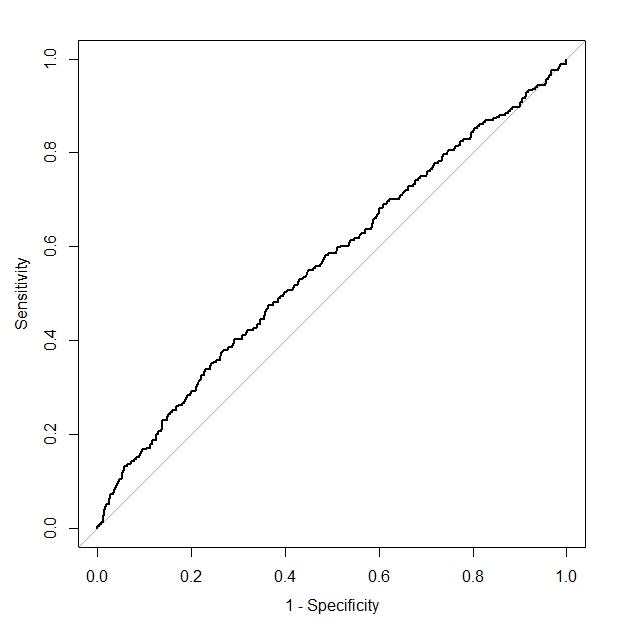


### Figure S15: Precision-recall (PPV-sensitivity) curve for Ali 2019


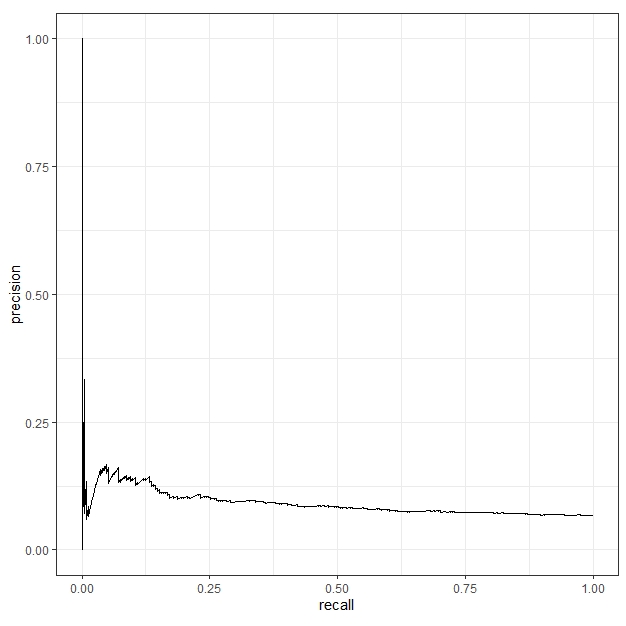


### Table S21: Performance metrics for Ali 2019

| **Threshold** | **Performance metrics** |
| --- | --- |
| Optimal threshold to maximise MCC = 0.247 | True positives = 33  True negatives = 3236  False negatives = 218  False positives = 197  MCC = 0.077  Sensitivity (recall) = 0.131  Specificity = 0.943  Youden index = 0.074  F statistic = 0.137  PPV (precision) = 0.143  NPV = 0.937 |
| Threshold-free metrics | Discrimination:  AUC ROC = 0.563 (0.525 to 0.602)  AUC PR = 0.090  Calibration:  Slope = 0.672 (0.300 to 1.044)  Intercept = -1.022 (-1.151 to -0.894)  ICI = 0.100  E50 = 0.100  E90 = 0.139  Emax = 0.522 |
| MCC = Matthews Correlation Coefficient; PPV = positive predictive value; NPV = negative predictive value; AUC ROC = Area under the receiver operating characteristic curve; AUC PR = Area under the precision-recall (PPV-sensitivity) curve; ICI = Integrated calibration index; E50 = median of the absolute difference between observed and predicted probabilities; E90 = 90th percentile of the absolute difference between observed and predicted probabilities; Emax = maximal absolute difference between observed and predicted probabilities of the outcome | |

### Figure S16: Calibration curve for Ali 2019


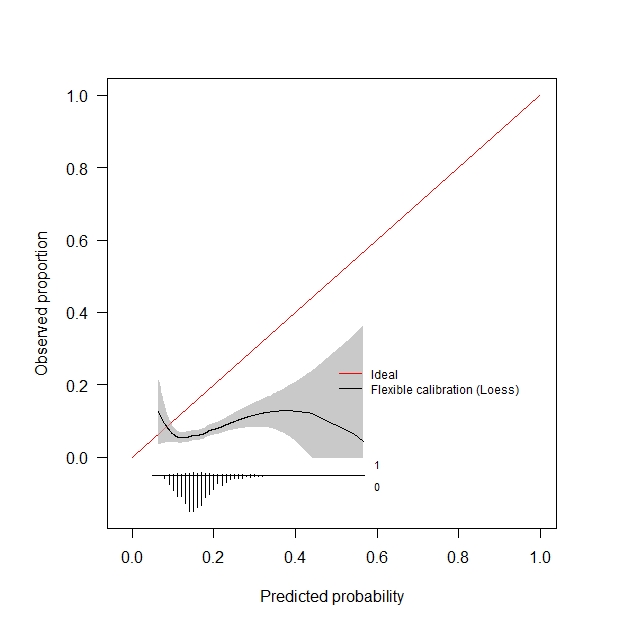


## Performance of Random Forest model trained on all predictors model (Table S22, Figures S17-S19, Table S23, Figure S20, Table S24)

### Table S22: Model specifications and AUC ROC performance for random forest model trained on all available predictors

| **Model** | **Predictors** | **Model specifications** | **AUC** |
| --- | --- | --- | --- |
| All predictors (i.e. systematic review variables, as well as all high- and moderate-importance variables through Delphi and focus group) | Congestive heart failure; Liver disease; Dementia; Charlson Comorbidity Index = 1; Substance abuse; Preoperative pain level; Admissions in the last 12 months; Pre-discharge complication (any type of complication); Days in ICU; Return to theatre prior to discharge; Preoperative mental function; Preoperative physical function; Peripheral vascular disease; Diabetes; Thrombocytopenia (proxy for coagulopathy); BMI; Age; Length of stay; Chronic kidney disease; Arrhythmia; Smoking; Pulmonary disease; Duration of operation (minutes); Low SES; Wound class; Depression; Previous stroke; Anaemia; History of cancer; High risk of infection; Remoteness area (major cities of Australia) – proxy for poor access to post-op care; Interpreter required; Emergency presentations in the last 12 months; Number of prior knee procedures; Male sex; Hypertension; Transfusion during surgery | Random forest  5000 trees | Train = 0.689  Test = 0.678 (95% confidence interval: 0.601 to 0.755) |
| ICU = intensive care unit; BMI = body mass index; SES = socioeconomic status | | | |

### Figure S17: Training set ROC curve for random forest model trained on all available predictors


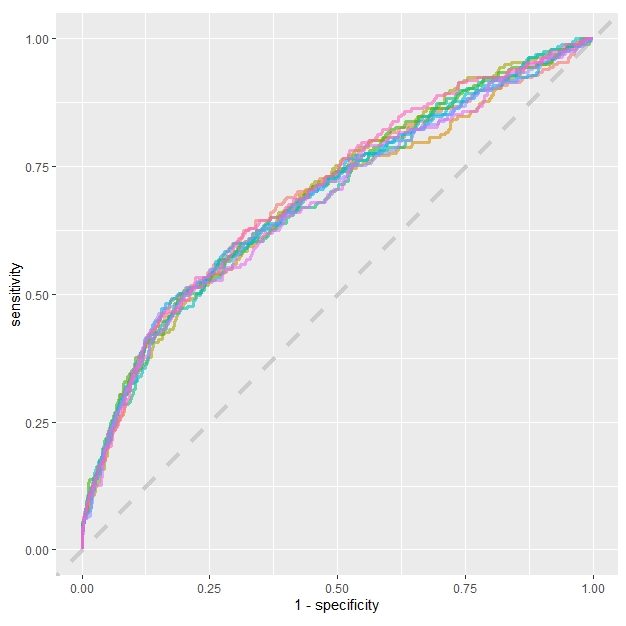


### Figure S18: Test set ROC curve for random forest model trained on all available predictors


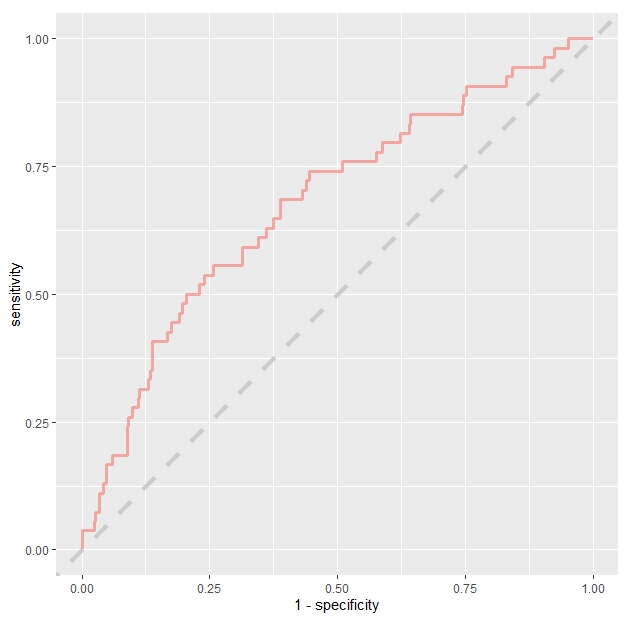


### Figure S19: Precision-recall (PPV-sensitivity) curve for random forest model trained on all available predictors


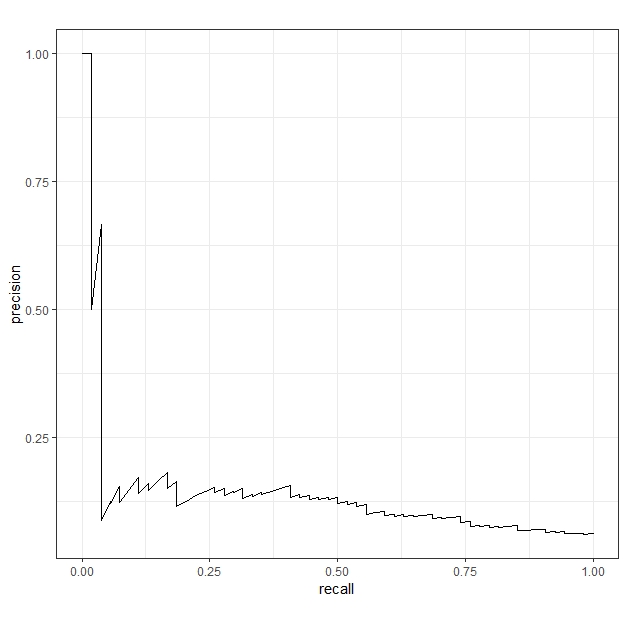


### Table S23: Performance metrics for random forest model trained on all available predictors

| **Thresholds** | **Performance metrics** |
| --- | --- |
| Optimal threshold to maximise MCC = 0.144 | True positives = 22  True negatives = 748  False negatives = 32  False positives = 119  MCC = 0.176  Sensitivity (recall) = 0.407  Specificity = 0.863  Youden index = 0.270  F statistic = 0.226  PPV (precision) = 0.156  NPV = 0.959 |
| Threshold-free metrics | Discrimination:  AUC PR = 0.137  Calibration:  Slope = 0.662 (0.373 to 0.950)  Intercept = -0.335 (-0.618 to -0.051)  ICI = 0.023  E50 = 0.014  E90 = 0.050  Emax = 0.113 |
| MCC = Matthews Correlation Coefficient; PPV = positive predictive value; NPV = negative predictive value; AUC ROC = Area under the receiver operating characteristic curve; AUC PR = Area under the precision-recall (PPV-sensitivity) curve; ICI = Integrated calibration index; E50 = median of the absolute difference between observed and predicted probabilities; E90 = 90th percentile of the absolute difference between observed and predicted probabilities; Emax = maximal absolute difference between observed and predicted probabilities of the outcome | |

### Figure S20: Calibration curve for random forest model trained on all available predictors


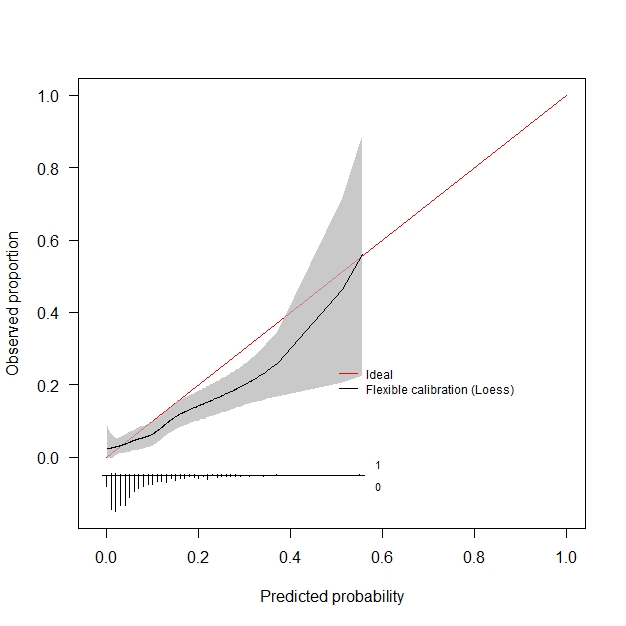


### Table S24: Variable importance factors for random forest model trained on all available predictors

| **Variable** | **Importance (descending order)** |
| --- | --- |
| Length of stay | 30.559 |
| BMI | 27.209 |
| VR-12 mental score | 24.789 |
| VR-12 physical score | 21.409 |
| Duration of operation (minutes) | 20.203 |
| Age | 17.637 |
| Historical knee procedures | 13.624 |
| SEIFA | 11.469 |
| VR-12 pain score | 6.265 |
| Transfusion during surgery | 6.004 |
| Pre-discharge complication | 4.211 |
| Charlson Comorbidity Index | 4.136 |
| Remoteness area | 3.220 |
| Hypertension | 2.716 |
| Sex | 2.667 |
| Pensioner concession card | 2.664 |
| Cancer (any) | 2.601 |
| Time in ICU (days) | 2.570 |
| Admissions in the past 12 months | 2.521 |
| interpreter | 2.497 |
| Diabetes | 2.447 |
| COPD | 2.394 |
| Depression | 2.350 |
| High risk of infection | 2.196 |
| Emergency presentations in the past 12 months | 2.179 |
| Chronic kidney disease | 2.172 |
| Anaemia | 1.952 |
| Stroke | 1.882 |
| Peripheral vascular disease | 1.547 |
| Congestive heart failure | 1.487 |
| Smoker | 1.483 |
| Return to theatre | 1.175 |
| Liver disease | 0.927 |
| Substance abuse | 0.652 |
| Dementia | 0.015 |
| Coagulopathy | 0.009 |
| Arrhythmia | 0.007 |
| Wound condition | 0.002 |
| VR-12 = Veteran’s RAND 12-item Health Survey; SEIFA = Socioeconomic Indexes for Areas; ICU = intensive care unit; HDU = High dependency unit; ED = emergency department; CHF = congestive heart failure; CKD = chronic kidney disease | |

## Performance of random forest model trained on k1-k25 highest importance variables (Table S25)

| **Predictors** | **Specifications** | **AUC** |
| --- | --- | --- |
| **k = 1**  Length of stay | 5000 trees  Used mean imputation instead of k-nearest neighbours imputation | Train = 0.595 |
| **k = 2**  Length of stay  BMI | 5000 trees  Used mean imputation instead of k-nearest neighbours imputation | Train = 0.595 |
| **k = 3**  Length of stay  BMI  VR-12 mental score | 5000 trees | Train = 0.579 |
| **k = 4**  Length of stay  BMI  VR-12 mental score  VR-12 physical score | 5000 trees | Train = 0.588 |
| **k = 5**  Length of stay  BMI  VR-12 mental score  VR-12 physical score  Duration of operation (minutes) | 5000 trees | Train = 0.596 |
| **k = 6**  Length of stay  BMI  VR-12 mental score  VR-12 physical score  Duration of operation (minutes)  Age | 5000 trees | Train = 0.606 |
| **k = 7**  Length of stay  BMI  VR-12 mental score  VR-12 physical score  Duration of operation (minutes)  Age  Historical knee procedures | 5000 trees | Train = 0.661 |
| **k = 8**  Length of stay  BMI  VR-12 mental score  VR-12 physical score  Duration of operation (minutes)  Age  Historical knee procedures  SEIFA | 5000 trees | Train = 0.674 |
| **k = 9**  Length of stay  BMI  VR-12 mental score  VR-12 physical score  Duration of operation (minutes)  Age  Historical knee procedures  SEIFA  VR-12 pain score | 5000 trees | Train = 0.674 |
| **k = 10**  Length of stay  BMI  VR-12 mental score  VR-12 physical score  Duration of operation (minutes)  Age  Historical knee procedures  SEIFA  VR-12 pain score  Transfusion during surgery | 5000 trees | Train = 0.677 |
| **k = 11**  Length of stay  BMI  VR-12 mental score  VR-12 physical score  Duration of operation (minutes)  Age  Historical knee procedures  SEIFA  VR-12 pain score  Transfusion during surgery  Pre-discharge complication | 5000 trees | Train = 0.697 |
| **k = 12**  Length of stay  BMI  VR-12 mental score  VR-12 physical score  Duration of operation (minutes)  Age  Historical knee procedures  SEIFA  VR-12 pain score  Transfusion during surgery  Pre-discharge complication  Charlson Comorbidity Index | 5000 trees | Train = 0.700* |
| **k = 13**  Length of stay  BMI  VR-12 mental score  VR-12 physical score  Duration of operation (minutes)  Age  Historical knee procedures  SEIFA  VR-12 pain score  Transfusion during surgery  Pre-discharge complication  Charlson Comorbidity Index  Remoteness area | 5000 trees | Train = 0.697 |
| **k = 14**  Length of stay  BMI  VR-12 mental score  VR-12 physical score  Duration of operation (minutes)  Age  Historical knee procedures  SEIFA  VR-12 pain score  Transfusion during surgery  Pre-discharge complication  Charlson Comorbidity Index  Remoteness area  Hypertension | 5000 trees | Train = 0.698 |
| **k = 15**  Length of stay  BMI  VR-12 mental score  VR-12 physical score  Duration of operation (minutes)  Age  Historical knee procedures  SEIFA  VR-12 pain score  Transfusion during surgery  Pre-discharge complication  Charlson Comorbidity Index  Remoteness area  Hypertension  Sex | 5000 trees | Train = 0.696 |
| **k = 16**  Length of stay  BMI  VR-12 mental score  VR-12 physical score  Duration of operation (minutes)  Age  Historical knee procedures  SEIFA  VR-12 pain score  Transfusion during surgery  Pre-discharge complication  Charlson Comorbidity Index  Remoteness area  Hypertension  Sex  Pensioner concession card | 5000 trees | Train = 0.695 |
| **k = 17**  Length of stay  BMI  VR-12 mental score  VR-12 physical score  Duration of operation (minutes)  Age  Historical knee procedures  SEIFA  VR-12 pain score  Transfusion during surgery  Pre-discharge complication  Charlson Comorbidity Index  Remoteness area  Hypertension  Sex  Pensioner concession card  Cancer (any) | 5000 trees | Train = 0.695 |
| **k = 18**  Length of stay  BMI  VR-12 mental score  VR-12 physical score  Duration of operation (minutes)  Age  Historical knee procedures  SEIFA  VR-12 pain score  Transfusion during surgery  Pre-discharge complication  Charlson Comorbidity Index  Remoteness area  Hypertension  Sex  Pensioner concession card  Cancer (any)  Time in ICU (days) | 5000 trees | Train = 0.696 |
| **k = 19**  Length of stay  BMI  VR-12 mental score  VR-12 physical score  Duration of operation (minutes)  Age  Historical knee procedures  SEIFA  VR-12 pain score  Transfusion during surgery  Pre-discharge complication  Charlson Comorbidity Index  Remoteness area  Hypertension  Sex  Pensioner concession card  Cancer (any)  Time in ICU (days)  Admissions in the past 12 months | 5000 trees | Train = 0.691 |
| **k = 20**  Length of stay  BMI  VR-12 mental score  VR-12 physical score  Duration of operation (minutes)  Age  Historical knee procedures  SEIFA  VR-12 pain score  Transfusion during surgery  Pre-discharge complication  Charlson Comorbidity Index  Remoteness area  Hypertension  Sex  Pensioner concession card  Cancer (any)  Time in ICU (days)  Admissions in the past 12 months  Interpreter | 5000 trees | Train = 0.693 |
| **k = 21**  Length of stay  BMI  VR-12 mental score  VR-12 physical score  Duration of operation (minutes)  Age  Historical knee procedures  SEIFA  VR-12 pain score  Transfusion during surgery  Pre-discharge complication  Charlson Comorbidity Index  Remoteness area  Hypertension  Sex  Pensioner concession card  Cancer (any)  Time in ICU (days)  Admissions in the past 12 months  Interpreter  Diabetes | 5000 trees | Train = 0.693 |
| **k = 22**  Length of stay  BMI  VR-12 mental score  VR-12 physical score  Duration of operation (minutes)  Age  Historical knee procedures  SEIFA  VR-12 pain score  Transfusion during surgery  Pre-discharge complication  Charlson Comorbidity Index  Remoteness area  Hypertension  Sex  Pensioner concession card  Cancer (any)  Time in ICU (days)  Admissions in the past 12 months  Interpreter  Diabetes  COPD | 5000 trees | Train = 0.694 |
| **k = 23**  Length of stay  BMI  VR-12 mental score  VR-12 physical score  Duration of operation (minutes)  Age  Historical knee procedures  SEIFA  VR-12 pain score  Transfusion during surgery  Pre-discharge complication  Charlson Comorbidity Index  Remoteness area  Hypertension  Sex  Pensioner concession card  Cancer (any)  Time in ICU (days)  Admissions in the past 12 months  Interpreter  Diabetes  COPD  Depression | 5000 trees | Train = 0.694 |
| **k = 24**  Length of stay  BMI  VR-12 mental score  VR-12 physical score  Duration of operation (minutes)  Age  Historical knee procedures  SEIFA  VR-12 pain score  Transfusion during surgery  Pre-discharge complication  Charlson Comorbidity Index  Remoteness area  Hypertension  Sex  Pensioner concession card  Cancer (any)  Time in ICU (days)  Admissions in the past 12 months  Interpreter  Diabetes  COPD  Depression  High risk of infection | 5000 trees | Train = 0.691 |
| **k = 25**  Length of stay  BMI  VR-12 mental score  VR-12 physical score  Duration of operation (minutes)  Age  Historical knee procedures  SEIFA  VR-12 pain score  Transfusion during surgery  Pre-discharge complication  Charlson Comorbidity Index  Remoteness area  Hypertension  Sex  Pensioner concession card  Cancer (any)  Time in ICU (days)  Admissions in the past 12 months  Interpreter  Diabetes  COPD  Depression  High risk of infection  Emergency presentations in the past 12 months | 5000 trees | Train = 0.689 |
| *Highest training set performance; VR-12 = Veteran’s RAND 12-item Health Survey; BMI = body mass index; SEIFA = Socioeconomic Indexes for Areas; ICU = intensive care unit; HDU = high dependency unit; ED = emergency department; CHF = congestive heart failure | | |

## Performance of random forest model trained on k12 highest importance variables – (Figures S21-S23, Table S26, Figure S24)

### Figure S21: Training set ROC curve for k25 random forest model


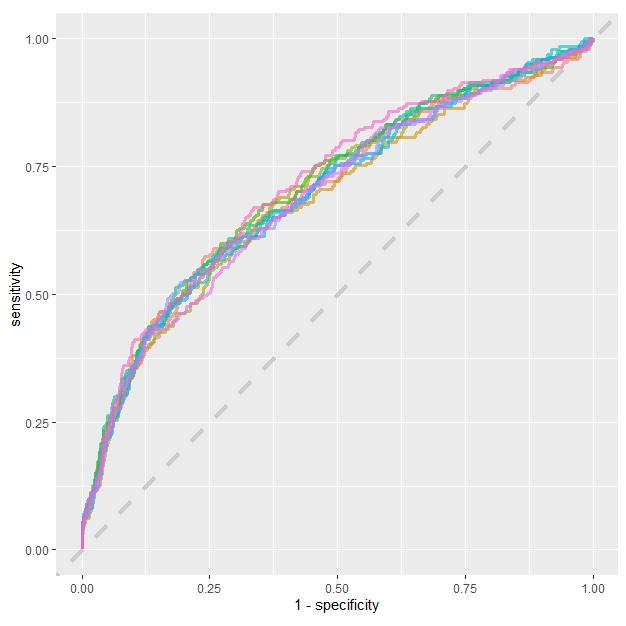


### Figure S22: Test set ROC curve for k12 random forest model


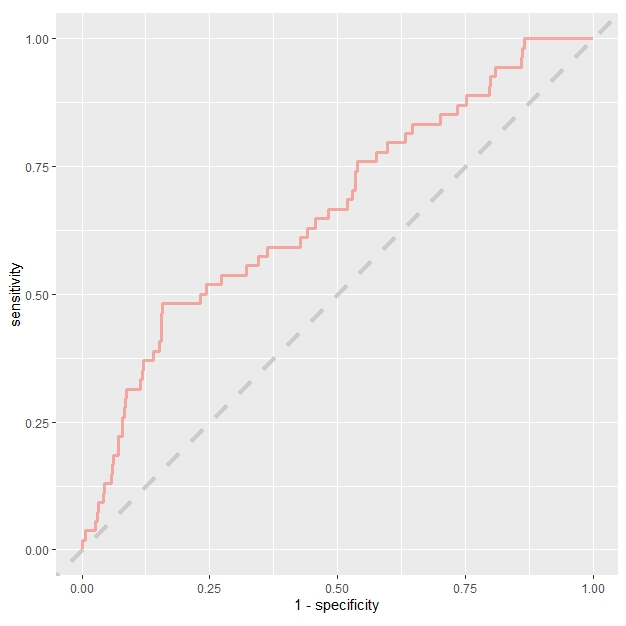


### Figure S23: Precision-recall (PPV-sensitivity) curve for k12 random forest model


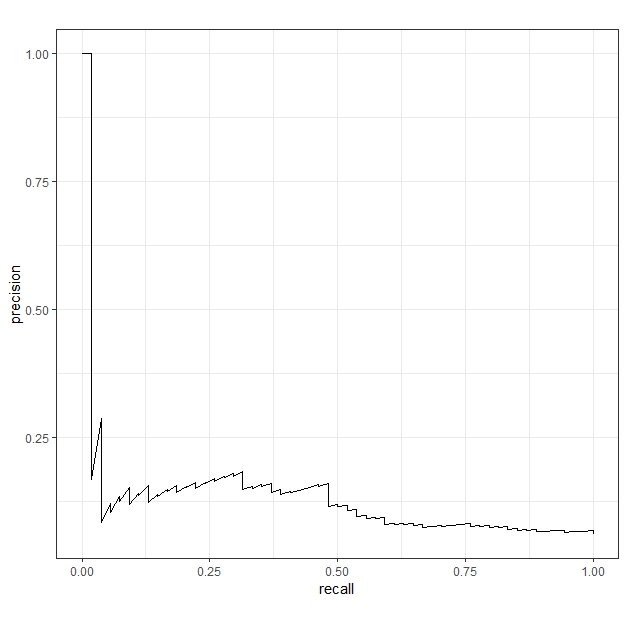


### Table S26: Performance metrics for k12 random forest model

| **Thresholds** | **Performance metrics** |
| --- | --- |
| Optimal threshold to maximise MCC = 0.129 | True positives = 26  True negatives = 730  False negatives = 28  False positives = 137  MCC = 0.199  Sensitivity (recall) = 0.481  Specificity = 0.842  Youden index = 0.323  F statistic = 0.240  PPV (precision) = 0.160  NPV = 0.963 |
| Threshold-free metrics | Discrimination:  AUC ROC (test set) = 0.666 (0.588 to 0.743)  AUC PR = 0.130  Calibration:  Slope = 0.593 (0.332 to 0.854)  Intercept = -0.350 (-0.636 to -0.064)  ICI = 0.029  E50 = 0.018  E90 = 0.059  Emax = 0.169 |
| MCC = Matthews Correlation Coefficient; PPV = positive predictive value; NPV = negative predictive value; AUC ROC = Area under the receiver operating characteristic curve; AUC PR = Area under the precision-recall (PPV-sensitivity) curve; ICI = Integrated calibration index; E50 = median of the absolute difference between observed and predicted probabilities; E90 = 90th percentile of the absolute difference between observed and predicted probabilities; Emax = maximal absolute difference between observed and predicted probabilities of the outcome | |

### Figure S24: Calibration curve for k12 random forest model


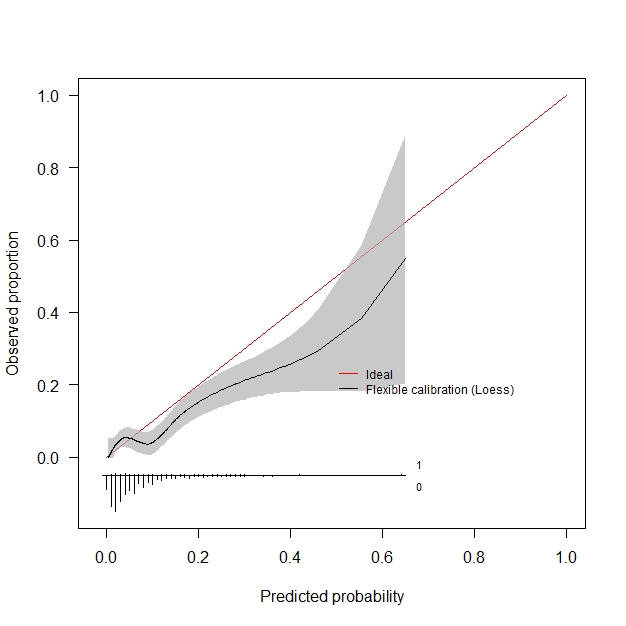


## Performance of k12 Random Forest model using only Initial consultation variables (Table S27, Figures S25-S27, Table S28, Figure S28)

### Table S27: Predictors and model specification for k12 model using only Initial consultation variables

| **Predictors** | **Specifications** | **AUC** |
| --- | --- | --- |
| **k = 20 (i.e. this is the k = 25 model but with the non-Initial consultation variables removed)**  Length of stay  BMI  VR-12 mental score  VR-12 physical score  Duration of operation (minutes)  Age  Historical knee procedures  SEIFA  VR-12 pain score  Transfusion during surgery  Pre-discharge complication  Charlson Comorbidity Index  Remoteness area  Hypertension  Sex  Pensioner concession card  Cancer (any)  Time in ICU (days)  Admissions in the past 12 months  Interpreter | 5000 trees | Train = 0.628  Test = 0.594 (95% confidence interval: 0.516 to 0.672) |
| VR-12 = Veteran’s RAND 12-item Health Survey; BMI = body mass index; SEIFA = Socioeconomic Indexes for Areas; | | |

### Figure S25: Training set ROC curve for k12 random forest model using only Initial consultation variables


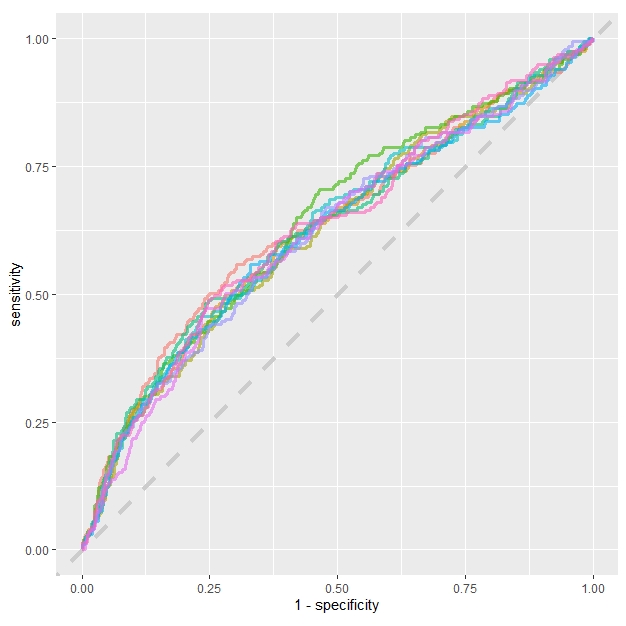


### Figure S26: Test set ROC curve for k12 random forest model using only Initial consultation variables


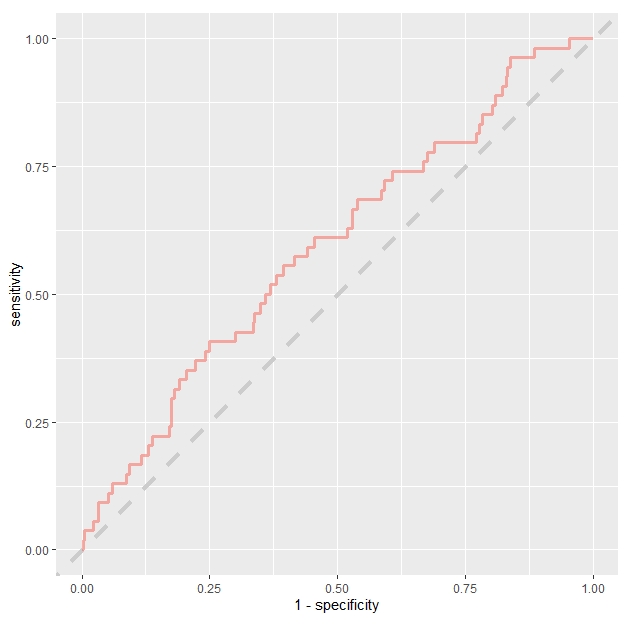


### Figure S27: Precision-recall (PPV-sensitivity) curve for k12 random forest model using only Initial consultation variables


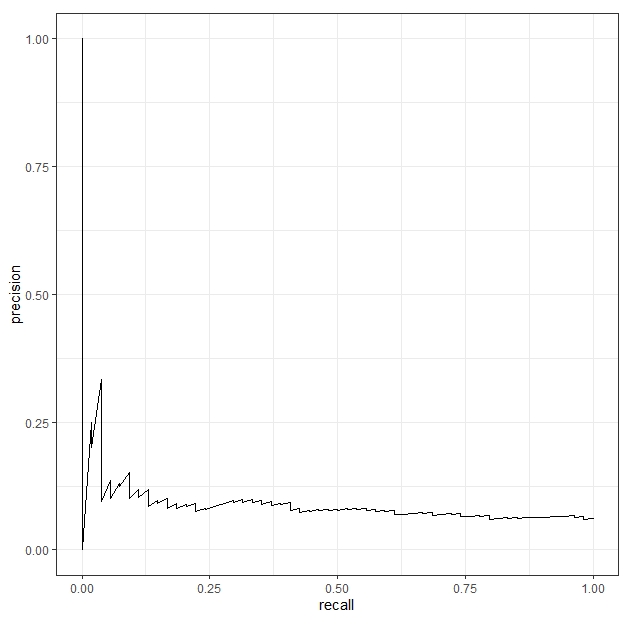


### Table S28: Performance metrics for k12 random forest model using only Initial consultation variables

| **Thresholds** | **Performance metrics** |
| --- | --- |
| Optimal threshold to maximise MCC = 0.383 | True positives = 2  True negatives = 863  False negatives = 52  False positives = 4  MCC = 0.095  Sensitivity (recall) = 0.037  Specificity = 0.995  Youden index = 0.032  F statistic = 0.067  PPV (precision) = 0.333  NPV = 0.943 |
| Threshold-free metrics | Discrimination:  AUC PR = 0.085  Calibration:  Slope = 0.370 (0.079 to 0.661)  Intercept = -0.328 (-0.612 to -0.045)  ICI = 0.032  E50 = 0.017  E90 = 0.090  Emax = 0.269 |
| MCC = Matthews Correlation Coefficient; PPV = positive predictive value; NPV = negative predictive value; AUC ROC = Area under the receiver operating characteristic curve; AUC PR = Area under the precision-recall (PPV-sensitivity) curve; ICI = Integrated calibration index; E50 = median of the absolute difference between observed and predicted probabilities; E90 = 90th percentile of the absolute difference between observed and predicted probabilities; Emax = maximal absolute difference between observed and predicted probabilities of the outcome | |

### Figure S28: Calibration curve for k12 random forest model using only Initial consultation variables


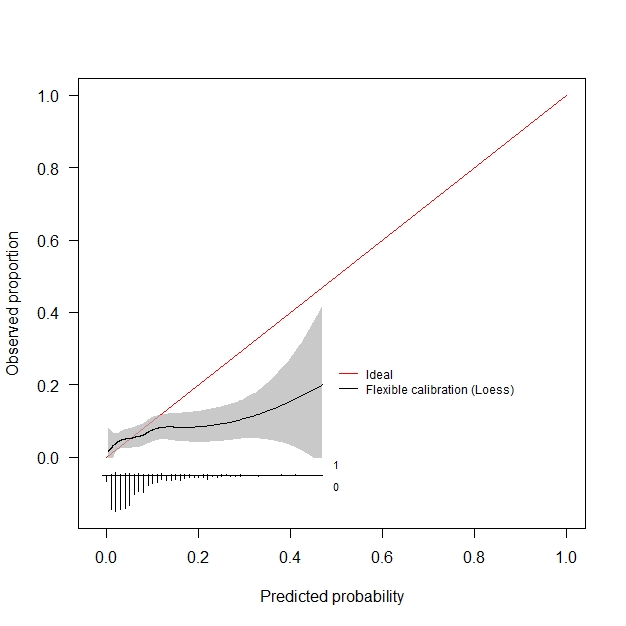


## Performance of Logistic Regression model trained on all predictors (Table S29, Figures S29-S31, Table S30, Figure S32, Table S31)

### Table S29: Model specifications and AUC ROC performance for logistic regression model trained on all available predictors

| **Model** | **Predictors** | **Model specifications** | **AUC** |
| --- | --- | --- | --- |
| All predictors (i.e. systematic review predictors, as well as all high- and moderate-importance variables through Delphi and focus group) | Congestive heart failure; Liver disease; Dementia; Charlson Comorbidity Index = 1; Substance abuse; Preoperative pain level; Admissions in the last 12 months; Pre-discharge complication (any type of complication); Days in ICU; Return to theatre prior to discharge; Preoperative mental function; Preoperative physical function; Peripheral vascular disease; Diabetes; Thrombocytopenia (proxy for coagulopathy); BMI; Age; Length of stay; Chronic kidney disease; Arrhythmia; Smoking; Pulmonary disease; Duration of operation (minutes); Low SES; Wound class; Depression; Previous stroke; Anaemia; History of cancer; High risk of infection; Remoteness area (major cities of Australia) – proxy for poor access to post-op care; Interpreter required; Emergency presentations in the last 12 months; Number of prior knee procedures; Male sex; Hypertension; Transfusion during surgery | Logistic regression | Train = 0.677  Test = 0.644 (95% confidence interval: 0.553 to 0.734) |
| ICU = intensive care unit; BMI = body mass index; SES = socioeconomic status | | | |

### Figure S29: Training set ROC curve for logistic regression model trained on all available predictors


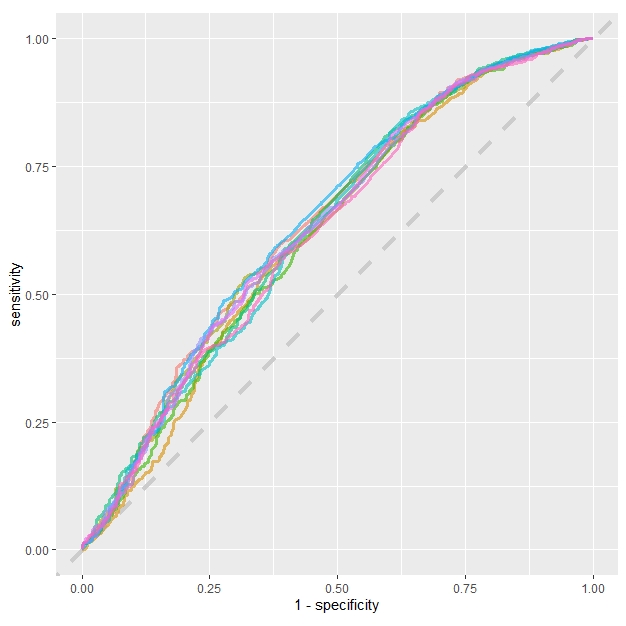


### Figure S30: Test set ROC curve for logistic regression model trained on all available predictors


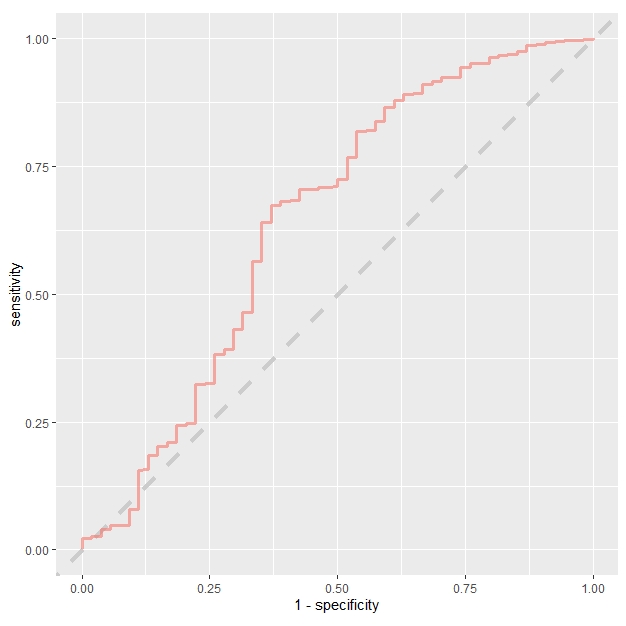


### Figure S31: Precision-recall (PPV-sensitivity) curve for random forest model trained on all available predictors


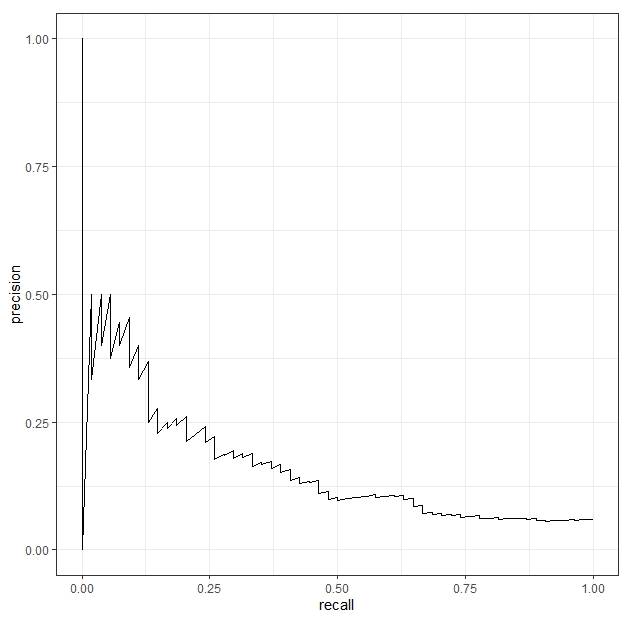


### Table S30: Performance metrics for logistic regression model trained on all available predictors

| **Thresholds** | **Performance metrics** |
| --- | --- |
| Optimal threshold to maximise MCC = 0.122 | True positives = 13  True negatives = 826  False negatives = 41  False positives = 41  MCC = 0.193  Sensitivity (recall) = 0.241  Specificity = 0.953  Youden index = 0.193  F statistic = 0.241  PPV (precision) = 0.241  NPV = 0.953 |
| Threshold-free metrics | Discrimination:  AUC PR = 0.156  Calibration:  Slope = 0.950 (0.506 to 1.393)  Intercept = -0.216 (-0.496 to 0.065)  ICI = 0.022  E50 = 0.021  E90 = 0.033  Emax = 0.943 |
| MCC = Matthews Correlation Coefficient; PPV = positive predictive value; NPV = negative predictive value; AUC ROC = Area under the receiver operating characteristic curve; AUC PR = Area under the precision-recall (PPV-sensitivity) curve; ICI = Integrated calibration index; E50 = median of the absolute difference between observed and predicted probabilities; E90 = 90th percentile of the absolute difference between observed and predicted probabilities; Emax = maximal absolute difference between observed and predicted probabilities of the outcome | |

### Figure S32: Calibration curve for logistic regression model trained on all available predictors


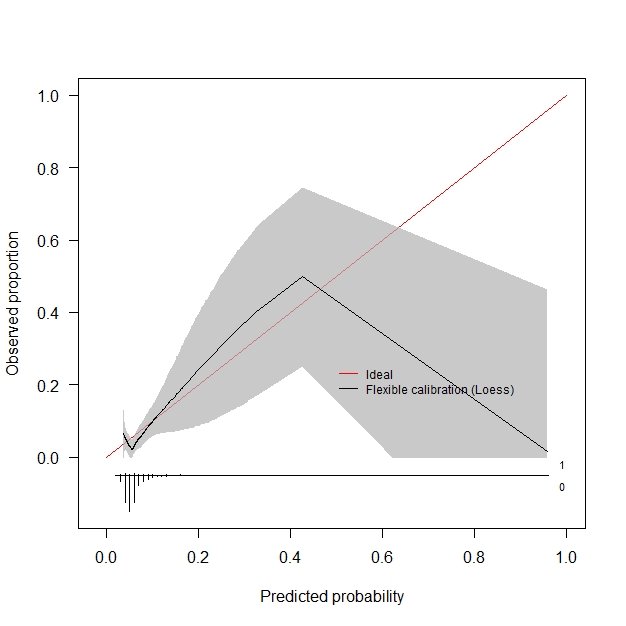


### Table S31: Coefficients for logistic regression model trained on all available predictors

| **Variable** | **Coefficients** |
| --- | --- |
| (Intercept) | -2.675 |
| VR-12 pain score | 0 |
| VR-12 mental score | 0 |
| VR-12 physical score | 0 |
| BMI | 0.020 |
| Age | 0 |
| Length of stay | 0.243 |
| Duration of operation (minutes) | 0 |
| High risk of infection | 0.007 |
| Congestive heart failure | 0 |
| Liver disease | 0 |
| Dementia | 0 |
| Charlson Comorbidity Index (one) | 0 |
| Charlson Comorbidity Index (two or more) | 0.052 |
| Substance abuse | 0 |
| Admissions in the past 12 months (one) | 0 |
| Admissions in the past 12 months (two) | 0 |
| Admissions in the past 12 months (three or more) | 0 |
| Pre-discharge complication | 0.174 |
| Time in ICU (days) (one) | 0 |
| Time in ICU (days) (two or more) | 0 |
| Return to theatre | 0 |
| Peripheral vascular disease | 0 |
| Diabetes | 0.009 |
| Diabetes with end-organ damage | 0 |
| Coagulopathy | 0 |
| Chronic kidney disease | 0.007 |
| Arrhythmia | 0 |
| Smoker | 0 |
| COPD | 0.029 |
| Pensioner concession card | 0.001 |
| SEIFA (two) | 0 |
| SEIFA (three) | 0 |
| SEIFA (four) | 0 |
| SEIFA (five) | 0 |
| SEIFA (six) | 0 |
| SEIFA (seven) | 0 |
| SEIFA (eight) | 0 |
| SEIFA (nine) | 0 |
| SEIFA (ten) | 0.053 |
| Wound condition (dirty) | 0 |
| Depression | 0 |
| Stroke | 0 |
| Anaemia | 0.043 |
| Cancer (any) | 0 |
| Remoteness area (major cities of Australia) | 0 |
| Remoteness area (outer regional or remote Australia) | 0 |
| Interpreter | 0 |
| Emergency presentations in the past 12 months (one) | 0.036 |
| Emergency presentations in the past 12 months (two or more) | 0 |
| Historical knee procedures (one) | 0 |
| Historical knee procedures (two) | 0 |
| Historical knee procedures (three or more) | 0.286 |
| Sex (male) | 0 |
| Hypertension | 0 |
| Transfusion during surgery (one) | 0 |
| Transfusion during surgery (two) | 0 |
| Transfusion during surgery (three or more) | 0.090 |
| VR-12 = Veteran’s RAND 12-item Health Survey; BMI = body mass index; CHF = congestive heart failure; ICU = intensive care unit; HDU = high dependency unit; CKD = chronic kidney disease; SEIFA = Socioeconomic Indexes for Areas; ED = emergency department | |

## Performance of Logistic Regression model trained on all predictors available at Initial consultation (Table S32, Figures S33-S35, Table S33, Figure S36, Table S34)

### Table S32: Model specifications and AUC ROC performance for logistic regression model trained on all predictors available at Initial consultation

| **Model** | **Predictors** | **Model specifications** | **AUC** |
| --- | --- | --- | --- |
| All predictors available at Initial consultation (i.e. systematic review predictors, as well as all high- and moderate-importance variables through Delphi and focus group) | Congestive heart failure; Liver disease; Dementia; Charlson Comorbidity Index = 1; Substance abuse; Preoperative pain level; Admissions in the last 12 months; Preoperative mental function; Preoperative physical function; Peripheral vascular disease; Diabetes; Thrombocytopenia (proxy for coagulopathy); BMI; Age; Chronic kidney disease; Arrhythmia; Smoking; Pulmonary disease; Low SES; Depression; Previous stroke; Anaemia; History of cancer; High risk of infection; Remoteness area (major cities of Australia) - proxy for poor access to post-op care; Interpreter required; Emergency presentations in the last 12 months; Number of prior knee procedures; Male sex; Hypertension | Logistic regression | Train = 0.595  Test = 0.592 (95% CI: 0.513 to 0.672) |
| ICU = intensive care unit; BMI = body mass index; SES = socioeconomic status | | | |

### Figure S33: Training set ROC curve for logistic regression model trained on all predictors available at Initial consultation


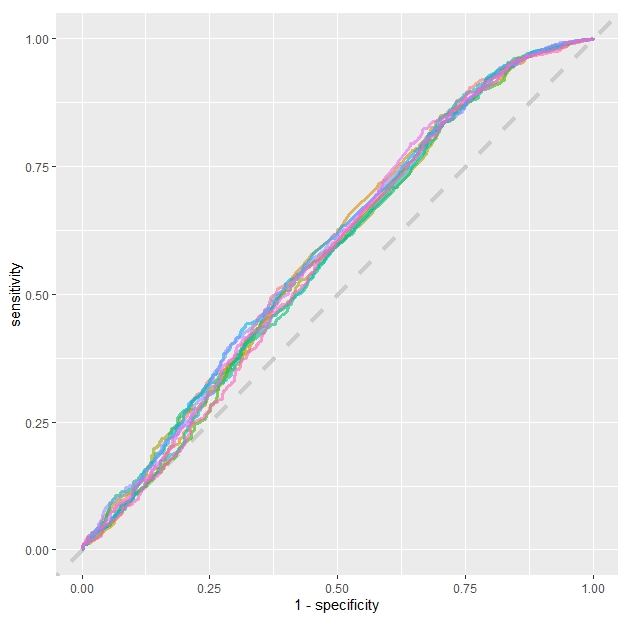


### Figure S34: Test set ROC curve for logistic regression model trained on all predictors available at Initial consultation


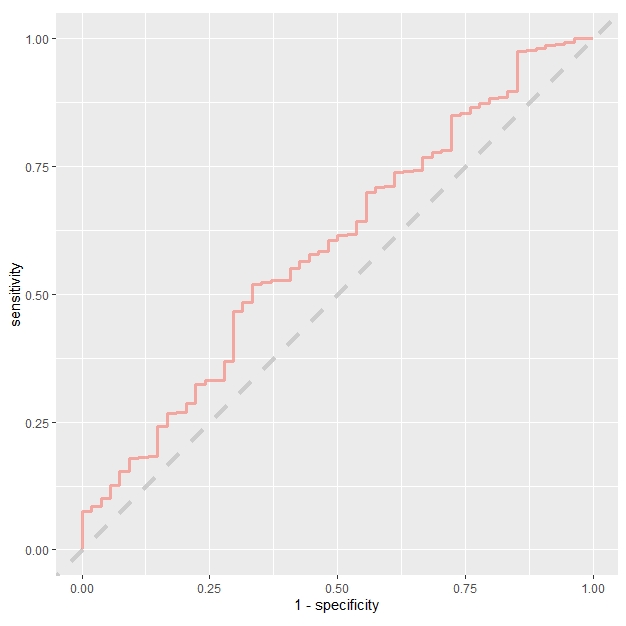


### Figure S37: Precision-recall (PPV-sensitivity) curve for random forest model trained on all predictors available at Initial consultation


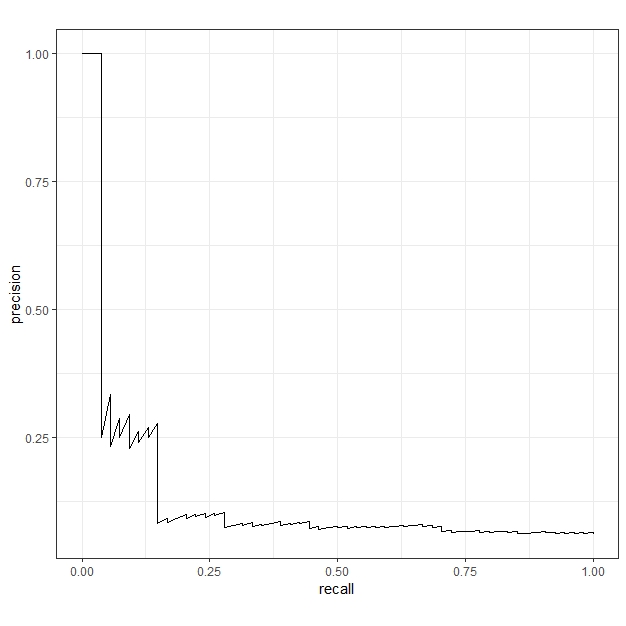


### Table S33: Performance metrics for logistic regression model trained on all predictors available at Initial consultation

| **Thresholds** | **Performance metrics** |
| --- | --- |
| Optimal threshold to maximise MCC = 0.443 | True positives = 2  True negatives = 867  False negatives = 52  False positives = 0  MCC = 0.187  Sensitivity (recall) = 0.037  Specificity = 1.000  Youden index = 0.037  F statistic = 0.071  PPV (precision) = 1  NPV = 0.943 |
| Threshold-free metrics | Discrimination:  AUC PR = 0.130  Calibration:  Slope = 0.674 (0.267 to 1.081)  Intercept = -0.226 (-0.506 to 0.054)  ICI = 0.016  E50 = 0.003  E90 = 0.046  Emax = 0.151 |
| MCC = Matthews Correlation Coefficient; PPV = positive predictive value; NPV = negative predictive value; AUC ROC = Area under the receiver operating characteristic curve; AUC PR = Area under the precision-recall (PPV-sensitivity) curve; ICI = Integrated calibration index; E50 = median of the absolute difference between observed and predicted probabilities; E90 = 90th percentile of the absolute difference between observed and predicted probabilities; Emax = maximal absolute difference between observed and predicted probabilities of the outcome | |

### Figure S36: Calibration curve for logistic regression model trained on all predictors available at Initial consultation


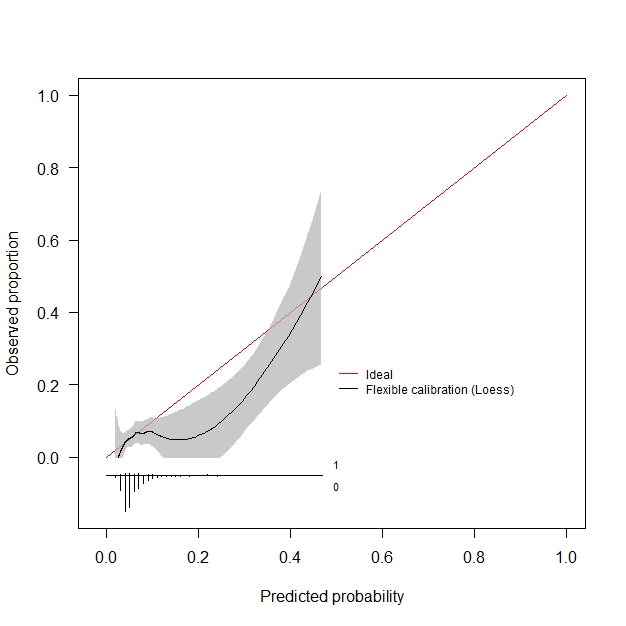


### Table S34: Coefficients for logistic regression model trained on all available predictors

| **Variable** | **Coefficients** |
| --- | --- |
| (Intercept) | -2.706 |
| VR-12 pain score | 0.010 |
| VR-12 mental score | -0.059 |
| VR-12 physical score | 0 |
| BMI | 0.117 |
| Age | 0 |
| High risk of infection | 0.011 |
| Congestive heart failure | 0.019 |
| Liver disease | -0.045 |
| Dementia | -0.050 |
| Charlson Comorbidity Index (one) | 0 |
| Charlson Comorbidity Index (two or more) | 0 |
| Substance abuse | -0.001 |
| Admissions in the past 12 months (one) | 0 |
| Admissions in the past 12 months (two) | -0.002 |
| Admissions in the past 12 months (three or more) | 0.023 |
| Peripheral vascular disease | 0.004 |
| Diabetes | 0.106 |
| Diabetes with end-organ damage | 0 |
| Coagulopathy | -0.059 |
| Chronic kidney disease | 0.089 |
| Arrhythmia | -0.058 |
| Smoker | -0.011 |
| COPD | 0.116 |
| Pensioner concession card | 0.052 |
| SEIFA (two) | -0.006 |
| SEIFA (three) | -0.077 |
| SEIFA (four) | 0 |
| SEIFA (five) | 0 |
| SEIFA (six) | 0.014 |
| SEIFA (seven) | 0 |
| SEIFA (eight) | 0.081 |
| SEIFA (nine) | 0.030 |
| SEIFA (ten) | 0.150 |
| Depression | 0 |
| Stroke | 0.024 |
| Anaemia | 0.081 |
| Cancer (any) | 0.122 |
| Remoteness area (major cities of Australia) | 0.014 |
| Remoteness area (outer regional or remote Australia) | -0.028 |
| Interpreter | -0.057 |
| Emergency presentations in the past 12 months (one) | 0.094 |
| Emergency presentations in the past 12 months (two or more) | 0 |
| Historical knee procedures (one) | -0.040 |
| Historical knee procedures (two) | 0 |
| Historical knee procedures (three or more) | 0.330 |
| Sex (male) | 0.020 |
| Hypertension | -0.020 |
| VR-12 = Veteran’s RAND 12-item Health Survey; BMI = body mass index; CHF = congestive heart failure; ICU = intensive care unit; HDU = high dependency unit; CKD = chronic kidney disease; SEIFA = Socioeconomic Indexes for Areas; ED = emergency department | |

## Predictor summary statistics SMART-only dataset (Table S35)

| **Feature** | **Non-readmitted cases (n = 5193)** | **Readmitted cases (n = 374)** | **P-value** |
| --- | --- | --- | --- |
| Age (mean (SD)) | 69.754 (8.771) | 70.104 (8.666) | 0.451 |
| Sex (% female) | 64.857% | 60.160% | 0.075 |
| BMI (mean (SD)) | 32.910 (6.303) | 34.150 (7.538) | 0.002 |
| Charlson Comorbidity Index | Zero = 2720 (52.378%)  One = 1470 (28.307%)  ≥Two = 1003 (19.314%) | Zero = 177 (47.326%)  One = 98 (26.203%) ≥Two = 99 (26.203%) | 0.004 |
| In-hospital complication (any) | 685 (13.191%) | 113 (30.214%) | <0.001 |
| Peripheral vascular disease | 178 (3.428%) | 18 (4.813%) | 0.208 |
| Diabetes | No = 4072 (78.413%)  Diabetes = 1105 (21.279%) Diabetes with end-organ damage = 16 (00.308%) | No = 276 (73.797%)  Diabetes = 97 (25.936%) Diabetes with end-organ damage = 1 (00.267%) | 0.087 |
| Length of stay (mean (SD)) | 5.148 (2.617) | 6.072 (3.785) | <0.001 |
| Low SES | 1 = 539 (10.379%)  2 = 348 (6.701%)  3 = 378 (7.279%)  4 = 391 (7.529%)  5 = 582 (11.207%)  6 = 385 (7.414%)  7 = 900 (17.331%)  8 = 621 (11.958%)  9 = 736 (14.173%)  10 = 313 (6.027%) | 1 = 30 (8.021%)  2 = 24 (6.417%)  3 = 20 (5.348%)  4 = 28 (7.487%)  5 = 33 (8.824%)  6 = 31 (8.289%)  7 = 64 (17.112%)  8 = 42 (11.230%)  9 = 67 (17.914%)  10 = 35 (9.358%) | 0.075 |
| Depression | 541 (10.418%) | 44 (11.765%) | 0.464 |
| History of cancer | 490 (9.436%) | 41 (10.963%) | 0.379 |
| Patient-related biopsychosocial: lower education level, poor health literacy, non-English speaking | 864 (16.638%)  Missing = 43 (0.828%) | 51 (13.636%) Missing = 5 (1.337%) | 0.320 |
| Hypertension | 3422 (65.896%) | 236 (63.102%) | 0.297 |
| *"how much did pain interfere with your normal work?" - 1 = Not at all; 2 = A little bit; 3 = Moderately; 4 = Quite a bit; 5 = Extremely; SES = socioeconomic status | | | |

## Missingness per variable - SMART-only dataset (Table S36)

| **Variable** | **Missingness in SMART** |
| --- | --- |
| Age (mean (SD)) | 0 |
| Sex (% female) | 0 |
| BMI (mean (SD)) | 0 |
| Charlson Comorbidity Index | 0 |
| In-hospital complication (any) | 0 |
| Peripheral vascular disease | 0 |
| Diabetes | 0 |
| Length of stay (mean (SD)) | 0 |
| Low SES | 0 |
| Depression | 0 |
| History of cancer | 0 |
| Patient-related biopsychosocial: lower education level, poor health literacy, non-English speaking | 0.862% |
| Hypertension | 0 |
| BMI = body mass index; CHF = congestive heart failure; ICU = intensive care unit; HDU = high dependency unit; CKD = chronic kidney disease; SES = socioeconomic status | |

## SMART-only dataset cohort creation flow diagram – Figure S37


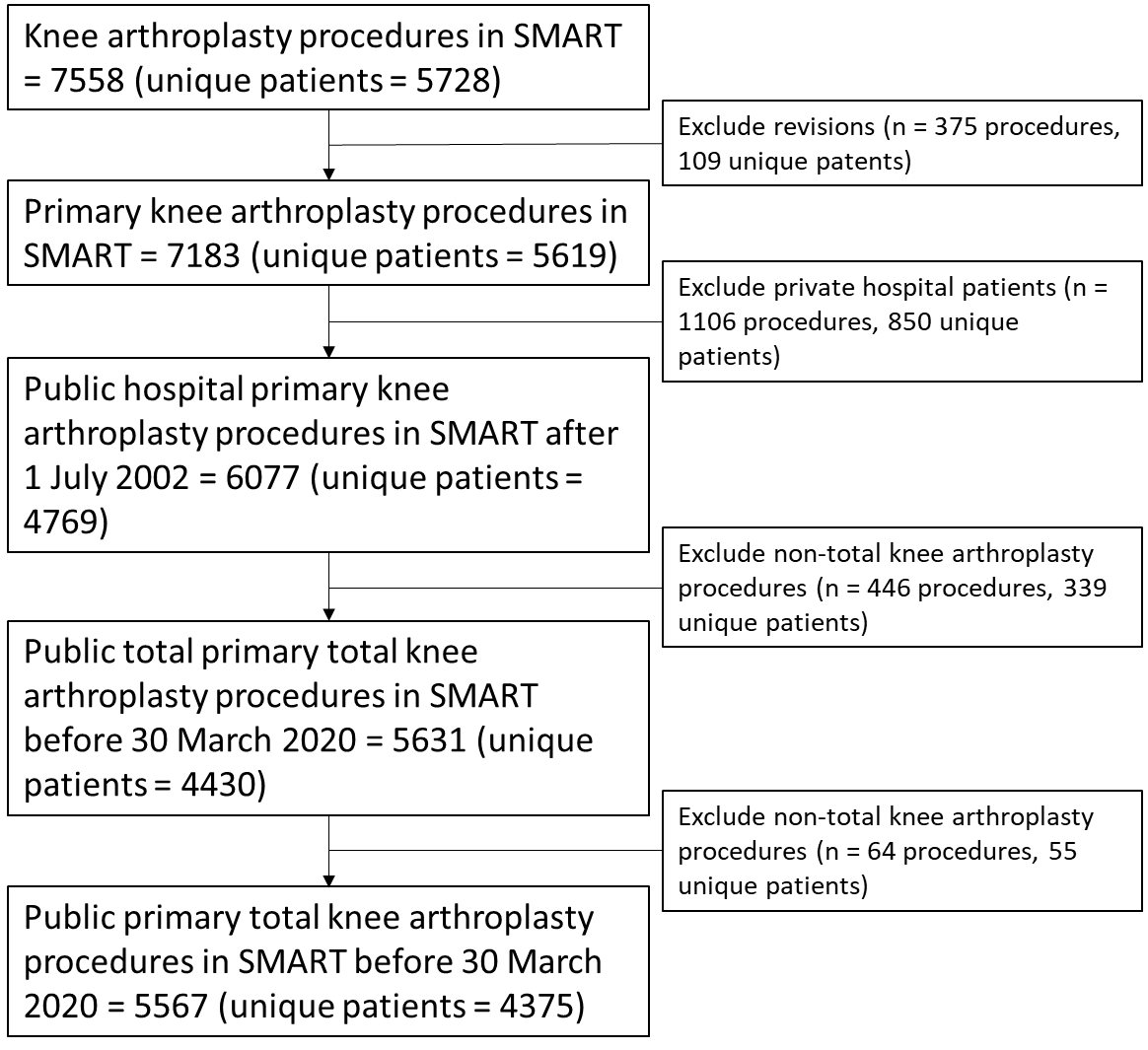


## Random Forest model performance - SMART discharge model (Table S37, Figures S38-S40, Table S38, Figure S41, Table S39)

### Table S37: Variables, model specifications, and AUC for SMART-only model

| **Model** | **Predictors** | **Model specifications** | **AUC** |
| --- | --- | --- | --- |
| All predictors (i.e. my selection of variables based on systematic review, as well as all high- and moderate-importance variables through Delphi and focus group) | BMI  Age  Length of stay  SEIFA  Charlson Comorbidity Index  Hypertension  Diabetes  In-hospital Complication (any)  Sex  History of cancer  Interpreter required  Peripheral vascular disease  Depression | Random forest  5000 trees | Train = 0.641  Test = 0.592 (95% confidence interval: 0.527 to 0.658) |
| BMI = body mass index; SEIFA = Socioeconomic Indexes for Areas | | | |

### Figure S38: Training set ROC curve for SMART-only random forest discharge model


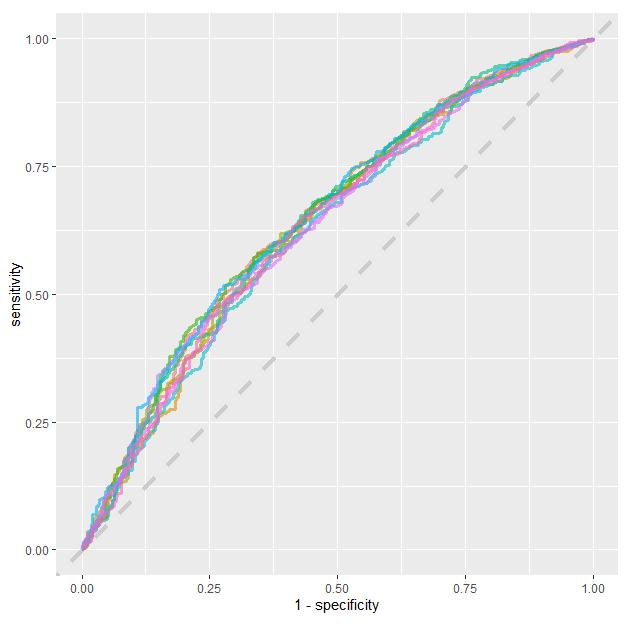


### Figure S39: Test set ROC curve for SMART-only random forest discharge model


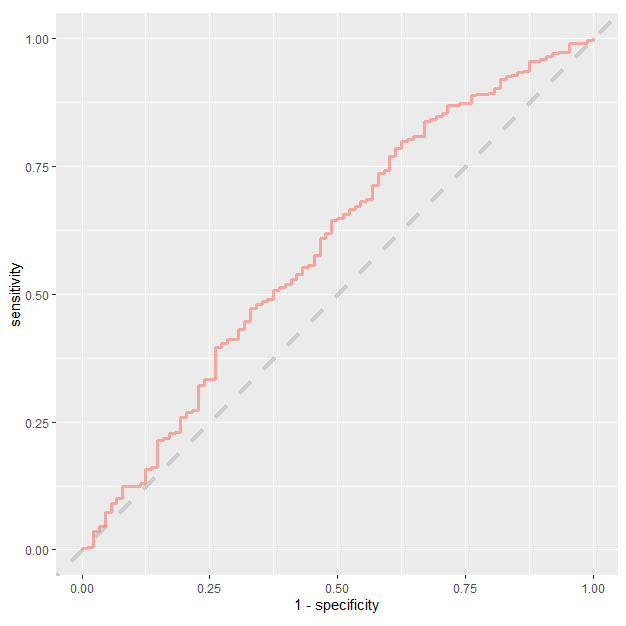


### Figure S40: Precision-recall (PPV-sensitivity) curve for SMART-only random forest discharge model


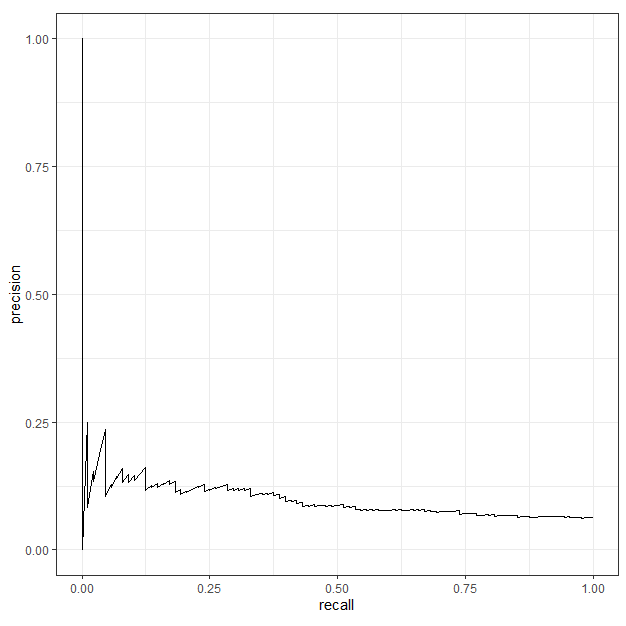


### Table S38: Performance metrics for SMART-only random forest discharge model

| **Thresholds** | **Performance metrics** |
| --- | --- |
| Optimal threshold to maximise MCC = 0.122 | True positives = 65  True negatives = 1135  False negatives = 63  False positives = 169  MCC = 0.109  Sensitivity (recall) = 0.284  Specificity = 0.870  Youden index = 0.154  F statistic = 0.177  PPV (precision) = 0.278  NPV = 0.947 |
| Threshold-free metrics | Discrimination:  AUC ROC (training set) = 0.641  AUC ROC (test set) = 0.592 (95% CI: 0.527 to 0.658)  AUC PR = 0.095  Calibration:  Slope = 0.426 (95% CI: 0.152 to 0.700)  Intercept = -0.080 (95% CI: -0.300 to 0.141)  ICI = 0.023  E50 = 0.020  E90 = 0.041  Emax = 0.246 |
| MCC = Matthews Correlation Coefficient; PPV = positive predictive value; NPV = negative predictive value; AUC ROC = Area under the receiver operating characteristic curve; AUC PR = Area under the precision-recall (PPV-sensitivity) curve; ICI = Integrated calibration index; E50 = median of the absolute difference between observed and predicted probabilities; E90 = 90th percentile of the absolute difference between observed and predicted probabilities; Emax = maximal absolute difference between observed and predicted probabilities of the outcome | |

### Figure S41: Calibration curve for SMART-only random forest discharge model


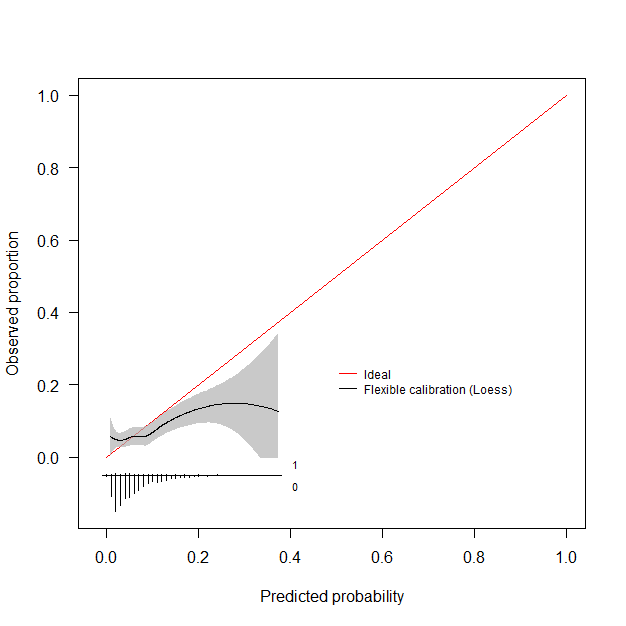


### Table S39: Variable importance factors for SMART-only random forest discharge model

| **Variable** | **Importance (descending order)** |
| --- | --- |
| BMI | 78.490 |
| Age | 54.650 |
| Length of stay | 30.436 |
| SEIFA | 30.301 |
| Charlson Comorbidity Index | 10.960 |
| In-hospital complication (any) | 8.242 |
| Hypertension | 7.549 |
| Sex | 7.408 |
| Diabetes | 6.470 |
| Interpreter required | 6.191 |
| History of cancer | 5.700 |
| Depression | 5.616 |
| Peripheral vascular disease | 4.465 |
| BMI = body mass index; SEIFA = Socioeconomic Indexes for Areas; | |

## Random Forest model performance - SMART Initial consultation model (Table S40, Figures S42-S44, Table S41, Figure S45, Table S42)

### Table S40: Variables, model specifications, and AUC for SMART-only random forest Initial consultation model

| **Model** | **Predictors** | **Model specifications** | **AUC** |
| --- | --- | --- | --- |
| All predictors (i.e. my selection of variables based on systematic review, as well as all high- and moderate-importance variables through Delphi and focus group) | BMI  Age  SEIFA  Charlson Comorbidity Index  Hypertension  Diabetes  Sex  History of cancer  Interpreter required  Peripheral vascular disease  Depression | Random forest  5000 trees | Train = 0.591  Test = 0.525 (95% confidence interval: 0.460 to 0.590) |
| BMI = body mass index; SEIFA = Socioeconomic Indexes for Areas | | | |

### Figure S42: Training set ROC curve for SMART-only random forest Initial consultation model


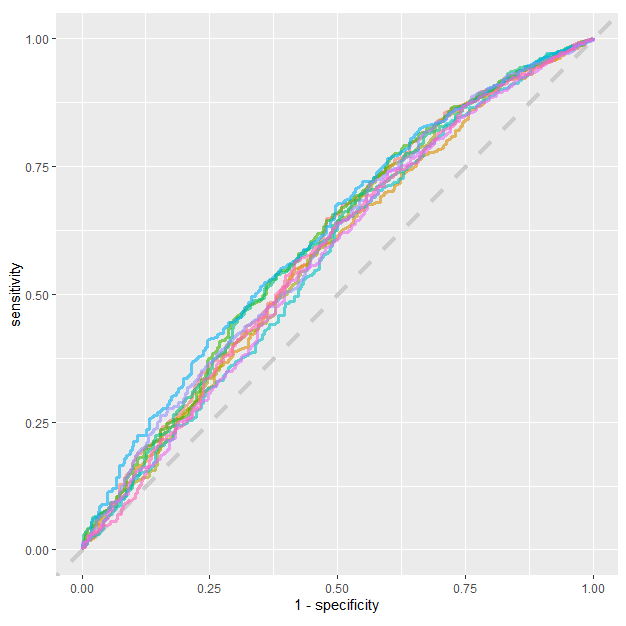


### Figure S43: Test set ROC curve for SMART-only random forest Initial consultation model


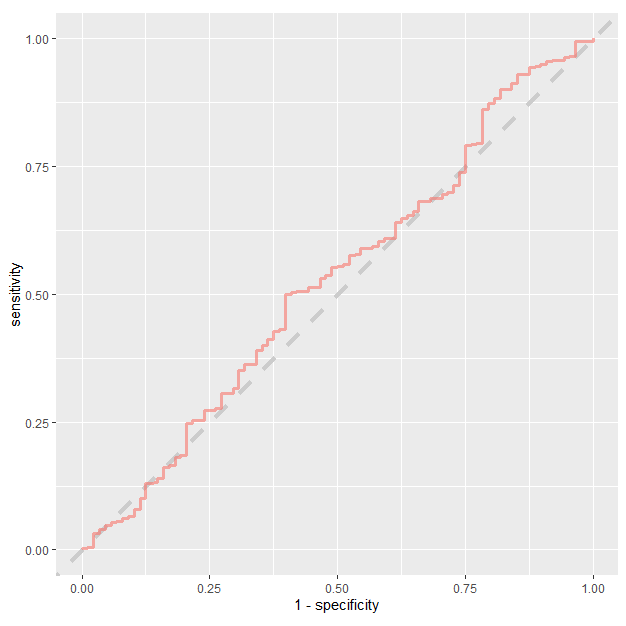


### Figure S44: Precision-recall (PPV-sensitivity) curve for SMART-only random forest Initial consultation model


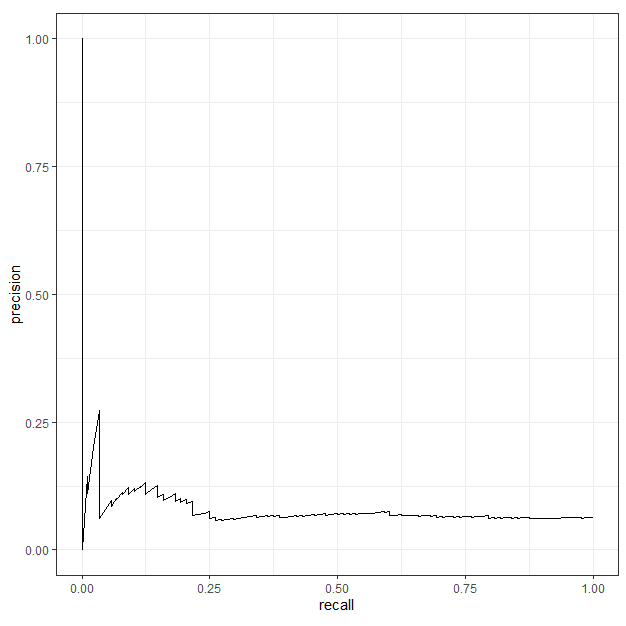


### Table S41: Performance metrics for SMART-only random forest Initial consultation model

| **Thresholds** | **Performance metrics** |
| --- | --- |
| Optimal threshold to maximise MCC = 0.272 | True positives = 3  True negatives = 1296  False negatives = 85  False positives = 8  MCC = 0.077  Sensitivity (recall) = 0.034  Specificity = 0.994  Youden index = 0.028  F statistic = 0.061  PPV (precision) = 0.273  NPV = 0.938 |
| Threshold-free metrics | Discrimination:  AUC ROC (training set) = 0.591  AUC ROC (test set) = 0.525 (95% CI: 0.460 to 0.590)  AUC PR = 0.076  Calibration:  Slope = 0.132 (95% CI: -0.153 to 0.418)  Intercept = -0.068 (95% CI: -0.289 to 0.152)  ICI = 0.035  E50 = 0.022  E90 = 0.075  Emax = 0.288 |
| MCC = Matthews Correlation Coefficient; PPV = positive predictive value; NPV = negative predictive value; AUC ROC = Area under the receiver operating characteristic curve; AUC PR = Area under the precision-recall (PPV-sensitivity) curve; ICI = Integrated calibration index; E50 = median of the absolute difference between observed and predicted probabilities; E90 = 90th percentile of the absolute difference between observed and predicted probabilities; Emax = maximal absolute difference between observed and predicted probabilities of the outcome | |

### Figure S45: Calibration curve for SMART-only random forest Initial consultation model


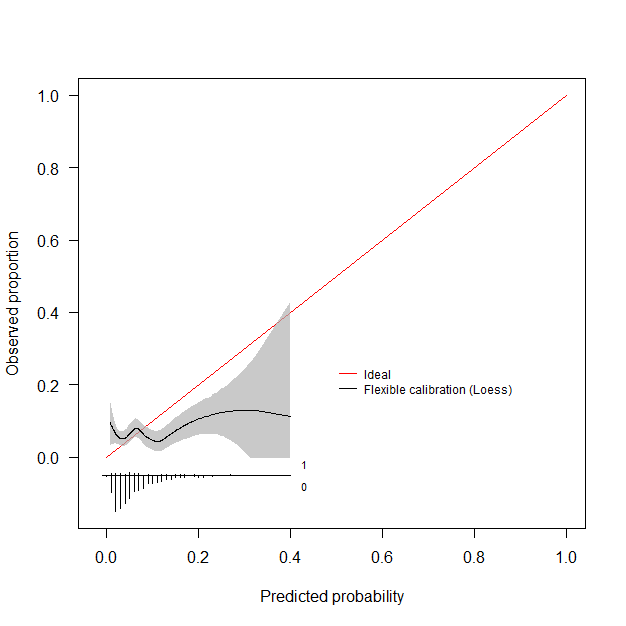


### Table S42: Variable importance factors for SMART-only random forest Initial consultation model

| **Variable** | **Importance (descending order)** |
| --- | --- |
| BMI | 91.887 |
| Age | 57.242 |
| SEIFA | 29.937 |
| Charlson Comorbidity Index | 10.108 |
| Hypertension | 7.932 |
| Sex | 6.782 |
| Diabetes | 6.431 |
| Interpreter required | 5.705 |
| History of cancer | 5.694 |
| Depression | 5.599 |
| Peripheral vascular disease | 4.179 |
| BMI = body mass index; SEIFA = Socioeconomic Indexes for Areas | |

## Cause-specific models

### Table S43: Outcome definitions and predictors for each outcome from the literature

| **Outcome (supporting literature)** | **Definition** | **Predictors** |
| --- | --- | --- |
| Surgical site infection [4-7] | Superficial infection and/or deep infection. In this study, we did not distinguish between the two.  In line with the literature, we excluded prosthetic joint infection from the definition of surgical site infection. | ASA class  Male sex  BMI  Malnutrition (using albumin or total lymphocyte level)  Low preoperative Hb  Diabetes mellitus  Rheumatoid arthritis  Preoperative UTI  Asymptomatic bacteriuria  Peripheral vascular disease  Chronic skin disease  Revision surgery  Smoking (and ex-smoker)  Alcohol abuse  Skin preparation technique  Prolonged surgery  Glove change  Antibiotic prophylaxis  Postoperative haematoma  Staph aureus decolonisation  Surgeon's experience  Low socioeconomic status (including Medicaid insurance and living in rural area)  Seasonal factors (Summer surgery increased risk)  Hypertension |
| Venous thromboembolism [5, 8, 9] | Deep vein thrombosis or pulmonary embolism, including both symptomatic and asymptomatic cases | Age  Sex  Ethnicity (specifically Asian and Pacific Islander)  Prior VTE  Thrombophilia (specifically factor V Leiden)  Malignancy  BMI  CHF  COAD (specifically with cor pulmonale)  Rheumatoid arthritis  Preoperative Hb  Haematologic disorder (including leukaemias)  Stroke with hemiparesis  Long hospital stay IN BED  Obesity (marked)  Early mobilisation  Thromboprophylaxis (mechanical)  Thromboprophylaxis (pharmacological)  Thromboprophylaxis (out of hospital)  Caprini score  Administrative databaset major surgery within the last month  Visible varicose veins within administrative databaset month  Inflammatory bowel disease within last month  Current lower limb oedema within last month  AMI within last month  Serious infection (e.g. pneumonia) within last month  Blood transfusion  Duration of surgery  On bed rest or restricted mobility  Family history of blood clots (thrombosis)  Paralysis  Spinal cord injury resulting in paralysis  Serious trauma (including serious falls history or MVA)  Broken hip, pelvis, or leg  Cemented prosthesis |
| Joint-specific complications [5, 10] | Ramkumar 2015: Traumatic and atraumatic dislocation; Prosthetic misalignment; Ligamentous laxity; Periprosthetic fracture; Septic joint, periprosthetic infection, or both  D'Apuzzo 2017: Acute myocardial infarction, Pneumonia, Sepsis/septicemia/shock, Surgical site bleeding, Pulmonary embolism, Death, Mechanical complications, Periprosthetic joint infection/wound infection  D’Apuzzo 2017 expanded: Acute myocardial infarction, Pneumonia, Sepsis/septicemia/shock, Surgical site bleeding, Pulmonary embolism, Death, Mechanical complications, Periprosthetic joint infection/wound infection,  Arterial embolism, Bleeding complication, Cardiac complication, Central nervous system complication other than stroke, Deep-vein thrombosis, Genitourinary complication, Hematological complication, Iatrogenic complication other than periprosthetic, Mechanical complication, Other complications, Nosocomial infection other than periprosthetic, Periprosthetic fracture or dislocation, Periprosthetic infection, Postoperative fever, Postoperative pain, Postoperative stiffness, Pulmonary complication other than pulmonary embolism, Soft-tissue complication, Stroke, Unspecified periprosthetic infection Wound complication, Revision | Age 65-75 yr  Age >85 yr  Male vs. female sex  Black race  Hispanic race  Medicare insurance  Medicaid insurance  In-hospital medical complications  In-hospital surgical complications  Congestive heart failure  Pulmonary circulation disorder  Other neurological disorder  Chronic pulmonary disease  Diabetes  Renal failure  Liver disease  Lymphoma  Coagulopathy  Obesity  Psychoses  Depression  Bilateral TKA |
| Infection (non-surgical site) [6, 11-15] | Urinary tract infection, sepsis, pneumonia, Clostridium Difficile colitis, and any other non-surgical site infection captured in our databases. | Female sex  Steroid use  Age  ASA class  Pre-op Cr >1.35  Duration of operation  Pre-op transfusion  Anaemia  Platelets <150000  Diabetes  Obesity  Dependent functional status  Hypertension  COPD  Dyspnea on exertion |
| Gastrointestinal complications [6, 16, 17] | Adenikinju 2019: Patients with secondary surgical procedures including colonoscopy, sigmoidoscopy, laparotomy, laparoscopy, or esophagogastroduodenoscopy within 30 days following TJA  Massaglia 2021: International Classification of Diseases, tenth revision, codes for infection, Clostridium difficile, ulcer, hemorrhage, gallstones, pancreatitis, constipation, obstruction, diarrhea, malabsorption, ischemia, liver failure, and miscellaneous digestive disorders | Peptic ulcer disease  Steroid use  Age  Hypotension  Smoking  Prior abdominal surgery  Narcotic pain medication  Patient-controlled analgesia  Male sex  DVT prophylaxis other than ASA (aspirin) or Warfarin  Former alcohol use  Regional anaesthesia |
| Cardiovascular complications [5, 6, 18] | Elsiwy 2019: cardiac arrest (CA), myocardial infarction (MI), or new-onset arrhythmia | Age  Sex  Any prior cardiac disorder  BMI  Hypertension  Diabetes  Bilateral TKA  Cerebrovascular disease  COPD  Low albumin  Anaemia  Smoking  ASA class |

### Table S44: Causes of readmission in this study cohort

| **Outcome category for primary cause of readmission (n)** | **Proportion of total readmissions (out of 251 total readmissions %)** |
| --- | --- |
| Surgical site infection (78) | 31.076% |
| Venous thromboembolism (40) | 15.936% |
| Joint-specific complication (70) | 27.888% |
| Infection (non-surgical site infection) (7) | 2.789% |
| Gastrointestinal complications (7) | 2.789% |
| Cardiac complications (6) | 2.390% |
| Other (43) | 17.131% |

### Table S45: Variable generation – outcomes

| Outcome* | Relevant complications used to generate this outcome variable | Number of events | Model developed |
| --- | --- | --- | --- |
| Surgical site infection – narrow definition | Blisters  Cellulitis  dehiscence  infection - deep wound  infection – pinsite  infection - superficial wound  infection - deep wound  stitch abscess  superficial wound infection  suture granuloma  wound blister  wound dehiscence | 62 | Yes |
| Surgical site infection – broad definition | Blisters  Cellulitis  decubitus ulcers  dehiscence  delayed wound healing  erythema  erythema – wound  infection - deep wound  infection – pinsite  infection - superficial wound  infection - deep wound  rash  stitch abscess  stitch reaction  superficial wound infection  suture granuloma  swelling  wound blister  wound dehiscence  wound edge necrosis  wound ooze  wound ooze delaying discharge  wound problems - other  wound retained foreign body | 84 | Yes |
| Venous thromboembolism – narrow definition | dvt  pe  pe/dvt  pulmonary embolism | 41 | Yes |
| Venous thromboembolism – broad definition | clot in kidney  dvt  epidural haematoma  haematoma  pe  pe/dvt  pulmonary embolism | 41 | No. There were no additional events above the narrow VTE definition. |
| Joint-specific complication – narrow definition | Traumatic and atraumatic dislocation;   - dislocation - dislocation – acute - dislocation - insert - dislocation – patella - dislocation – recurrent - prosthetic discloation   Prosthetic misalignment;   - malalignment   Ligamentous laxity;   - mcl medial laxity   Periprosthetic fracture;   - fracture – acetabulum - fracture - ankle - fracture – calcar - fracture - femoral condyle - fracture – femur - fracture - fibula - fracture - greater trochanter - fracture - intra-operative - fracture – ischium - fracture - orbital - fracture – patella - fracture - peri-prosthetic - fracture – periacetabular - fracture - superior rim - fracture – tibia - fracture - tibial plateau - fracture – wrist - peri-prosthetic fracture - stress fracture   Septic joint, periprosthetic infection, or both   - joint infection – acute - joint infection - haematogenous - joint infection – latent - prosthetic joint infection - pji   Acute myocardial infarction,   - ami   Pneumonia,   - pneumonia   Sepsis/septicemia/shock,   - sepsis - septic shower - septicaemia   Surgical site bleeding,   - haemarthrosis - haemathrosis - wound haematoma - wound ooze - wound ooze delaying discharge   Pulmonary embolism,   - pe - pe/dvt - pulmonary embolism   Death,   - death - surgery related   Mechanical complications,   - arthrofibrosis - asceptic loosening - acetabulum - asceptic loosening - femoral component - asceptic loosening - femoral stem - asceptic loosening – patella - asceptic loosening - tibial component - aseptic loosening - aseptic loosening – acetabulum - aseptic loosening - femoral component - aseptic loosening - femoral stem - aseptic loosening – patella - aseptic loosening - tibial component - ffd - fixation/hardware failure - implant loosening - instability - malalignment - mcl medial laxity - mcl tear - migration of prosthesis - patella maltracking - patella tendon rupture - pcl tear - poor rom - ruptured mcl - ruptured patella tendon - ruptured quadraceps - stiffness   Periprosthetic joint infection/wound infection   - cellulitis - decubitus ulcers - dehiscence - delayed wound healing - erythema - erythema - wound - infection - deep wound - infection – pinsite - infection - superficial wound - infection - deep wound - joint infection – acute - joint infection - haematogenous - joint infection – latent - pji - prosthetic joint infection - stitch abscess - stitch reaction - superficial ssi - superficial wound infection - suture granuloma - wound blister - wound dehiscence - wound edge necrosis - wound retained foreign body | 153 | Yes |
| Joint-specific complication – broad definition | Acute myocardial infarction,   - ami   Pneumonia,   - pneumonia   Sepsis/septicemia/shock,   - sepsis - septic shower - septicaemia   Surgical site bleeding,   - haemarthrosis - haemathrosis - wound haematoma - wound ooze - wound ooze delaying discharge   Pulmonary embolism,   - pe - pe/dvt - pulmonary embolism   Death,   - death - surgery related   Mechanical complications,   - arthrofibrosis - asceptic loosening - acetabulum - asceptic loosening - femoral component - asceptic loosening - femoral stem - asceptic loosening – patella - asceptic loosening - tibial component - aseptic loosening - aseptic loosening – acetabulum - aseptic loosening - femoral component - aseptic loosening - femoral stem - aseptic loosening – patella - aseptic loosening - tibial component - ffd - fixation/hardware failure - implant loosening - instability - malalignment - mcl medial laxity - mcl tear - migration of prosthesis - patella maltracking - patella tendon rupture - pcl tear - poor rom - ruptured mcl - ruptured patella tendon - ruptured quadraceps - stiffness   Periprosthetic joint infection/wound infection   - cellulitis - decubitus ulcers - dehiscence - delayed wound healing - erythema - erythema - wound - infection - deep wound - infection – pinsite - infection - superficial wound - infection - deep wound - joint infection – acute - joint infection - haematogenous - joint infection – latent - pji - prosthetic joint infection - stitch abscess - stitch reaction - superficial ssi - superficial wound infection - suture granuloma - wound blister - wound dehiscence - wound edge necrosis - wound retained foreign body   Arterial embolism,   - arterial occlusion   Bleeding complication,   - anaemia - acute due to surgical blood loss - blood loss - blood vessel cut - haematoma - haemorrhage - post-operative anaemia - ruptured genicular artery - surgical blood loss   Cardiac complication,   - af - ami - cardiac arrest - cardiac arrhythmia - nstemi - rapid af - svt - tachy-brady syndrome - exacerbation ccf - cardiogenic pulmonary oedema   Central nervous system complication other than stroke,   - acopia - alcohol withdrawal - confusion - delirium - drowsiness - drowsy - narcosed - opioid overdose - reduced gcs - seizure   Deep-vein thrombosis,   - dvt - pe/dvt   Genitourinary complication,   - acute urinary retention - haematuria - hydrocoele - hydronephrosis - post-catherisation haematuria - thrush - urinary incontinence - urinary retention - urinary stricture - uti   Hematological complication,   - dic - electrolyte imbalance - hyperkalaemia - hypokalaemia - hyponatraemia - hyponatremia (chronic) - pancytopenia - thrombocytopenia - transfusion reaction   Iatrogenic complication other than periprosthetic,   - blood vessel cut - breach of acetabular wall - drain tube-incomplete removal - dural puncture - instruments inadvertently contaminated - nerve injury - femoral - nerve injury – peroneal - nerve injury – sciatic - popliteal artery injury - post-catherisation haematuria - pressure sore – hip - pressure sore - lower limb - pressure sore – sacrum - retained drain tube   Other complications,   - airway swelling - aki - allergic skin reaction - anaphylaxis - bullous phemphigoid - burn - cement extravasation - drug reaction - exac asthma - exac bronchiectasis - exac coad - exacerbation of ra - excematous rash - fall - fat embolus - fat necrosis - granuloma - herpes simplex - hyperglycaemia - hyperparathyroidism - hypoglycaemia - hypoglycaemic - hypoxia - infected iv site - infected sebaceous cyst - ischaemic leg - neurological - neuropathic pain - neuropraxia - neuropraxia - femoral nerve - neuropraxia - lateral femoral cutaneous nerve - neuropraxia - sciatic nerve - pneumothorax - pseudo gout - unstable diabetes - unverified   Nosocomial infection other than periprosthetic,   - acute peritonitis - c diff infection - clostridium difficle colitis   Periprosthetic fracture or dislocation,   - fracture – acetabulum - fracture - ankle - fracture – calcar - fracture - femoral condyle - fracture – femur - fracture - fibula - fracture - greater trochanter - fracture - intra-operative - fracture – ischium - fracture - orbital - fracture – patella - fracture - peri-prosthetic - fracture – periacetabular - fracture - superior rim - fracture – tibia - fracture - tibial plateau - fracture – wrist - dislocation - dislocation – acute - dislocation - insert - dislocation – patella - dislocation – recurrent - peri-prosthetic fracture - prosthetic discloation - stress fracture   Periprosthetic infection,   - joint infection – acute - joint infection - haematogenous - joint infection – latent - prosthetic joint infection - pji   Postoperative fever,   - post-operative fever - pyrexia of unknown origin   Postoperative pain,   - neuropathic pain - pain   Postoperative stiffness,   - stiffness - poor rom - ffd   Pulmonary complication other than pulmonary embolism,   - apo - atelectasis - cardiogenic pulmonary oedema - chest infection - empyema - exac asthma - exac bronchiectasis - exac coad - lrti - lung nodule - pneumonia - pneumothorax - pulmonary oedema - respiratory acidosis - tracheal stenosis - urti   Soft-tissue complication,   - abductor tear - avulsion gluteous - compartment syndrome - detachment of hip abductors - gastrocnemius rupture - ischaemic leg - lower limb ischaemia - mcl tear - patella tendon rupture - pcl tear - ruptured mcl - ruptured patella tendon - ruptured quadraceps - skin tear   Stroke,   - cva - stroke - tia   Wound complication,   - wound blister - wound dehiscence - wound edge necrosis - wound haematoma - wound ooze - wound ooze delaying discharge - wound problems - other - wound retained foreign body - blisters - dehiscence - delayed wound healing - erythema – wound - infection - deep wound - infection – pinsite - infection - superficial wound - infection - deep wound - stitch abscess - stitch reaction - superficial ssi - superficial wound infection - suture granuloma   Revision   - dxfailure - dxfailure_aseptic_loosening - dxfailure_structural _implant_failure - dxfailure_tibiofemoral_instability - asceptic loosening - acetabulum - asceptic loosening - femoral component - asceptic loosening - femoral stem - asceptic loosening – patella - asceptic loosening - tibial component - aseptic loosening - aseptic loosening – acetabulum - aseptic loosening - femoral component - aseptic loosening - femoral stem - aseptic loosening – patella - aseptic loosening - tibial component   Prosthetic misalignment;   - malalignment   Ligamentous laxity;   - mcl medial laxity   Septic joint, periprosthetic infection, or both   - ALREADY INCLUDED ABOVE (in PJI) | 217 | Yes |
| Infection (non-surgical site) – narrow definition | acute peritonitis  c diff infection  chest infection  cholecystitis  clostridium difficle colitis  diverticulitis  empyema  infected iv site  infected sebaceous cyst  lrti  pancreatitis  pneumonia  sepsis  septic shower  septicaemia  shingles  urti  uti | 10 | No – not enough events. |
| Gastrointestinal complications – narrow definition | infection   - acute peritonitis - cholecystitis - diverticulitis - gastroenteritis - pancreatitis   Clostridium difficile   - c diff infection - clostridium difficle colitis   ulcer   - duodenal ulcer - oesophageal ulceration - perforated duodenal ulcer - pyloric ulcer perforation   hemorrhage   - bleeding – pr - bleeding oesophageal varices - gi bleeding - haematemesis - malaena - mallory-weiss tear   gallstones   - cholecystitis   pancreatitis   - pancreatitis   constipation   - constipation   obstruction   - bowel obstruction - ileus - paralytic ileus - pseudo bowel obstruction - sigmoid volvules   diarrhea   - diahorrea - diarrhoea   ischemia   - ischaemic hepatitis | 8 | No – not enough events |
| Gastrointestinal complications – broad definition | acute peritonitis  ascites  bleeding – pr  bleeding oesophageal varices  bowel obstruction  bowel perforation  c diff infection  cholecystitis  clostridium difficle colitis  constipation  derranged lfts  diahorrea  diarrhoea  diverticulitis  duodenal ulcer  gastroenteritis  gi bleeding  gord  haematemesis  ileus  ischaemic hepatitis  malaena  mallory-weiss tear  oesophageal ulceration  oesophagitis  pancreatitis  paralytic ileus  perforated duodenal ulcer  pseudo bowel obstruction  pyloric ulcer perforation  sigmoid volvules  vomiting | 13 | No – not enough events |
| Cardiac complications – narrow definition | af  ami  cardiac arrest  cardiac arrhythmia  nstemi  rapid af  svt  tachy-brady syndrome  exacerbation ccf  cardiogenic pulmonary oedema | 6 | No – not enough events |
| Cardiac complications – broad definition | af  ami  angina  apo  arterial occlusion  asystole  bradycardia  cardiac arrest  cardiac arrhythmia  cardiogenic pulmonary oedema  ccf  chest pain  digitoxin toxicity  exacerbation ccf  fluid overload  heart block  hypertension  hypotension  hypotension/presyncopal episodes  hypoxia  nstemi  pericardial effusion  peripheral oedema  ppm  pulmonary oedema  rapid af  svt  syncope  tachy-brady syndrome  tachycardia  takutsubo cardiomyopathy  vasovagal | 12 | No – not enough events |
| Combined outcome | Any of the above complications | 227 | Yes |
| *Two versions of each outcome were developed: a narrow definition that only included complications identified in prior literature, and a broad definition including additional related complications to increase the number of outcome events on which models were trained. | | | |

### Variable generation – predictors

#### Table S46: Surgical site infection (same predictors for narrow and broad definition)

| **Variable** | **Variable generation process** |
| --- | --- |
| Peripheral vascular disease | Variable exists in Registry |
| Diabetes mellitus | Variable exists in Registry |
| BMI | Variable exists in Registry |
| Malnutrition (using albumin or total lymphocyte level) | Unavailable |
| Low preoperative Hb | Variable exists in Registry |
| Rheumatoid arthritis | Variable exists in Registry |
| Smoking (and ex-smoker) | Variable exists in Registry |
| Preoperative UTI | Variable exists in Registry |
| Asymptomatic bacteriuria | Unavailable |
| Chronic skin disease | Variable exists in Registry (generated a proxy variable based on all chronic skin diseases available in the registry) |
| Prolonged surgery | Variable exists in administrative database, and Registry. Used duration of operation (minutes) as a proxy for this |
| Revision surgery | Unavailable in this cohort (only primary TKA surgeries) |
| Low socioeconomic status (including Medicaid insurance and living in rural area) | Used SEIFA score in Registry, and Pensioner Card Holder in administrative database. Rurality = In administrative database and SMART, used ASGC remoteness code. |
| Staph aureus decolonisation | Unavailable |
| Summer surgery | Variable exists in SMART and administrative database |
| Antibiotic prophylaxis | Unavailable |
| Alcohol abuse | Variable exists in SMART |
| Skin preparation technique | Unavailable |
| Glove change | Unavailable |
| Postoperative haematoma | Variable exists in Registry |
| Sex | Variable exists in Registry and administrative database |
| Hypertension | Variable exists in Registry |
| ASA class | Variable exists in SMART |
| Surgeon’s experience | Variable exists in SMART |
| CHF = congestive heart failure; ICU/HDU = intensive care unit/high dependency unit; BMI = body mass index; CKD = chronic kidney disease; SES = socioeconomic status; SEIFA = Socioeconomic Indexes for Areas; IVDU = intravenous drug use; emergency department = ED; SMART = St Vincent’s Melbourne Arthroplasty Outcomes Registry; VR-12 = Veteran’s RAND 12-item Health survey | |

#### Table S47: Venous thromboembolism (same predictors for narrow and broad definition)

| **Variable** | **Variable generation process** |
| --- | --- |
| CHF | Variable exists in Registry |
| BMI, obesity (marked) | Variable exists in Registry |
| Age | Variable exists in administrative database, and Registry |
| Ethnicity (specifically Asian and Pacific Islander) | Unavailable |
| Long hospital stay in bed | Unavailable |
| Early mobilisation | Unavailable |
| Prior VTE | Variable exists in Registry |
| Thrombophilia (specifically factor V Leiden) | Variable exists in Registry |
| Malignancy | Indicator variable for the occurrence of any of the following:   - Ovarian cancer - Uterine cancer - Thyroid cancer - Lung cancer - Endometrial cancer - Cervical cancer - Bladder cancer - Renal cancer - Melanoma - Prostate cancer - Bowel cancer - Breast cancer - Solid tumour (Charlson comorbidity) - Leukaemia (Charlson comorbidity) - Lymphoma (Charlson comorbidity) - Liposarcoma - Oesophageal cancer - Osteosarcoma - Cancer of the vocal cords - Hepatic cancer - Squamous cell carcinoma - Stomach cancer - Soft tissue cancer - Testicular cancer   Throat cancer |
| Stroke with hemiparesis | Variable exists in Registry (not enough observations to use this variable) |
| COAD (specifically with cor pulmonale) | Variable exists in Registry (but not specifically with cor pulmonale) |
| Paralysis | Variable exists in SMART (proxy generated = polio) |
| Visible varicose veins within administrative databaset month | Variable exists in SMART |
| Major surgery within administrative databaset month | Unavailable |
| Inflammatory bowel disease within last month | Variable exists in SMART |
| Current lower limb oedema within last month | Variable exists in SMART (proxy generated, not necessarily within the past month) |
| Blood transfusion | Variable exists in SMART (only blood transfusion during surgery is available) |
| AMI within last month | Variable exists in SMART (but not specifically within the past month) |
| Serious infection (e.g. pneumonia) within last month | Indicator variable for the occurrence of any variable available in the Registry which could increase risk of infection (either through the comorbidity itself, its treatment, and/or its sequelae):   - Rheumatoid arthritis - Connective tissue disorder - Ankylosing spondylitis - Autoimmune hepatitis - Crohn’s disease - Juvenile arthritis - Psoriasis - Psoriatic arthritis - Sarcoidosis - Scleroderma - Sjogren’s syndrome - Systemic lupus erythematosus - Temporal arteritis - Ulcerative colitis - Vasculitis - History of cancer (see previous variable) - Diabetes - AIDS - Neutropenia - Multiple myeloma - Osteomyelitis - Septic arthritis - Splenectomy - Smoker - Prosthetic joint infection |
| On bed rest or restricted mobility | Unavailable |
| Family history of blood clots (thrombosis) | Unavailable |
| Spinal cord injury resulting in paralysis | Unavailable |
| Sex | Variable exists in Registry and administrative database |
| Serious trauma (including serious falls history or MVA)/Broken hip, pelvis, or leg | Variable exists in Registry (not enough observations to use this predictor) |
| Cemented prosthesis | Variable exists in SMART |
| Rheumatoid arthritis | Variable exists in SMART |
| Preoperative haemoglobin | Variable exists in SMART |
| Haematologic disorder | Variable exists in SMART |
| Thromboprophylaxis (mechanical) | Variable exists in SMART |
| Thromboprophylaxis (pharmacological) | Variable exists in SMART |
| Thromboprophylaxis (out of hospital) | Variable exists in SMART (proxy = warfarin) |
| CHF = congestive heart failure; ICU/HDU = intensive care unit/high dependency unit; BMI = body mass index; CKD = chronic kidney disease; SES = socioeconomic status; SEIFA = Socioeconomic Indexes for Areas; IVDU = intravenous drug use; emergency department = ED; SMART = St Vincent’s Melbourne Arthroplasty Outcomes Registry; VR-12 = Veteran’s RAND 12-item Health survey | |

#### Table S48: Joint-specific complication – narrow definition (same predictors for narrow and broad definition)

| **Variable** | **Variable generation process** |
| --- | --- |
| CHF | Variable exists in Registry |
| Liver disease | Variable exists in Registry |
| Sex | Variable exists in Registry and administrative database |
| Black race | Unavailable |
| Hispanic race | Unavailable |
| Medicare insurance, Medicaid insurance | Proxy variable used in administrative database (pensioner concession card flag) |
| In-hospital surgical complications, in-hospital medical complications | Variable exists in Registry |
| Age | Variable exists in administrative database, and Registry |
| Pulmonary circulation disorder | Variable exists in Registry (at least, pulmonary circulatory disorder does) |
| Other neurological disorder | Variable exists in Registry |
| Diabetes | Variable exists in Registry |
| Coagulopathy | Variable exists in Registry. Used the comorbidity variable ‘thrombocytopenia’ |
| BMI | Variable exists in Registry |
| Chronic pulmonary disease | Variable exists in Registry |
| CKD | Variable exists in Registry |
| Lymphoma | Variable exists in Registry |
| Depression | Variable exists in SMART |
| Bilateral TKA | Variable exists in Registry and administrative database |
| CHF = congestive heart failure; ICU/HDU = intensive care unit/high dependency unit; BMI = body mass index; CKD = chronic kidney disease; SES = socioeconomic status; SEIFA = Socioeconomic Indexes for Areas; IVDU = intravenous drug use; emergency department = ED; SMART = St Vincent’s Melbourne Arthroplasty Outcomes Registry; VR-12 = Veteran’s RAND 12-item Health survey | |

#### Table S49: Infection (non-surgical site)

| **Variable** | **Variable generation process** |
| --- | --- |
| Sex | Variable exists in Registry and administrative database |
| Age | Variable exists in administrative database, and Registry |
| Duration of operation | Variable exists in administrative database, and Registry |
| Pre-op Cr >1.35 | Variable exists in Registry (used proxy variable, presence of chronic kidney disease or end-stage kidney disease) |
| Pre-op transfusion | Variable exists in Registry (best available was intra-operative transfusion) |
| Steroid use | Unavailable |
| Dependent functional status | Unavailable |
| Hypertension | Variable exists in Registry |
| Diabetes | Variable exists in Registry |
| COPD | Variable exists in Registry |
| Obesity | Variable exists in Registry |
| ASA class | Variable exists in Registry |
| Anaemia | Variable exists in Registry |
| Platelets <150000 | Unavailable |
| Dyspnea on exertion | Unavailable |
| CHF = congestive heart failure; ICU/HDU = intensive care unit/high dependency unit; BMI = body mass index; CKD = chronic kidney disease; SES = socioeconomic status; SEIFA = Socioeconomic Indexes for Areas; IVDU = intravenous drug use; emergency department = ED; SMART = St Vincent’s Melbourne Arthroplasty Outcomes Registry; VR-12 = Veteran’s RAND 12-item Health survey | |

#### Table S50: Gastrointestinal complications (same predictors for narrow and broad definition)

| **Variable** | **Variable generation process** |
| --- | --- |
| Sex | Variable exists in Registry and administrative database |
| Age | Variable exists in administrative database, and Registry |
| Former alcohol use | Variable exists in Registry |
| Steroid use | Unavailable |
| Peptic ulcer disease | Variable exists in Registry |
| Hypotension | Unavailable |
| Smoking | Variable exists in Registry |
| Prior abdominal surgery | Unavailable |
| Narcotic pain medication | Unavailable |
| Patient-controlled analgesia | Unavailable |
| DVT prophylaxis other than ASA (aspirin) or Warfarin | Variable exists in Registry |
| Regional anaesthesia | Variable exists in Registry |
| CHF = congestive heart failure; ICU/HDU = intensive care unit/high dependency unit; BMI = body mass index; CKD = chronic kidney disease; SES = socioeconomic status; SEIFA = Socioeconomic Indexes for Areas; IVDU = intravenous drug use; emergency department = ED; SMART = St Vincent’s Melbourne Arthroplasty Outcomes Registry; VR-12 = Veteran’s RAND 12-item Health survey | |

#### Table S51: Cardiac complications (same predictors for narrow and broad definition)

| **Variable** | **Variable generation process** |
| --- | --- |
| Age | Variable exists in administrative database, and Registry |
| BMI | Variable exists in Registry |
| Sex | Variable exists in Registry and administrative database |
| Any prior cardiac disorder | Variable exists in Registry |
| Hypertension | Variable exists in Registry |
| Diabetes | Variable exists in Registry |
| Bilateral TKA | Variable exists in Registry and administrative database |
| ASA class | Variable exists in Registry |
| Cerebrovascular disease | Variable exists in Registry |
| Anaemia | Variable exists in Registry |
| Smoking | Variable exists in Registry |
| Low albumin | Unavailable |
| COPD | Variable exists in Registry |
| CHF = congestive heart failure; ICU/HDU = intensive care unit/high dependency unit; BMI = body mass index; CKD = chronic kidney disease; SES = socioeconomic status; SEIFA = Socioeconomic Indexes for Areas; IVDU = intravenous drug use; emergency department = ED; SMART = St Vincent’s Melbourne Arthroplasty Outcomes Registry; VR-12 = Veteran’s RAND 12-item Health survey | |

#### Table S52: Variable preparation and missingness – predictors not described earlier in Table S3

| Feature | Preparation | Missingness |
| --- | --- | --- |
| Comorbidities | | |
| ASA class | Categorical (1, 2, 3, 4) | 0.678% |
| Rheumatoid arthritis | Dummy | 0 |
| Preoperative UTI | Dummy | 0 |
| Chronic skin condition | Dummy | 0 |
| Alcohol abuse | Dummy | 0 |
| Prior VTE | Dummy | 0 |
| Factor V Leiden | Dummy | 0 |
| Haematologic disorder | Dummy (any of: leukaemia, lymphoma, haemophilia) | 0 |
| Psychoses | Dummy (schizophrenia) | 0 |
| Thromboprophylaxis (out of hospital) | Dummy (warfarin use outside of hospital) | 0 |
| Varicose veins | Dummy | 0 |
| Inflammatory bowel disease | Dummy | 0 |
| Paralysis | Dummy (polio was the only relevant comorbidity available) | 0 |
| Pulmonary circulation disorder | Dummy (pulmonary hypertension was the only relevant comorbidity available) | 0 |
| Other neurological disorder | Dummy (any of: hemiplegia, dementia, past stroke, epilepsy, Parkinson’s, restless legs, vertigo, cerebral aneurysm, multiple sclerosis, Alzheimer disease, subarachnoid hemorrhage) | 0 |
| Lymphoma | Dummy | 0 |
| Peptic ulcer disease | Dummy | 0 |
| Any cardiac comorbidity | Dummy (any of: heart failure, arrhythmia, ischemic heart disease, atrial fibrillation, prior cardiac surgery, angina, aortic stenosis, pacemaker, aortic valve replacement, mitral valve replacement, cardiomyopathy, paroxysmal atrial fibrillation, supraventricular tachycardia, palpitations, coronary artery disease, prior acute myocardial infarction) | 0 |
| Variables related to index hospital admission | | |
| Preoperative haemoglobin (mean (SD)) | Continuous (numeric) | 14.844% |
| Surgeon experience* | NA | 0 |
| DVT prophylaxis other than warfarin | Dummy | 0 |
| Mechanical thromboprophylaxis | Dummy | 0 |
| Pharmacological thromboprophylaxis | Dummy | 0 |
| Anaesthetic - general | Dummy | 0 |
| Anaesthetic - epidural | Dummy | 0 |
| Anaesthetic - local | Dummy | 0 |
| Anaesthetic - spinal | Dummy | 0 |
| Anaesthetic - sedation | Dummy | 0 |
| Cemented prosthesis | Dummy (indicator for three cemented components (femoral, tibial, patella)) | 0 |
| Simultaneous bilateral TKA | Dummy | 0 |
| Summer surgery | Dummy | 0 |
| *Variable derived from SMART registry; **Variable derived from administrative database; ***”how much did pain interfere with your normal work?” – 1 = Not at all; 2 = A little bit; 3 = Moderately; 4 = Quite a bit; 5 = Extremely; **** Continuous variables were compared using Student’s t-test. Categorical variables were compared using chi-squared test, or Fisher’s exact test in cases of counts below 10; BMI = body mass index; SEIFA = Socioeconomic Indexes for Areas [19]; CHF = congestive heart failure; SES = socioeconomic status; IVDU = intravenous drug use; ED = emergency department | | |

#### Table S53: Comparison of baseline characteristics for participants with ≥10% missing data for given variable

| **Feature** | **Missing** | **Non-missing** | **P-value** |
| --- | --- | --- | --- |
| Age (mean (SD)) | 71.060 (8.185) | 69.307 (8.924) | <0.001 |
| Sex (% female) | 60.512% | 64.181% | 0.110 |
| BMI (mean (SD)) | 31.596 (6.336) | 33.405 (6.561) | <0.001 |
| Charlson Comorbidity Index | One = 171 (31.261%)  ≥Two = 91 (16.636%) | One = 855 (27.247%)  ≥Two = 675 (21.511%) | 0.070 |
| Combined outcome – any complication | 29 (5.302%) | 198 (6.310%) | 0.419 |
| BMI = body mass index; SD = standard deviation | | | |

### Baseline characteristics

#### Table S54: Surgical site infection – narrow definition

| Feature | No surgical site infection (n = 3623) | Surgical site infection, narrow definition (n = 62 (1.682%)) | P-value**** |
| --- | --- | --- | --- |
| Demographics | | | |
| Sex* (% female) | 63.649% | 62.903% | 1 |
| BMI* (mean (SD)) | 33.126 (6.555) | 33.738 (6.772) | 0.483 |
| Smoking current or former)* | 1164 (32.128%) | 21 (33.871%) | 0.877 |
| Low socioeconomic status (including Medicaid insurance and living in rural area)* | Pensioner card** = 1823 (50.317%)  SEIFA score*:  1 = 389 (10.737%)  2 = 243 (6.707%)  3 = 280 (7.728%)  4 = 302 (8.336%)  5 = 423 (11.675%)  6 = 279 (7.701%)  7 = 620 (17.113%)  8 = 393 (10.847%)  9 = 482 (13.304%)  10 = 212 (5.852%) | Pensioner card = 31 (50.000%)  SEIFA score:  1 = 4 (6.452%)  2 = 3 (4.839%)  3 = 4 (6.452%)  4 = 4 (6.452%)  5 = 7 (11.290%)  6 = 4 (6.452%)  7 = 14 (22.581%)  8 = 8 (12.903%)  9 = 9 (14.516%)  10 = 5 (8.065%) | 1  0.942 |
| Low socioeconomic status (including Medicaid insurance and living in rural area)* | Major cities of Australia = 2694 (77.201%)  Inner regional Australia = 681 (18.797%)  Outer regional or remote Australia = 131 (3.726%)  Missing = 10 (0.276%) | Major cities of Australia = 56 (90.323%)  Inner regional Australia = 6 (9.677%)  Outer regional or remote Australia = 0  Missing = 0 | 0.041 |
| Comorbidities | | | |
| Hypertension* | 2415 (66.657%) | 39 (62.903%) | 0.627 |
| Peripheral vascular disease* | 138 (3.588%) | 4 (6.452%) | 0.460 |
| Diabetes* | Diabetes = 809 (22.330%)  Diabetes with end-organ damage = 16 (0.442%) | Diabetes = 18 (29.032%)  Diabetes with end-organ damage = 0 | 0.407 |
| Rheumatoid arthritis* | 194 (5.355%) | 3 (6.290%) | 1 |
| Preoperative UTI* | 36 (0.994%) | 1 (1.613%) | 1 |
| Chronic skin condition* | 103 (2.843%) | 3 (4.834%) | 0.583 |
| Alcohol abuse* | 59 (1.628%) | 0 | 0.651 |
| ASA class* | 1 = 95 (2.622%)  2 = 1854 (51.173%)  3 = 1597 (44.079%)  4 = 54 (1.490%)  Missing = 23 (0.635%) | 1 = 1 (1.613%)  2 = 30 (48.387%)  3 = 28 (45.161%)  4 = 1 (1.613%)  Missing = 2 (3.226%) | 0.956 |
| Variables related to index hospital admission | | | |
| Duration of operation in minutes (mean (SD))** | 119.983 (34.726) | 113.436 (23.376) | 0.034 |
| Summer surgery** | 752 (20.756%) | 16 (25.806%) | 0.416 |
| Preoperative haemoglobin (mean (SD)) | 136.253 (13.838) | 133.265 (16.098) | 0.203 |
| Surgeon experience* | NA (de-identified proxy ID number for individual surgeons used) | NA (de-identified proxy ID number for individual surgeons used) |  |
| *Variable derived from Registry; **Variable derived from administrative database; ***”how much did pain interfere with your normal work?” – 1 = Not at all; 2 = A little bit; 3 = Moderately; 4 = Quite a bit; 5 = Extremely; **** Continuous variables were compared using Student’s t-test. Categorical variables were compared using chi-squared test, or Fisher’s exact test in cases of counts below 10; BMI = body mass index; SEIFA = Socioeconomic Indexes for Areas [19]; CHF = congestive heart failure; SES = socioeconomic status; IVDU = intravenous drug use; ED = emergency department | | | |

#### Table S55: Surgical site infection – broad definition

| Feature | No surgical site infection (n = 3601) | Surgical site infection, narrow definition (n = 84 (2.198%)) | P-value**** |
| --- | --- | --- | --- |
| Demographics | | | |
| Sex* (% female) | 63.677% | 61.905% | 0.827 |
| BMI* (mean (SD)) | 33.091 (6.537) | 35.093 (7.202) | 0.013 |
| Smoking current or former)* | 1155 (32.074%) | 30 (35.714%) | 0.557 |
| Low socioeconomic status (including Medicaid insurance and living in rural area)* | Pensioner card** = 1805 (50.125%)  SEIFA score*:  1 = 388 (10.775%)  2 = 241 (6.693%)  3 = 280 (7.776%)  4 = 300 (8.331%)  5 = 421 (11.691%)  6 = 276 (7.665%)  7 = 616 (17.106%)  8 = 391 (10.858%)  9 = 478 (13.274%)  10 = 210 (5.832%) | Pensioner card = 31 (58.333%)  SEIFA score:  1 = 5 (5.952%)  2 = 5 (5.952%)  3 = 4 (4.762%)  4 = 6 (7.143%)  5 = 9 (10.714%)  6 = 7 (8.333%)  7 = 18 (21.429%)  8 = 10 (11.905%)  9 = 13 (15.476%)  10 = 7 (8.333%) | 0.169  0.798 |
| Low socioeconomic status (including Medicaid insurance and living in rural area)* | Major cities of Australia = 2778 (77.145%)  Inner regional Australia = 679 (18.856%)  Outer regional or remote Australia = 134 (3.721%)  Missing = 10 (0.277%) | Major cities of Australia = 75 (89.286%)  Inner regional Australia = 8 (9.524%)  Outer regional or remote Australia = 1 (1.190%)  Missing = 0 | 0.033 |
| Comorbidities | | | |
| Hypertension* | 2402 (66.704%) | 52 (61.905%) | 0.421 |
| Peripheral vascular disease* | 137 (3.804%) | 5 (5.952%) | 0.469 |
| Diabetes* | Diabetes = 802 (22.272%)  Diabetes with end-organ damage = 16 (0.444%) | Diabetes = 25 (29.762%)  Diabetes with end-organ damage = 0 | 0.239 |
| Rheumatoid arthritis* | 194 (5.332%) | 5 (5.952%) | 0.996 |
| Preoperative UTI* | 35 (0.972%) | 1 (2.381%) | 0.467 |
| Chronic skin condition* | 103 (2.833%) | 3 (4.762%) | 0.474 |
| Alcohol abuse* | 59 (1.638%) | 0 | 0.458 |
| ASA class* | 1 = 95 (2.638%)  2 = 1849 (51.347%)  3 = 1581 (43.904%)  4 = 53 (1.472%)  Missing = 23 (0.639%) | 1 = 1 (1.190%)  2 = 35 (41.667%)  3 = 44 (52.381%)  4 = 2 (2.381%)  Missing = 2 (2.381%) | 0.262 |
| Variables related to index hospital admission | | | |
| Duration of operation in minutes (mean (SD))** | 119.890 (34.460) | 119.155 (39.517) | 0.866 |
| Summer surgery** | 749 (20.800%) | 19 (22.619%) | 0.787 |
| Preoperative haemoglobin (mean (SD)) | 136.231 (13.851) | 135.143 (15.051) | 0.551 |
| Surgeon experience* | NA (de-identified proxy ID number for individual surgeons used) | NA (de-identified proxy ID number for individual surgeons used) |  |
| *Variable derived from Registry; **Variable derived from administrative database; ***”how much did pain interfere with your normal work?” – 1 = Not at all; 2 = A little bit; 3 = Moderately; 4 = Quite a bit; 5 = Extremely; **** Continuous variables were compared using Student’s t-test. Categorical variables were compared using chi-squared test, or Fisher’s exact test in cases of counts below 10; BMI = body mass index; SEIFA = Socioeconomic Indexes for Areas [19]; CHF = congestive heart failure; SES = socioeconomic status; IVDU = intravenous drug use; ED = emergency department | | | |

#### Table S56: Venous thromboembolism – narrow definition

| Feature | No VTE, narrow definition (n = 3644) | VTE, narrow definition (n = 41 (1.113%)) | P-value**** |
| --- | --- | --- | --- |
| Demographics | | | |
| Age* (mean (SD)) | 69.569 (8.839) | 69.366 (8.946) | 0.886 |
| Sex* (% female) | 63.557% | 70.732% | 0.432 |
| BMI* (mean (SD)) | 33.141 (6.562) | 32.736 (6.342) | 0.687 |
| Comorbidities | | | |
| Thromboprophylaxis (out of hospital)* | 107 (2.936%) | 1 (2.439%) | 1 |
| CHF* | 107 (2.936%) | 3 (7.317%) | 0.122 |
| History of cancer* | 342 (9.805%) | 29 (14.721%) | 0.035 |
| Factor V Leiden* | 5 (0.137%) | 1 (2.439%) | 0.065 |
| Prior VTE* | 239 (6.559%) | 9 (21.951%) | 0.001 |
| Past AMI* | 166 (4.555%) | 0 | 0.260 |
| Pulmonary disease* | 192 (5.269%) | 5 (12.195%) | 0.107 |
| Rheumatoid arthritis* | 194 (5.324%) | 3 (7.317%) | 0.830 |
| Haematologic disorder* | 36 (0.988%) | 0 | 1 |
| Varicose veins* | 29 (0. 796%) | 0 | <0.001 |
| Inflammatory bowel disease* | 23 (0. 631%) | 0 | 1 |
| Polio* | 11 (0.302%) | 0 | 1 |
| Variables related to index hospital admission | | | |
| Cemented prosthesis* | 1795 (49.259%) | 16 (39.024%) | 0.252 |
| Mechanical thromboprophylaxis | 1617 (44.374%) | 12 (29.268%) | 0.075 |
| Pharmacological thromboprophylaxis | 1614 (44.300%) | 12 (29.268%) | 0.077 |
| Preoperative haemoglobin (mean (SD)) | 136.224 (13.909) | 134.757 (10.986) | 0.426 |
| *Variable derived from Registry; **Variable derived from administrative database; ***”how much did pain interfere with your normal work?” – 1 = Not at all; 2 = A little bit; 3 = Moderately; 4 = Quite a bit; 5 = Extremely; **** Continuous variables were compared using Student’s t-test. Categorical variables were compared using chi-squared test, or Fisher’s exact test in cases of counts below 10; BMI = body mass index; SEIFA = Socioeconomic Indexes for Areas [19]; CHF = congestive heart failure; SES = socioeconomic status; IVDU = intravenous drug use; ED = emergency department | | | |

#### Table S57: Joint-specific complication – narrow definition

| Feature | No complication (n = 3532) | Complication (n = 153 (4.152%)) | P-value**** |
| --- | --- | --- | --- |
| Demographics | | | |
| Age* (mean (SD)) | 69.320 (8.452) | 69.578 (8.856) | 0.713 |
| Sex* (% female) | 63.732% | 61.438% | 0.623 |
| BMI* (mean (SD)) | 33.067 (6.515) | 34.736 (7.334) | 0.006 |
| Low SES | Pensioner card** = 1766 (50.000%) | Pensioner card = 88 (57.516%) | 0.082 |
| Comorbidities | | | |
| Diabetes* | Diabetes = 791 (22.395%)  Diabetes with end-organ damage = 16 (0. 453%) | Diabetes = 36 (23.529%)  Diabetes with end-organ damage = 0 | 0.882 |
| Coagulopathy* | 19 (0.538%) | 0 | 0.624 |
| CHF* | 101 (2.860%) | 9 (5.882%) | 0.300 |
| Liver disease* | 89 (2.520%) | 4 (2.614%) | 0.794 |
| Depression* | 407 (11.523%) | 23 (15.033%) | 0.232 |
| CKD* | 135 (3.822%) | 6 (3.921%) | 0.831 |
| Chronic pulmonary disease* | 184 (5.210%) | 13 (8.497%) | 0.113 |
| Pulmonary circulation disorder* | 9 (0.255%) | 0 (0.461%) | 1 |
| Lymphoma* | 37 (1.048%) | 1 (0.654%) | 1 |
| Other neurological disorder* | 330 (9.343%) | 15 (9.804%) | 0.960 |
| Variables related to index hospital admission | | | |
| In-hospital complication (any) during index admission* | 518 (14.666%) | 61 (22.876%) | 0.008 |
| Bilateral TKA | 35 (0.991%) | 2 (1.307%) | 0.665 |
| *Variable derived from Registry; **Variable derived from administrative database; ***”how much did pain interfere with your normal work?” – 1 = Not at all; 2 = A little bit; 3 = Moderately; 4 = Quite a bit; 5 = Extremely; **** Continuous variables were compared using Student’s t-test. Categorical variables were compared using chi-squared test, or Fisher’s exact test in cases of counts below 10; BMI = body mass index; SEIFA = Socioeconomic Indexes for Areas [19]; CHF = congestive heart failure; SES = socioeconomic status; IVDU = intravenous drug use; ED = emergency department | | | |

#### Table S58: Joint-specific complication – broad definition

| Feature | No complication (n = 3468) | Complication (n = 217 (5.889%)) | P-value**** |
| --- | --- | --- | --- |
| Demographics | | | |
| Age* (mean (SD)) | 69.945 (8.679) | 69.544 (8.850) | 0.510 |
| Sex* (% female) | 63.783% | 38.710% | 0.504 |
| BMI* (mean (SD)) | 33.068 (6.519) | 34.233 (7.082) | 0.019 |
| Low SES | Pensioner card** = 1723 (49.683%) | Pensioner card = 131 (60.369%) | 0.003 |
| Comorbidities | | | |
| Diabetes* | Diabetes = 769 (22.174%)  Diabetes with end-organ damage = 16 (0.461%) | Diabetes = 58 (26.728%)  Diabetes with end-organ damage = 0 | 0.227 |
| Coagulopathy* | 19 (0.548%) | 0 | 0.624 |
| CHF* | 101 (2.912%) | 9 (4.147%) | 0.300 |
| Liver disease* | 89 (2.520%) | 6 (2.765%) | 0.822 |
| Depression* | 399 (11505%) | 31 (14.286%) | 0.259 |
| CKD* | 128 (3.691%) | 13 (5.991%) | 0.126 |
| Chronic pulmonary disease* | 178 (5.133%) | 19 (8.756%) | 0.032 |
| Pulmonary circulation disorder* | 8 (0.231%) | 1 (0.461%) | 0.421 |
| Lymphoma* | 35 (1.009%) | 3 (1.382%) | 0.488 |
| Other neurological disorder* | 318 (9.170%) | 27 (12.442%) | 0.137 |
| Variables related to index hospital admission | | | |
| In-hospital complication (any) during index admission* | 518 (14.187%) | 35 (28.111%) | <0.0001 |
| Bilateral TKA | 35 (0.922%) | 2 (0.922%) | 1 |
| *Variable derived from Registry; **Variable derived from administrative database; ***”how much did pain interfere with your normal work?” – 1 = Not at all; 2 = A little bit; 3 = Moderately; 4 = Quite a bit; 5 = Extremely; **** Continuous variables were compared using Student’s t-test. Categorical variables were compared using chi-squared test, or Fisher’s exact test in cases of counts below 10; BMI = body mass index; SEIFA = Socioeconomic Indexes for Areas [19]; CHF = congestive heart failure; SES = socioeconomic status; IVDU = intravenous drug use; ED = emergency department | | | |

#### Table S59: Combined outcome

| Feature | No complication (n = 3458) | Complication (n = 227 (6.160%)) | P-value**** |
| --- | --- | --- | --- |
| Demographics | | | |
| Age* (mean (SD)) | 70.198 (8.803) | 69.526 (8.841) | 0.266 |
| Sex* (% female) | 63.852% | 60.352% | 0.322 |
| BMI* (mean (SD)) | 33.081 (6.518) | 33.983 (7.116) | 0.064 |
| Smoking* | 271 (7.837%) | 19 (8.370%) | 0.872 |
| Low SES | Pensioner card** = 1717 (49.653%)  SEIFA score*:  1 = 378 (10.931%)  2 = 231 (6.680%)  3 = 273 (7.895%)  4 = 289 (8.357%)  5 = 409 (11.828%)  6 = 264 (7.634%)  7 = 594 (17.178%)  8 = 373 (10.787%)  9 = 453 (13.100%)  10 = 194 (5.610%) | Pensioner card = 137 (60.352%)  SEIFA score:  1 = 15 (6.608%)  2 = 15 (6.608%)  3 = 11 (4.846%)  4 = 17 (7.489%)  5 = 21 (9.251%)  6 = 19 (8.370%)  7 = 40 (17.621%)  8 = 28 (12.335%)  9 = 38 (16.740%)  10 = 23 (10.132%) | 0.002  0.041 |
| Poor access to post-op care: lives far from hospital, lack of access to allied health support, lack of access to telehealth support* | Major cities of Australia = 2662 (76.981%)  Inner regional Australia = 655 (18.942%)  Outer regional or remote Australia = 132 (3.817%)  Missing = 9 (0.260%) | Major cities of Australia = 191 (84.141%)  Inner regional Australia = 32 (14.100%)  Outer regional or remote Australia = 4 (1.322%)  Missing = 1 (0.441%) | 0.018 |
| Patient-related biopsychosocial: lower education level, poor health literacy, non-English speaking | Interpreter required* = 571 (16.512%)  Missing = 31 (0.896%) | Interpreter required = 33 (14.537%)  Missing = 5 (2.203%) | 0.545 |
| Comorbidities | | | |
| Hypertension* | 2307 (66.715%) | 147 (64.758%) | 0.594 |
| Peripheral vascular disease* | 130 (3.759%) | 12 (5.286%) | 0.327 |
| Diabetes* | Diabetes = 766 (22.152%)  Diabetes with end-organ damage = 16 (0.463%) | Diabetes = 55 (26.872%)  Diabetes with end-organ damage = 0 | 0.199 |
| Coagulopathy* | 19 (0.549%) | 0 | 0.627 |
| Charlson Comorbidity Index* | Zero = 1785 (51.619%)  One = 967 (27.964%)  ≥Two = 706 (20.416%) | Zero = 108 (47.577%)  One = 59 (25.991%)  ≥Two = 60 (26.432%) | 0.096 |
| CHF* | 101 (2.921%) | 9 (3.965%) | 0.319 |
| Liver disease* | 87 (2.516%) | 6 (2.643%) | 0.827 |
| Depression* | 396 (11.452%) | 34 (14.978%) | 0.135 |
| Previous stroke* | 211 (8.370%) | 19 (8.370%) | 0.220 |
| Anaemia* | 60 (1.735%) | 8 (3.524%) | 0.068 |
| History of cancer* | 342 (9.900%) | 29 (12.775%) | 0.199 |
| High risk of infection: High risk of infectiond state, active IVDU, infection in other primary joint replacement* | 1480 (42.799%) | 112 (49.339%) | 0.063 |
| CKD* | 127 (3.673%) | 15 (6.608%) | 0.032 |
| Arrhythmia* | 18 (0.521%) | 0 | 0.623 |
| Pulmonary disease* | 178 (5.147%) | 12 (8.370%) | 0.053 |
| Dementia* | 12 (0.347%) | 0 | 1 |
| Substance abuse* | 59 (1.706%) | 3 (1.322%) | 1 |
| ASA class* | 1 = 94 (2.718%)  2 = 1780 (51.475%)  3 = 1512 (43.725%)  4 = 50 (1.446%)  Missing = 22 (0.636%) | 1 = 2 (0.881%)  2 = 104 (45.815%)  3 = 113 (49.780%)  4 = 5 (2.203%)  Missing = 3 (1.322%) | 0.068 |
| Rheumatoid arthritis | 184 (5.321%) | 13 (5.727%) | 0.912 |
| Preoperative UTI | 34 (0.983%) | 3 (1.322%) | 0.495 |
| Chronic skin condition | 99 (2.863%) | 7 (3.084%) | 0.836 |
| Alcohol abuse | 56 (1.619%) | 3 (1.322%) | 1 |
| Prior VTE | 219 (6.333%) | 29 (12.775%) | <0.001 |
| Factor V Leiden | 3 (0.087%) | 3 (1.322%) | 0.004 |
| Haematologic disorder | 34 (0.983%) | 3 (0.881%) | 1 |
| Psychoses | 19 (0.549%) | 3 (1.322%) | 0.150 |
| Thromboprophylaxis (out of hospital) | 93 (2.689%) | 15 (6.608%) | 0.001 |
| Varicose veins | 29 (0.839%) | 0 | <0.001 |
| Inflammatory bowel disease | 21 (0.607%) | 2 (0.881%) | 0.650 |
| Polio | 10 (0.289%) | 1 (0.441%) | 0.504 |
| Pulmonary circulation disorder | 8 (0.231%) | 1 (0.441%) | 0.436 |
| Other neurological disorder | 317 (9.167%) | 28 (12.335%) | 0.142 |
| Lymphoma | 35 (1.012%) | 28 (1.322%) | 0.506 |
| Peptic ulcer disease | 103 (2.979%) | 9 (3.965%) | 0.420 |
| Any cardiac comorbidity | 830 (24.002%) | 55 (24.229%) | 1 |
| Patient-reported variables | | | |
| Preoperative patient-reported level of function (mean (SD))* | Mental function = 44.365 (15.096)  Physical function = 24.701 (7.829) | Mental function = 42.449 (16.699)  Physical function = 23.620 (7.356) | 0.114  0.047 |
| Preoperative patient-reported pain level*  *** | One = 22 (0.636%)  Two = 127 (3.673%)  Three = 455 (13.158%)  Four = 1386 (40.081%) Five = 966 (27.935%)  Missing = 502 (14.517%) | One = 1 (0.441%)  Two = 4 (1.762%) Three = 21 (9.251%) Four = 95 (41.850%) Five = 78 (34.361%) Missing = 28 (12.335%) | 0.097 |
| Prior healthcare utilisation | | | |
| Increasing number of previous admissions** | Zero = 3245 (93.836%)  One = 116 (3.355%)  Two = 51 (1.475%) ≥Three = 46 (1.330%) | Zero = 208 (91.630%)  One = 10 (4.405%)  Two = 3 (1.322%) ≥Three = 6 (2.643%) | 0.291 |
| Number of prior ED presentations (12 months)** | Zero = 3320 (96.009%)  One = 82 (2.371%)  ≥Two = 56 (1.619%) | Zero = 212 (93.392%)  One = 10 (4.405%)  ≥Two = 5 (2.203%) | 0.117 |
| Historical knee procedures** | Zero = 1237 (35.772%)  One = 1338 (38.519%)  Two = 767 (22.180%)  ≥Three = 122 (3.528%) | Zero = 79 (34.802%)  One = 69 (30.396%)  Two = 44 (19.383%)  ≥Three = 35 (15.419%) | <0.001 |
| Variables related to index hospital admission | | | |
| In-hospital complication (any) during index admission* | 487 (14.864%) | 66 (29.075%) | <0.001 |
| ICU/HDU admission during index admission** | Zero = 3325 (96.154%)  One = 64 (1.851%) ≥Two = 69 (1.995%) | Zero = 215 (94.714%)  One = 4 (1.762%) Two = 8 (3.524%) | 0.294 |
| Return to theatre during index admission** | 10 (0.289%) | 6 (3.046%) | <0.001 |
| Length of stay in days (mean (SD))* | 9.000 (4.442) | 11.419 (8.700) | <0.001 |
| Duration of operation in minutes (mean (SD))** | 119.915 (34.487) | 119.229 (35.939) | 0.780 |
| Wound class (not clean)** | 7 (0.202%) | 0 | 1 |
| Transfusion during surgery in number of packed red blood cells (mean (SD))* | Zero = 3130 (90.515%)  One = 65 (1.880%)  Two = 208 (6.015%)  ≥Three = 55 (1.591%) | Zero = 192 (84.582%)  One = 3 (1.322%)  Two = 19 (8.370%)  ≥Three = 13 (5.727%) | 0.001 |
| Preoperative haemoglobin (mean (SD)) | 136.378 (13.847) | 134.757 (14.244) | 0.067 |
| Surgeon experience* | NA (de-identified proxy ID number for individual surgeons used) | NA (de-identified proxy ID number for individual surgeons used) |  |
| DVT prophylaxis other than warfarin | 1544 (44.650%) | 82 (36.123%) | 0.015 |
| Mechanical thromboprophylaxis | 1546 (44.708%) | 83 (36.564%) | 0.020 |
| Pharmacological thromboprophylaxis | 1544 (44.650%) | 82 (36.123%) | 0.015 |
| Anaesthetic - general | 591 (17.091%) | 45 (19.824%) | 0.335 |
| Anaesthetic - epidural | 36 (1.041%) | 0 | 0.167 |
| Anaesthetic - local | 20 (0.578%) | 1 (0.441%) | 1 |
| Anaesthetic - spinal | 2913 (84.239%) | 187 (82.379%) | 0.516 |
| Anaesthetic - sedation | 1062 (30.711%) | 54 (23.789%) | 0.034 |
| Cemented prosthesis | 1703 (49.248%) | 108 (47.577%) | 0.675 |
| Simultaneous bilateral TKA | 35 (1.012%) | 2 (0.881%) | 1 |
| Summer surgery | 719 (20.792%) | 49 (21.586%) | 0.841 |
| *Variable derived from SMART registry; **Variable derived from administrative database; ***”how much did pain interfere with your normal work?” – 1 = Not at all; 2 = A little bit; 3 = Moderately; 4 = Quite a bit; 5 = Extremely; **** Continuous variables were compared using Student’s t-test. Categorical variables were compared using chi-squared test, or Fisher’s exact test in cases of counts below 10; BMI = body mass index; SEIFA = Socioeconomic Indexes for Areas [19]; CHF = congestive heart failure; SES = socioeconomic status; IVDU = intravenous drug use; ED = emergency department | | | |

### Table S60: Cause-specific models developed

| **Model*** | **Training set AUC** |
| --- | --- |
| **Surgical site infection** | |
| SSI narrow definition, combined dataset, discharge, logistic regression (LASSO) | 0.575 |
| SSI narrow definition, combined dataset, discharge, random forest (Top k (k = 62/10 = 6)) | 0.515 |
| SSI narrow definition, combined dataset, initial consultation, logistic regression (LASSO) | 0.500 |
| SSI narrow definition, combined dataset, initial consultation, random forest (Top k (k = 62/10 = 6)) | 0.492 |
| SSI narrow definition, administrative database dataset, discharge, logistic regression | 0.519 |
| SSI narrow definition, administrative database dataset, discharge, random forest | 0.501 |
| SSI narrow definition, administrative database dataset, initial consultation, logistic regression | 0.506 |
| SSI narrow definition, administrative database dataset, initial consultation, random forest | 0.485 |
| SSI broad definition, combined dataset, discharge, logistic regression (LASSO) | 0.604 = highest performance for SSI |
| SSI broad definition, combined dataset, discharge, random forest (Top k (k = 84/10 = 8)) | 0.572 |
| SSI broad definition, combined dataset, initial consultation, logistic regression (LASSO) | 0.534 |
| SSI broad definition, combined dataset, initial consultation, random forest (Top k (k = 84/10 = 8)) | 0.530 |
| SSI broad definition, administrative database dataset, discharge, logistic regression | 0.505 |
| SSI broad definition, administrative database dataset, discharge, random forest | 0.489 |
| SSI broad definition, administrative database dataset, initial consultation, logistic regression | 0.548 = best of the two initial consultation administrative dataset models |
| SSI broad definition, administrative database dataset, initial consultation, random forest | 0.503 |
| **Joint-specific complications** | |
| Joint-specific narrow definition, combined dataset, discharge, logistic regression (LASSO) | 0.544 |
| Joint-specific narrow definition, combined dataset, discharge, random forest (Top k (k = 153/10 = 15)) | 0.536 |
| Joint-specific narrow definition, combined dataset, initial consultation, logistic regression | 0.550 |
| Joint-specific narrow definition, combined dataset, initial consultation, random forest | 0.537 |
| Joint-specific narrow definition, administrative database dataset, discharge, logistic regression | 0.530 |
| Joint-specific narrow definition, administrative database dataset, discharge, random forest | 0.538 |
| Joint-specific narrow definition, administrative database dataset, initial consultation, logistic regression | 0.533 |
| Joint-specific narrow definition, administrative database dataset, initial consultation, random forest | 0.532 |
| Joint-specific broad definition, combined dataset, discharge, logistic regression | 0.565 |
| Joint-specific broad definition, combined dataset, discharge, random forest | 0.585 = highest performance for joint-specific complications |
| Joint-specific broad definition, combined dataset, initial consultation, logistic regression | 0.546 |
| Joint-specific broad definition, combined dataset, initial consultation, random forest | 0.558 |
| Joint-specific broad definition, administrative database dataset, discharge, logistic regression | 0.549 |
| Joint-specific broad definition, administrative database dataset, discharge, random forest | 0.540 |
| Joint-specific broad definition, administrative database dataset, initial consultation, logistic regression | 0.556 = best of the two initial consultation administrative dataset models |
| Joint-specific broad definition, administrative database dataset, initial consultation, random forest | 0.548 |
| **VTE** | |
| VTE narrow definition, combined dataset, discharge, logistic regression (LASSO) | 0.616 = highest performance for VTE |
| VTE narrow definition, combined dataset, discharge, random forest (Top k (k = 41/10 = 4)) | 0.522 |
| VTE narrow definition, combined dataset, initial consultation, logistic regression (LASSO) | 0.550 |
| VTE narrow definition, combined dataset, initial consultation, random forest (Top k (41/10 = 4)) | 0.573 |
| VTE narrow definition, administrative database dataset, initial consultation, logistic regression | 0.531 |
| VTE narrow definition, administrative database dataset, initial consultation, random forest | 0.549 = best of the two initial consultation administrative dataset models |
| **Combined outcome – any complication associated with readmission** | |
| Combined outcome (any readmission-causing complication), combined dataset, discharge, logistic regression (LASSO) | 0.603 |
| Combined outcome (any readmission-causing complication), combined dataset, discharge, random forest (Top k (227/10 = 22)) | 0.632 = highest performance for combined outcome |
| Combined outcome (any readmission-causing complication), combined dataset, initial consultation, logistic regression (LASSO) | 0.569 |
| Combined outcome (any readmission-causing complication), combined dataset, initial consultation, random forest (Top k (227/10 = 22)) | 0.608 |
| Combined outcome (any readmission-causing complication), administrative database dataset, discharge, logistic regression | 0.542 |
| Combined outcome (any readmission-causing complication), administrative database dataset, discharge, random forest | 0.506 |
| Combined outcome (any readmission-causing complication), administrative database dataset, initial consultation, logistic regression | 0.566 = best of the two initial consultation administrative dataset models |
| Combined outcome (any readmission-causing complication), administrative database dataset, initial consultation, random forest | 0.541 |
| **All study predictors - Combined outcome – any complication associated with readmission** | |
| All study predictors - Combined outcome (any readmission-causing complication), combined dataset, discharge, logistic regression (LASSO) | 0.665 |
| All study predictors - Combined outcome (any readmission-causing complication), combined dataset, discharge, random forest (Top k (227/10 = 22)) | 0.700 = best overall |
| All study predictors - Combined outcome (any readmission-causing complication), combined dataset, initial consultation, logistic regression (LASSO) | 0.599 |
| All study predictors - Combined outcome (any readmission-causing complication), combined dataset, initial consultation, random forest (Top k (227/10 = 22)) | 0.646 |
| All study predictors - Combined outcome (any readmission-causing complication), administrative database dataset, discharge, logistic regression | 0.607 |
| All study predictors - Combined outcome (any readmission-causing complication), administrative database dataset, discharge, random forest | 0.643 |
| All study predictors - Combined outcome (any readmission-causing complication), administrative database dataset, initial consultation, logistic regression | 0.565 = best of the two initial consultation administrative dataset models |
| All study predictors - Combined outcome (any readmission-causing complication), administrative database dataset, initial consultation, random forest | 0.556 |
| *There were not enough events in our dataset to develop models to predict gastrointestinal, cardiac, or non-surgical site infection complications. However, we developed a combined outcome variable indicating the occurrence of any of complications commonly associated with readmission. Two predictor selection strategies were used. First, models were developed to predict the combined outcome based on all the predictors used for these complication-specific models. Second, models were developed to predict the combined outcome using all the predictors generated in this study, including those generated for the readmission prediction models. | |

### Full model evaluation

#### SSI full model evaluation - best overall (SSI broad definition, combined dataset, discharge, logistic regression (LASSO)) – Figures S46-S48, Table S61, Figure S49, Table S62

##### Figure S46: Training set ROC curve


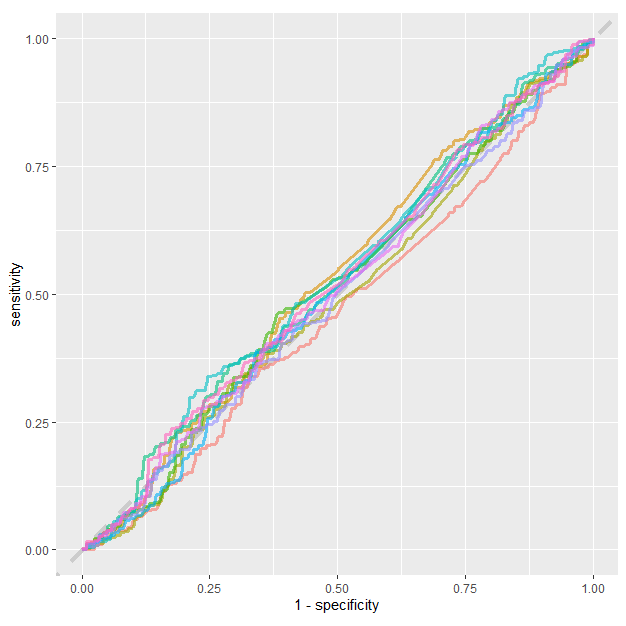


##### Figure S47: Test ROC curve


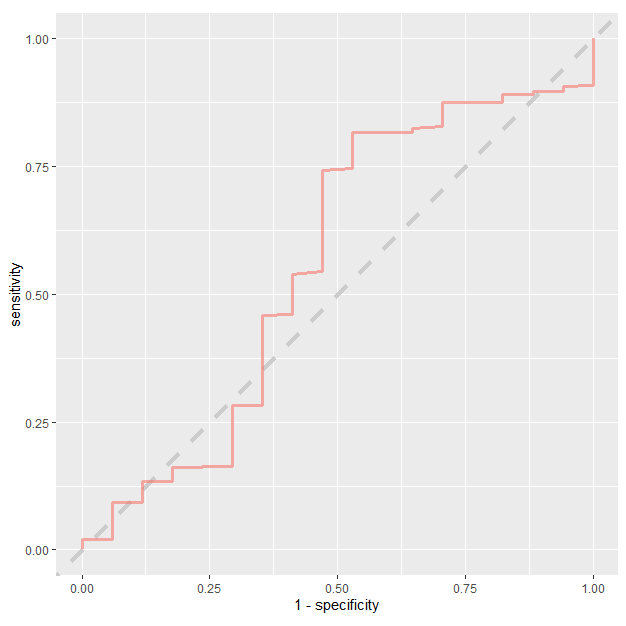


##### Figure S48: Precision-recall (PPV-sensitivity) curve


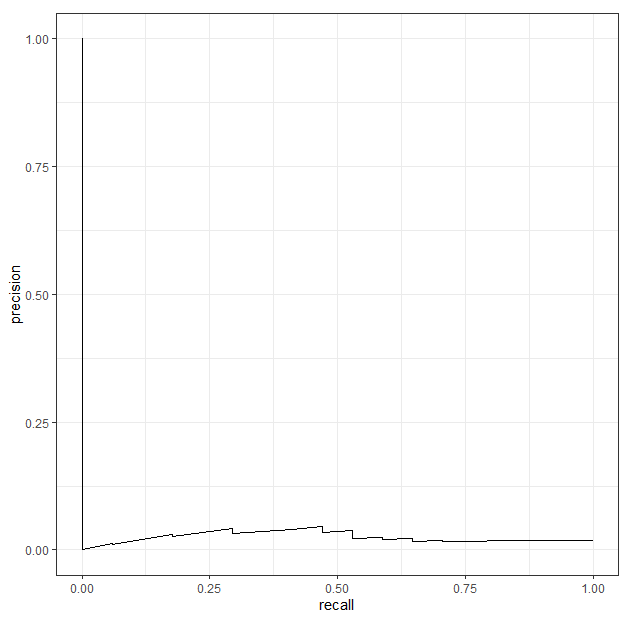


##### Table S61: Performance metrics

| **Thresholds** | **Performance metrics** |
| --- | --- |
| Optimal threshold to maximise MCC = 0.027 | True positives = 8  True negatives = 738  False negatives = 9  False positives = 166  MCC = 0.099  Sensitivity (recall) = 0.471  Specificity = 0.816  Youden index = 0.287  F statistic = 0.084  PPV (precision) = 0.046  NPV = 0.990 |
| Threshold-free metrics | Discrimination:  AUC ROC (training set) = 0.604  AUC ROC (test set) = 0.559 (0.396-0.723)  AUC PR = 0.024  Calibration:  Slope = -0282 (-2.767 to 2.202)  Intercept = -0.265 (-0.745 to 0.215)  ICI = 0.011  E50 = 0.007  E90 = 0.020  Emax = 0.048 |
| MCC = Matthews Correlation Coefficient; PPV = positive predictive value; NPV = negative predictive value; AUC ROC = Area under the receiver operating characteristic curve; AUC PR = Area under the precision-recall (PPV-sensitivity) curve; ICI = Integrated calibration index; E50 = median of the absolute difference between observed and predicted probabilities; E90 = 90th percentile of the absolute difference between observed and predicted probabilities; Emax = maximal absolute difference between observed and predicted probabilities of the outcome | |

##### Figure S49: Calibration curve


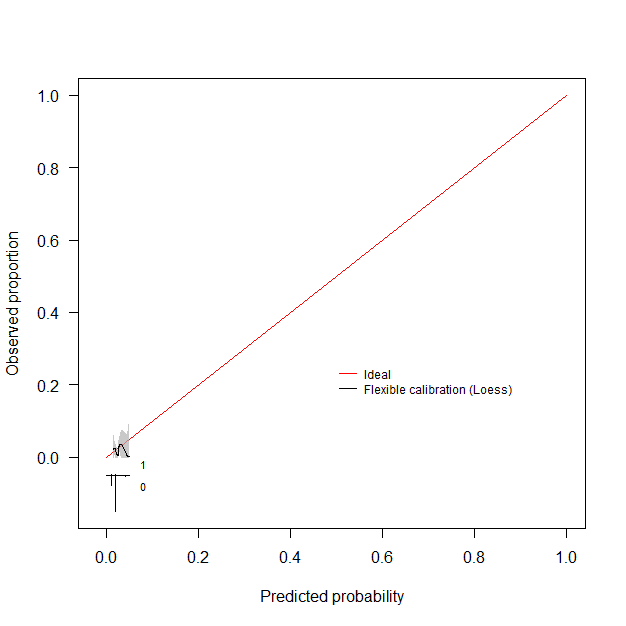


##### Table S62: Model coefficients

| Variable | Coefficient |
| --- | --- |
| (Intercept) | -3.720 |
| BMI | 0.070 |
| Preoperative haemoglobin | 0 |
| Duration of operation | 0 |
| ASA class (two) | 0 |
| ASA class (three) | 0 |
| ASA class (four) | 0 |
| Sex | 0 |
| Diabetes without end-organ damage | 0 |
| Diabetes with end-organ damage | 0 |
| Rheumatoid arthritis | 0 |
| Preoperative urinary tract infection | 0 |
| Peripheral vascular disease | 0 |
| Chronic skin condition | 0 |
| Smoker or former smoker | 0 |
| Alcohol abuse | 0 |
| Surgeon ID X (assigned ID number removed for blinding purposes) | 0 |
| Surgeon ID X (assigned ID number removed for blinding purposes) | 0.116 |
| Surgeon ID X (assigned ID number removed for blinding purposes) | 0 |
| Surgeon ID X (assigned ID number removed for blinding purposes) | 0 |
| Surgeon ID X (assigned ID number removed for blinding purposes) | 0 |
| Surgeon ID X (assigned ID number removed for blinding purposes) | 0 |
| Surgeon ID X (assigned ID number removed for blinding purposes) | 0 |
| Surgeon ID X (assigned ID number removed for blinding purposes) | 0 |
| Surgeon ID X (assigned ID number removed for blinding purposes) | 0 |
| Surgeon ID X (assigned ID number removed for blinding purposes) | -0.155 |
| Surgeon ID X (assigned ID number removed for blinding purposes) | 0 |
| Surgeon ID X (assigned ID number removed for blinding purposes) | 0 |
| Surgeon ID X (assigned ID number removed for blinding purposes) | 0 |
| Surgeon ID X (assigned ID number removed for blinding purposes) | 0 |
| Surgeon ID X (assigned ID number removed for blinding purposes) | 0 |
| Surgeon ID X (assigned ID number removed for blinding purposes) | 0 |
| Surgeon ID X (assigned ID number removed for blinding purposes) | 0 |
| Surgeon ID X (assigned ID number removed for blinding purposes) | 0 |
| Surgeon ID X (assigned ID number removed for blinding purposes) | 0 |
| Surgeon ID X (assigned ID number removed for blinding purposes) | 0 |
| Surgeon ID X (assigned ID number removed for blinding purposes) | 0 |
| Surgeon ID X (assigned ID number removed for blinding purposes) | 0 |
| Surgeon ID X (assigned ID number removed for blinding purposes) | 0 |
| Surgeon ID X (assigned ID number removed for blinding purposes) | 0 |
| Surgeon ID X (assigned ID number removed for blinding purposes) | 0 |
| SEIFA score (2) | 0 |
| SEIFA score (3) | 0 |
| SEIFA score (4) | 0 |
| SEIFA score (5) | 0 |
| SEIFA score (6) | 0 |
| SEIFA score (7) | 0 |
| SEIFA score (8) | 0 |
| SEIFA score (9) | 0 |
| SEIFA score (10) | 0 |
| Remoteness Area (Major cities of Australia) | 0 |
| Remoteness Area (Outer regional or remote Australia) | 0 |
| Pensioner Card | 0 |
| Summer surgery | 0 |
| Hypertension | 0 |

#### SSI full model evaluation - best administrative database – initial consultation, logistic regression (Figures S50-S52, Table S63, Figure S53, Table S64)

##### Figure S50: Training set ROC curve


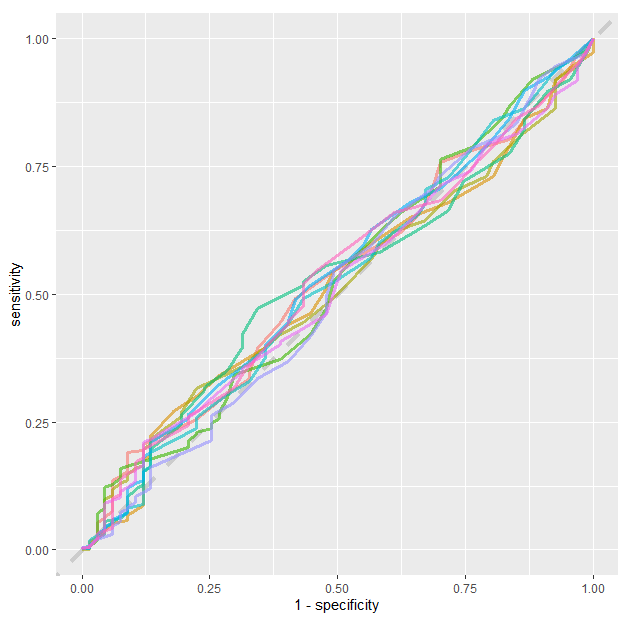


Figure S51: Test ROC curve
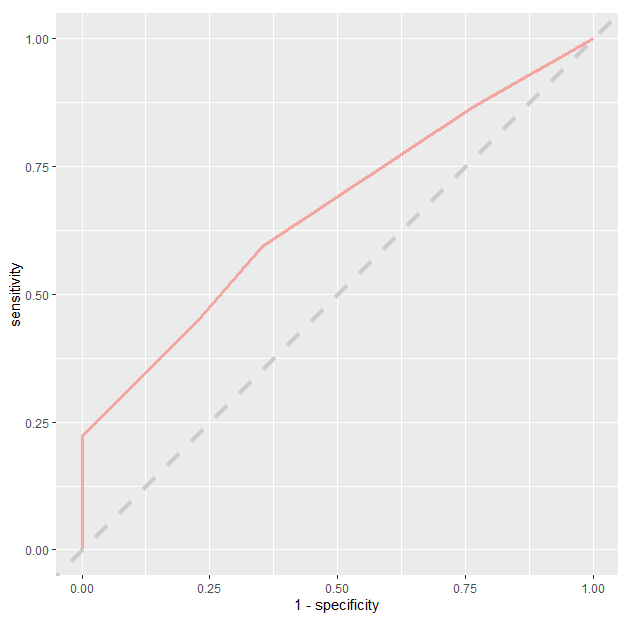


##### Figure S52: Precision-recall (PPV-sensitivity) curve


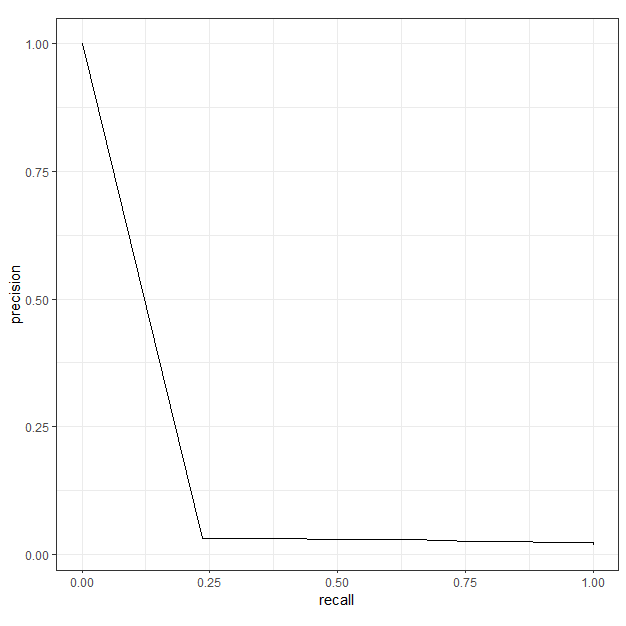


##### Table S63: Performance metrics

| **Thresholds** | **Performance metrics** |
| --- | --- |
| Optimal threshold to maximise MCC = 0.022 | True positives = 17  True negatives = 201  False negatives = 0  False positives = 703  MCC = 0.072  Sensitivity (recall) = 1  Specificity = 0.222  Youden index = 0.222  F statistic = 0.046  PPV (precision) = 0.024  NPV = 1 |
| Threshold-free metrics | Discrimination:  AUC ROC (training set) = 0.548  AUC ROC (test set) = 0.662 (0.556-0.769)  AUC PR = 0.143  Calibration:  Slope = 2.886 (0.373 to 5.399)  Intercept = -0.284 (-0.764 to 0.196)  ICI = 0.006  E50 = 0.006  E90 = 0.013  Emax = 0.014 |
| MCC = Matthews Correlation Coefficient; PPV = positive predictive value; NPV = negative predictive value; AUC ROC = Area under the receiver operating characteristic curve; AUC PR = Area under the precision-recall (PPV-sensitivity) curve; ICI = Integrated calibration index; E50 = median of the absolute difference between observed and predicted probabilities; E90 = 90th percentile of the absolute difference between observed and predicted probabilities; Emax = maximal absolute difference between observed and predicted probabilities of the outcome | |

##### Figure S53: Calibration curve


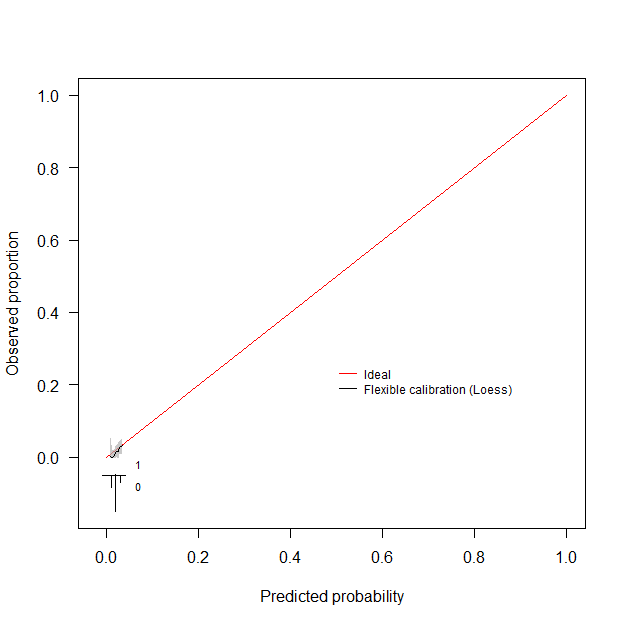


##### Table S64: Coefficients

| (Intercept) | 0.024 |
| --- | --- |
| Sex | 1.080 |
| Remoteness area (major cities of Australia) | 1.280 |
| Remoteness area (outer regional or remote Australia) | 0.945 |
| Pensioner concession card | 1.14 |

#### VTE full model evaluation - best overall; combined dataset, discharge, logistic regression (LASSO) – Figures S54-S56, Table S65, Figure S57, Table S66)

##### Figure S54: Training set ROC curve


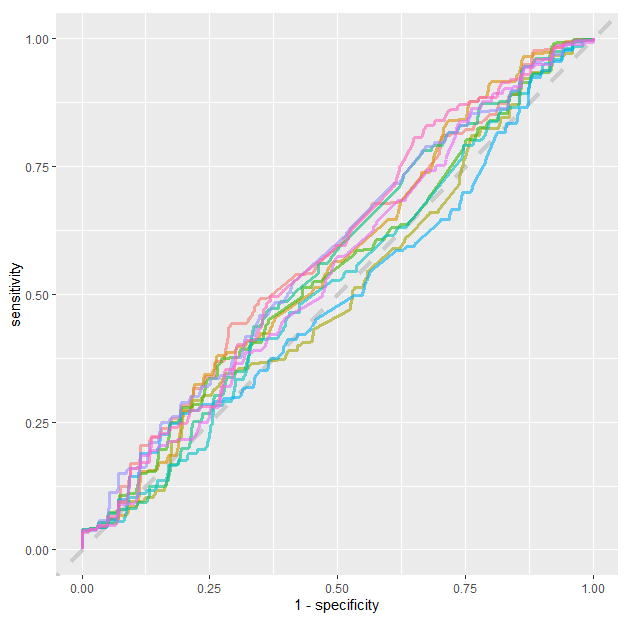


##### Figure S55: Test ROC curve


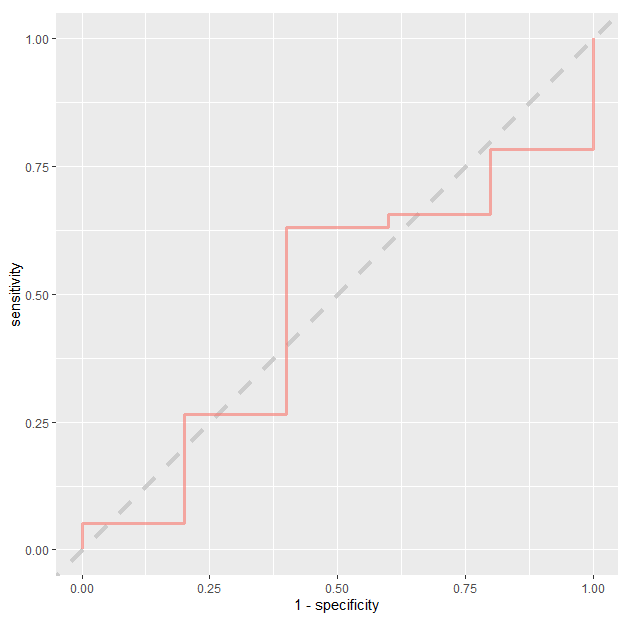


##### Figure S56: Precision-recall (PPV-sensitivity) curve


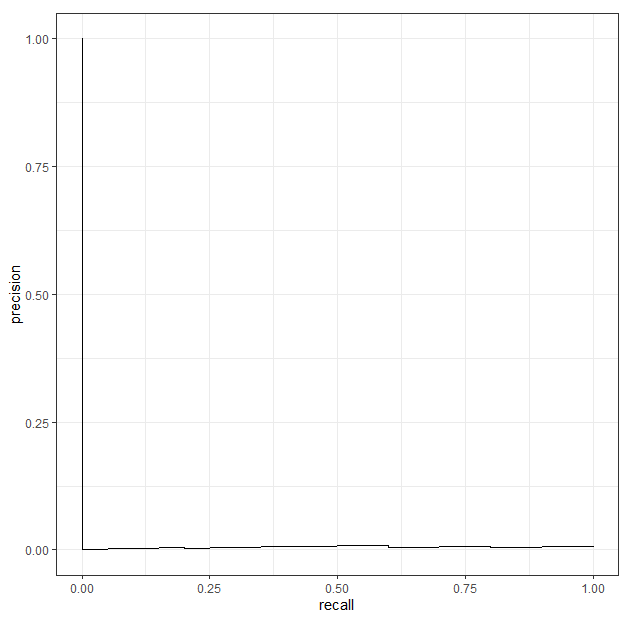


##### Table S65: Performance metrics

| **Thresholds** | **Performance metrics** |
| --- | --- |
| Optimal threshold to maximise MCC = 0.012 | True positives = 3  True negatives = 578  False negatives = 2  False positives = 338  MCC = 0.035  Sensitivity (recall) = 0.600  Specificity = 0.631  Youden index = 0.231  F statistic = 0.017  PPV (precision) = 0.009  NPV = 0.997 |
| Threshold-free metrics | Discrimination:  AUC ROC (training set) = 0.616  AUC ROC (test set) = 0.478 (0.209 to 0.747)  AUC PR = 0.005  Calibration:  Slope = -0.204 (-1.636 to 1.228)  Intercept = -0.824 (-1.705 to 0.056)  ICI = 0.008  E50 = 0.005  E90 = 0.015  Emax = 0.117 |
| MCC = Matthews Correlation Coefficient; PPV = positive predictive value; NPV = negative predictive value; AUC ROC = Area under the receiver operating characteristic curve; AUC PR = Area under the precision-recall (PPV-sensitivity) curve; ICI = Integrated calibration index; E50 = median of the absolute difference between observed and predicted probabilities; E90 = 90th percentile of the absolute difference between observed and predicted probabilities; Emax = maximal absolute difference between observed and predicted probabilities of the outcome | |

##### Figure S57: Calibration curve


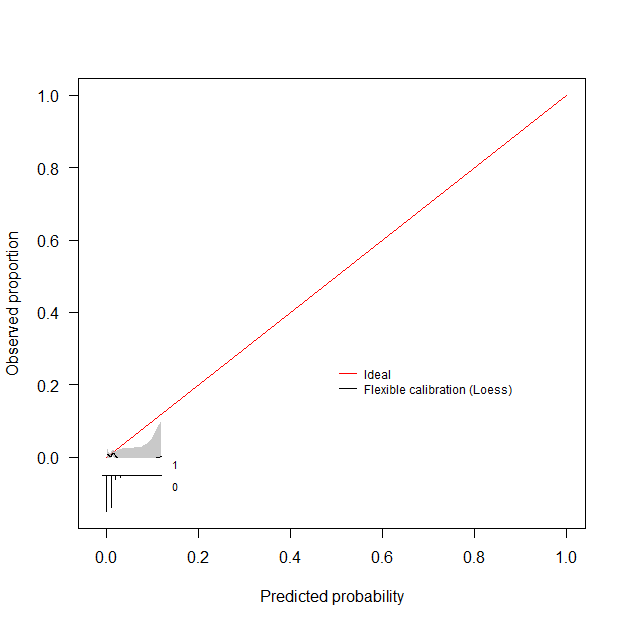


##### Table S66: Coefficients

| (Intercept) | -4.550 |
| --- | --- |
| Age | -0.123 |
| BMI | -0 |
| Varicose veins | -0.012 |
| Preoperative haemoglobin | -0.0856 |
| Sex | -0.0675 |
| Prior VTE | 0.288 |
| Factor V Leiden | 0.092 |
| Cancer (any) | 0 |
| Congestive heart failure | 0.159 |
| COPD | 0.215 |
| Rheumatoid arthritis | 0.053 |
| Haematological disorder | -0.026 |
| Warfarin therapy | -0.063 |
| Inflammatory bowel disease | -0.036 |
| Past AMI | -0.234 |
| Polio | 0 |
| Mechanical prophylaxis | -0.278 |
| Pharmacological thromboprophylaxis | 0 |
| Cemented prosthesis | -0.221 |

#### VTE full model evaluation - best administrative database – initial consultation, random forest (Figures S58-S60, Table S67, Figure S61, Table S68)

##### Figure S58: Training set ROC curve


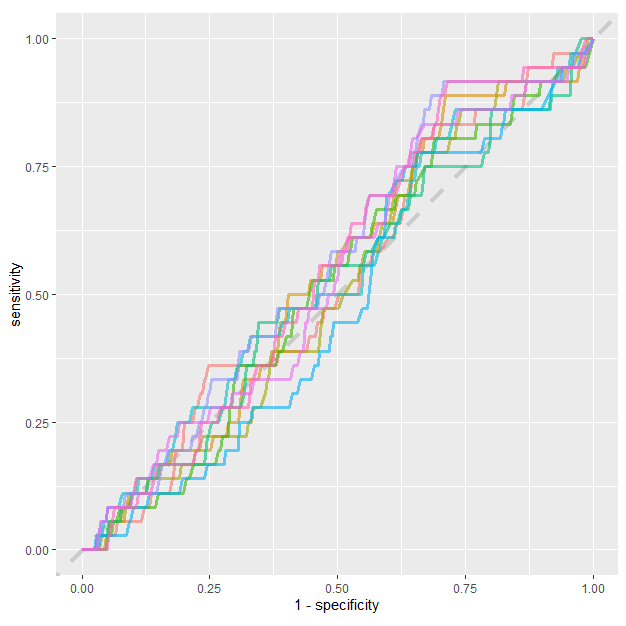


##### Figure S59: Test ROC curve


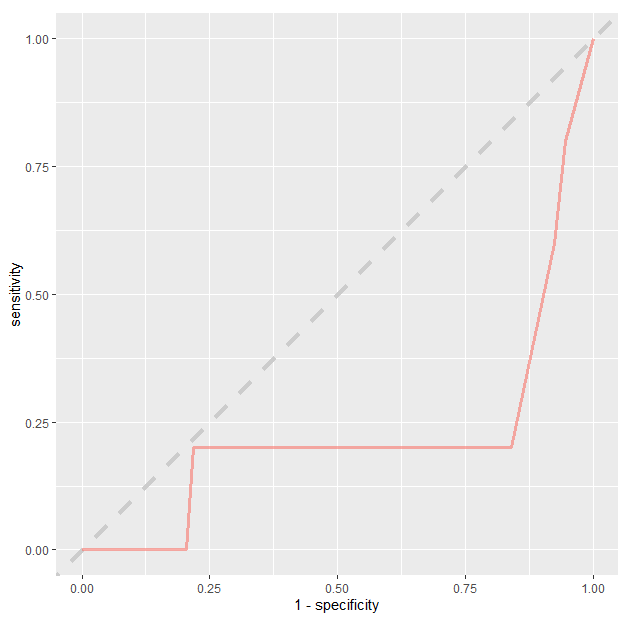


##### Figure S60: Precision-recall (PPV-sensitivity) curve


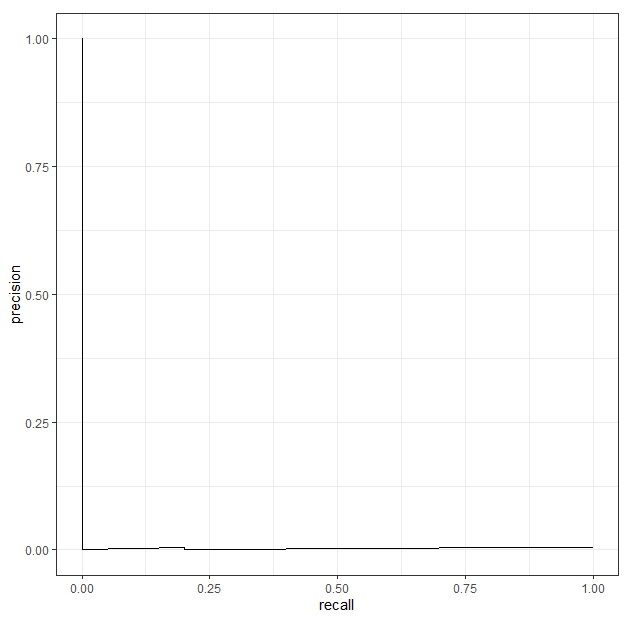


##### Table S67: Performance metrics

| **Thresholds** | **Performance metrics** |
| --- | --- |
| Optimal threshold to maximise MCC = 0.007 | True positives = 1  True negatives = 146  False negatives = 4  False positives = 770  MCC = 0.127  Sensitivity (recall) = 0.159  Specificity = 0.200  Youden index = -0.641  F statistic = 0.003  PPV (precision) = 0.001  NPV = 0.973 |
| Threshold-free metrics | Discrimination:  AUC ROC (training set) = 0.549  AUC ROC (test set) = 0.233 (0.003 to 0.500)  AUC PR = 0.003  Calibration:  Slope = -2.485 (-4.738 to -0.231)  Intercept = -0.887 (-1.767 to -0.007)  ICI = 0.012  E50 = 0.013  E90 = 0.016  Emax = 0.095 |
| MCC = Matthews Correlation Coefficient; PPV = positive predictive value; NPV = negative predictive value; AUC ROC = Area under the receiver operating characteristic curve; AUC PR = Area under the precision-recall (PPV-sensitivity) curve; ICI = Integrated calibration index; E50 = median of the absolute difference between observed and predicted probabilities; E90 = 90th percentile of the absolute difference between observed and predicted probabilities; Emax = maximal absolute difference between observed and predicted probabilities of the outcome | |

##### Figure S61: Calibration curve


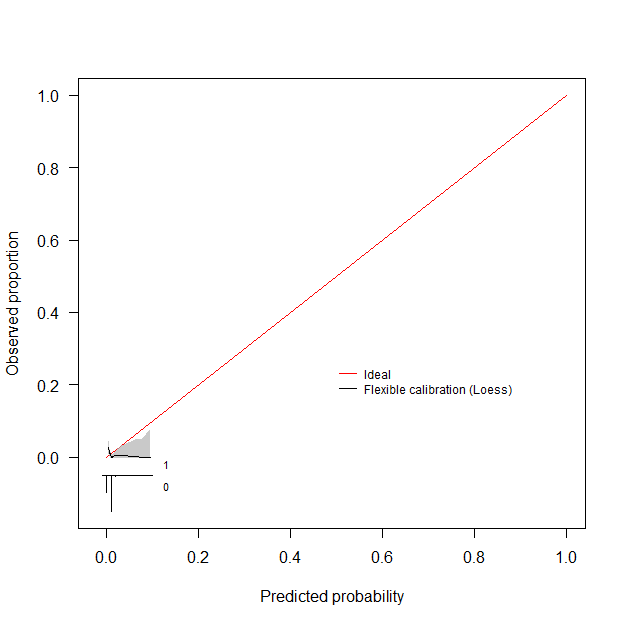


##### Table S68: Variable importance factors

| Age | 1.112 |
| --- | --- |
| Sex | 0.192 |

#### Joint-specific full model evaluation - best overall (broad definition, combined dataset, discharge, random forest) – Figures S62-S64, Table S69, Figure S65, Table S70)

##### Figure S62: Training set ROC curve


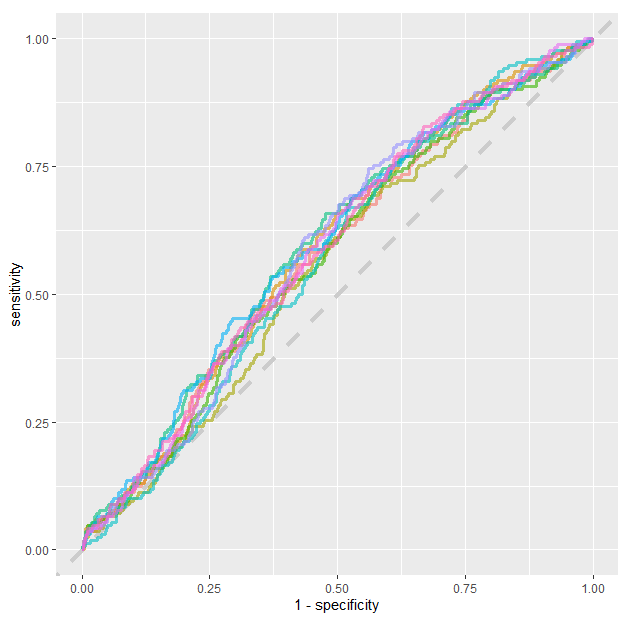


##### Figure S63: Test ROC curve


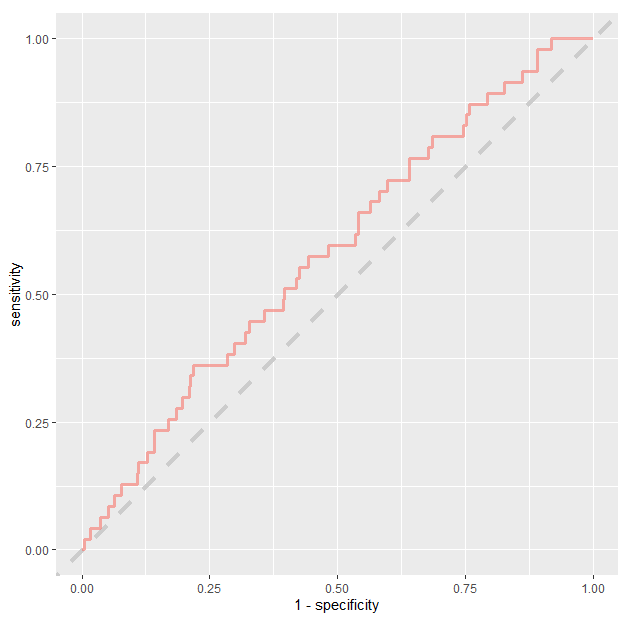


##### Figure S64: Precision-recall (PPV-sensitivity) curve


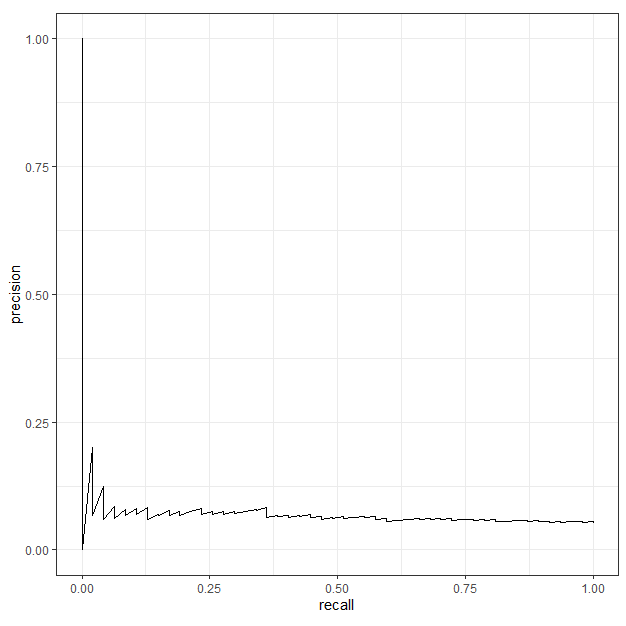


##### Table S69: Performance metrics

| **Thresholds** | **Performance metrics** |
| --- | --- |
| Optimal threshold to maximise MCC = 0.091 | True positives = 17  True negatives = 684  False negatives = 30  False positives = 190  MCC = 0.076  Sensitivity (recall) = 0.362  Specificity = 0.783  Youden index = 0.144  F statistic = 0.134  PPV (precision) = 0.082  NPV = 0.958 |
| Threshold-free metrics | Discrimination:  AUC ROC (training set) = 0.585  AUC ROC (test set) = 0.582 (0.500 to 0.663)  AUC PR = 0.066  Calibration:  Slope = 0.376 (0.008 to 0.743)  Intercept = -0.296 (-0.595 to 0.003)  ICI = 0.026  E50 = 0.014  E90 = 0.062  Emax = 0.269 |
| MCC = Matthews Correlation Coefficient; PPV = positive predictive value; NPV = negative predictive value; AUC ROC = Area under the receiver operating characteristic curve; AUC PR = Area under the precision-recall (PPV-sensitivity) curve; ICI = Integrated calibration index; E50 = median of the absolute difference between observed and predicted probabilities; E90 = 90th percentile of the absolute difference between observed and predicted probabilities; Emax = maximal absolute difference between observed and predicted probabilities of the outcome | |

##### Figure S65: Calibration curve


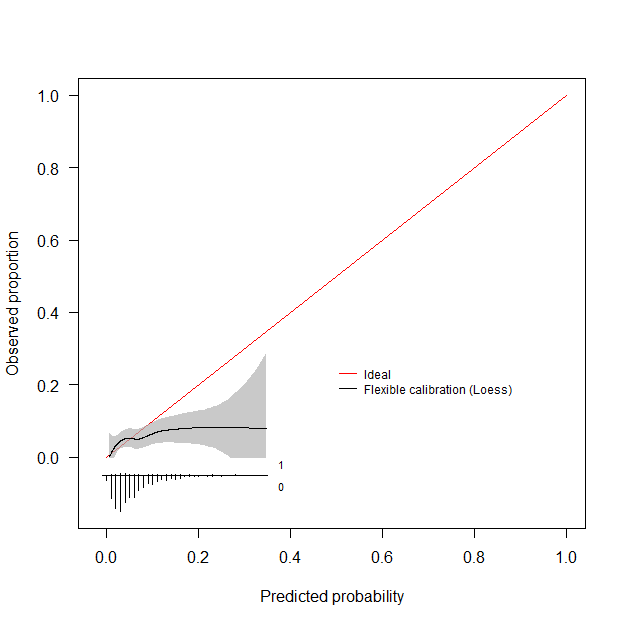


##### Table S70: Variable importance factors

| BMI | 48.563 |
| --- | --- |
| Age | 27.958 |
| Pre-discharge complication | 3.864 |
| Sex | 3.814 |
| Diabetes | 3.737 |
| Pensioner concession card | 3.470 |
| Depression | 3.264 |
| COPD | 3.134 |
| Other neurological disorder | 3.017 |
| Congestive heart failure | 2.682 |
| Chronic kidney disease | 2.658 |
| Liver disease | 1.644 |
| Lymphoma | 0.972 |
| Simultaneous bilateral TKA | 0.849 |
| Pulmonary hypertension | 0.541 |
| Coagulopathy | 0.052 |

#### Joint-specific full model evaluation - best administrative database – initial consultation (logistic regression) – Figure S66-S68, Table S71, Figure S69, Table S72

##### Figure S66: Training set ROC curve


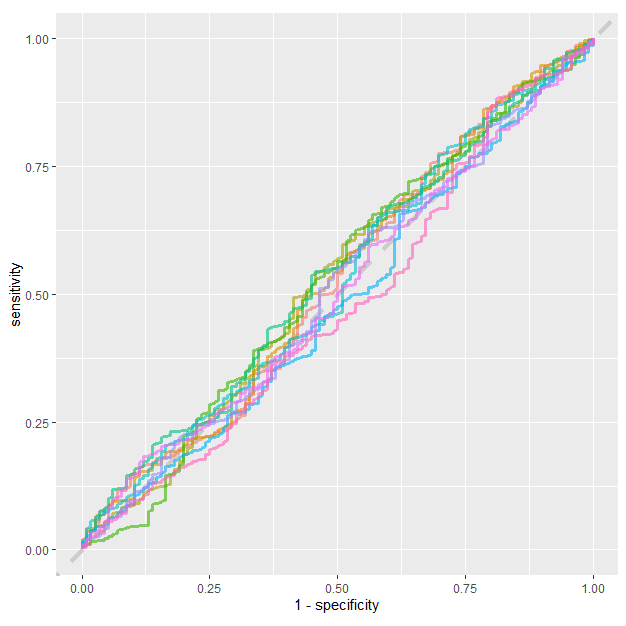


##### Figure S67: Test ROC curve


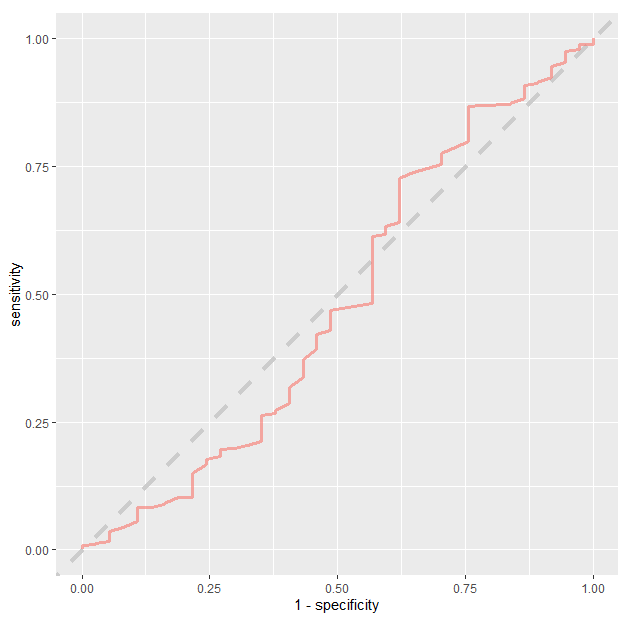


##### Figure S68: Precision-recall (PPV-sensitivity) curve


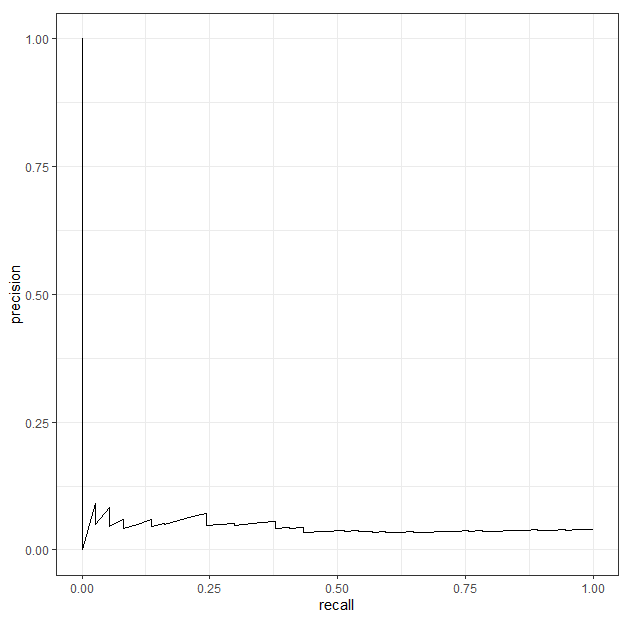


##### Table S71: Performance metrics

| **Thresholds** | **Performance metrics** |
| --- | --- |
| Optimal threshold to maximise MCC = 0.033 | True positives = 29  True negatives = 90  False negatives = 8  False positives = 794  MCC = 0.073  Sensitivity (recall) = 0.898  Specificity = 0.216  Youden index = -0.114  F statistic = 0.067  PPV (precision) = 0.035  NPV = 0.918 |
| Threshold-free metrics | Discrimination:  AUC ROC (training set) = 0.533  AUC ROC (test set) = 0.482 (0.408 to 0.629)  AUC PR = 0.044  Calibration:  Slope = -0.326 (-1.93 to 1.28)  Intercept = -0.057 (-0.386 to 0.273)  ICI = 0.015  E50 = 0.015  E90 = 0.024  Emax = 0.055 |
| MCC = Matthews Correlation Coefficient; PPV = positive predictive value; NPV = negative predictive value; AUC ROC = Area under the receiver operating characteristic curve; AUC PR = Area under the precision-recall (PPV-sensitivity) curve; ICI = Integrated calibration index; E50 = median of the absolute difference between observed and predicted probabilities; E90 = 90th percentile of the absolute difference between observed and predicted probabilities; Emax = maximal absolute difference between observed and predicted probabilities of the outcome | |

##### Figure S69: Calibration curve


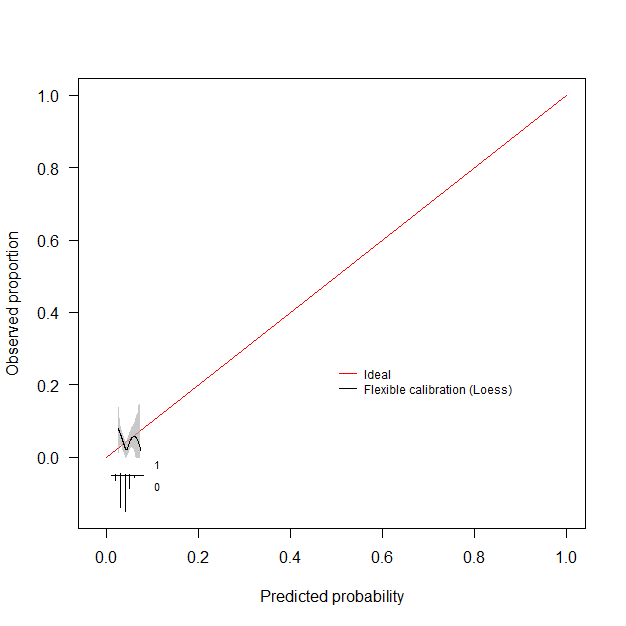


##### Table S72: Coefficients

| (Intercept) | 0.043 |
| --- | --- |
| Age | 0.871 |
| Sex | 1.120 |
| Pensioner concession card | 1.150 |

#### Combined outcome full model evaluation - best overall (combined dataset, discharge, random forest (Top k (227/10 = 22))) – Figures S70-S72, Table S73, Figure S73, Table S74

##### Figure S70: Training set ROC curve


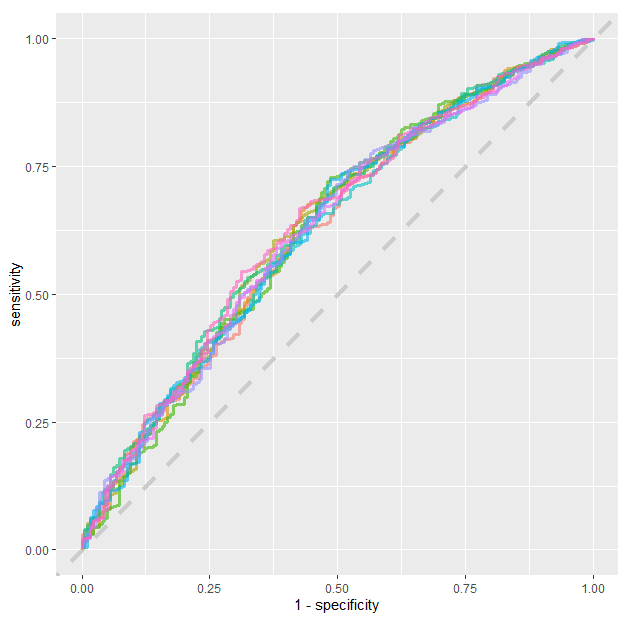


##### Figure S71: Test ROC curve


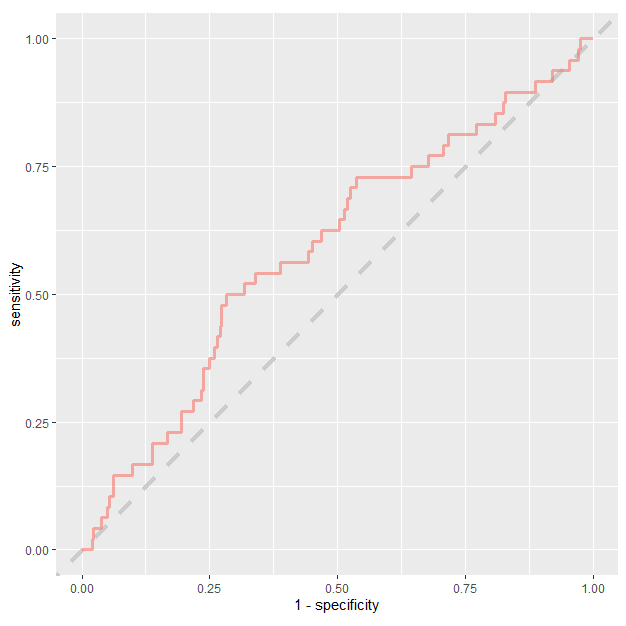


##### Figure S72: Precision-recall (PPV-sensitivity) curve


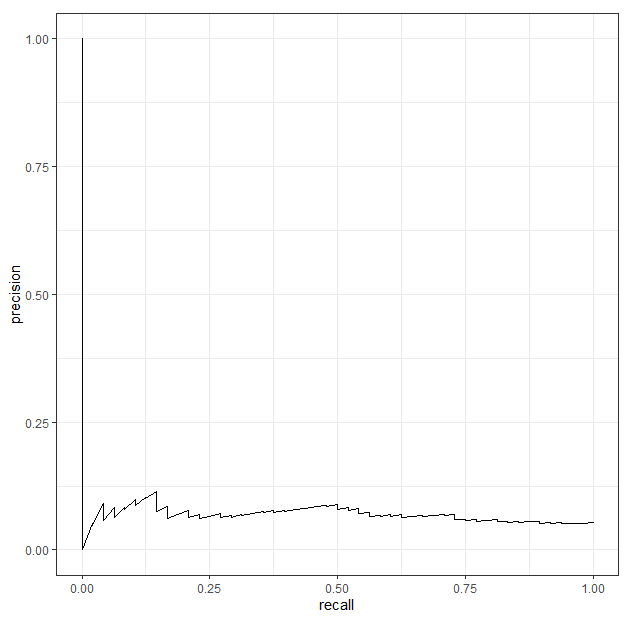


##### Table S73: Performance metrics

| **Thresholds** | **Performance metrics** |
| --- | --- |
| Optimal threshold to maximise MCC = 0.083 | True positives = 24  True negatives = 627  False negatives = 24  False positives = 246  MCC = 0.107  Sensitivity (recall) = 0.500  Specificity = 0.718  Youden index = 0.218  F statistic = 0.151  PPV (precision) = 0.089  NPV = 0.963 |
| Threshold-free metrics | Discrimination:  AUC ROC (training set) = 0.632  AUC ROC (test set) = 0.589 (0.504 to 0.674)  AUC PR = 0.068  Calibration:  Slope = -0.311 (-0.021 to 0.644)  Intercept = -0.384 (-0.681 to -0.087)  ICI = 0.032  E50 = 0.015  E90 = 0.083  Emax = 0.434 |
| MCC = Matthews Correlation Coefficient; PPV = positive predictive value; NPV = negative predictive value; AUC ROC = Area under the receiver operating characteristic curve; AUC PR = Area under the precision-recall (PPV-sensitivity) curve; ICI = Integrated calibration index; E50 = median of the absolute difference between observed and predicted probabilities; E90 = 90th percentile of the absolute difference between observed and predicted probabilities; Emax = maximal absolute difference between observed and predicted probabilities of the outcome | |

##### Figure S73: Calibration curve


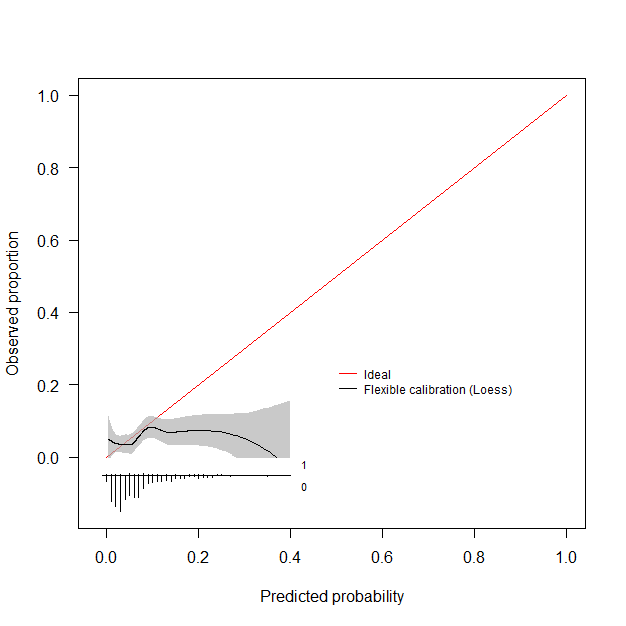


##### Table S74: Variable importance factors

| BMI | 31.427 |
| --- | --- |
| Preoperative haemoglobin | 27.117 |
| Duration of operation | 25.742 |
| Age | 22.772 |
| Surgeon ID | 17.151 |
| SEIFA | 13.744 |
| Transfusion during surgery | 6.747 |
| ASA class | 4.787 |
| Pre-discharge complication | 4.045 |
| Cancer (any) | 3.915 |
| Warfarin therapy | 3.744 |
| Hypertension | 3.661 |
| Diabetes | 3.478 |
| Pensioner concession card | 3.434 |
| Sex | 3.396 |
| Remoteness area | 3.367 |
| Summer surgery | 3.363 |
| Cemented prosthesis | 3.205 |
| Prior VTE | 3.089 |
| Depression | 3.055 |
| COPD | 2.941 |
| Factor V Leiden | 2.703 |

#### Combined outcome full model evaluation - best administrative database – initial consultation (logistic regression) (Figures S74-S76, Table S76, Figure S77, Table S77)

##### Figure S74: Training set ROC curve


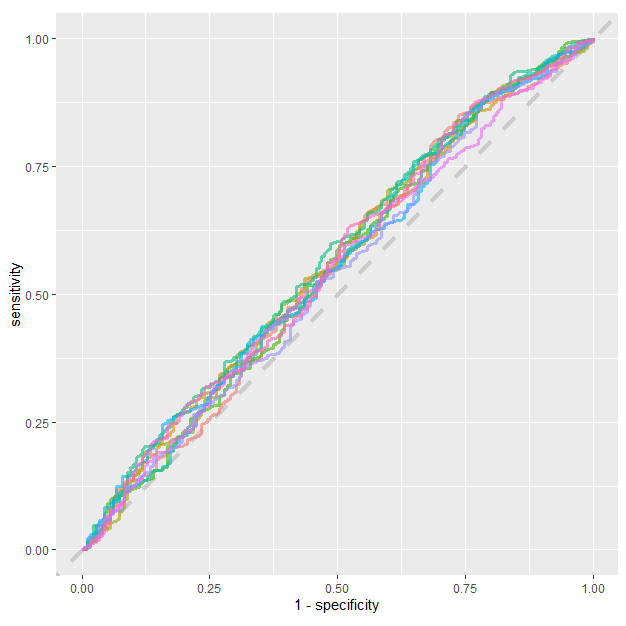


##### Figure S75: Test ROC curve


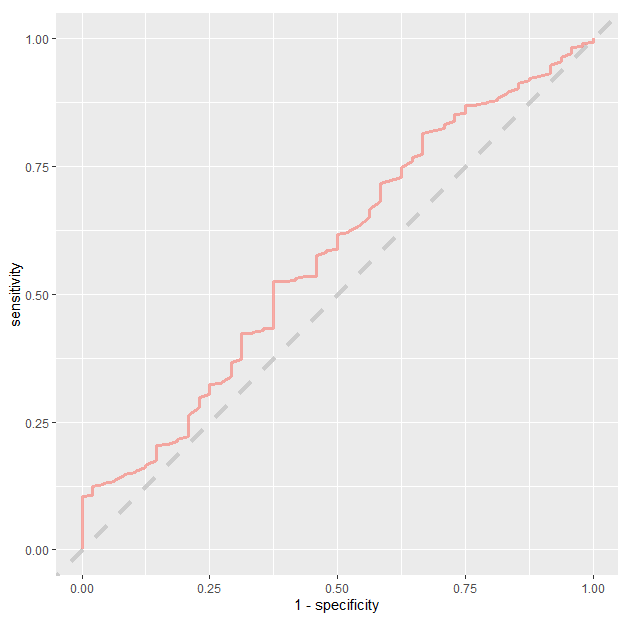


##### Figure S76: Precision-recall (PPV-sensitivity) curve


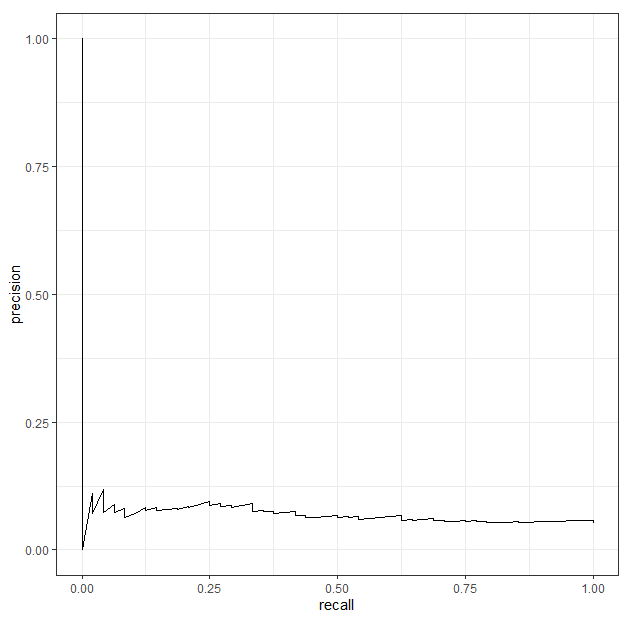


##### Table S76: Performance metrics

| **Thresholds** | **Performance metrics** |
| --- | --- |
| Optimal threshold to maximise MCC = 0.079 | True positives = 16  True negatives = 711  False negatives = 32  False positives = 162  MCC = 0.083  Sensitivity (recall) = 0.333  Specificity = 0.814  Youden index = 0.148  F statistic = 0.142  PPV (precision) = 0.090  NPV = 0.957 |
| Threshold-free metrics | Discrimination:  AUC ROC (training set) = 0.566  AUC ROC (test set) = 0.577 (0.493 to 0.662)  AUC PR = 0.068  Calibration:  Slope = 1.150 (0.095 to 2.206)  Intercept = -0.239 (-0.530 to 0.053)  ICI = 0.014  E50 = 0.017  E90 = 0.024  Emax = 0.075 |
| MCC = Matthews Correlation Coefficient; PPV = positive predictive value; NPV = negative predictive value; AUC ROC = Area under the receiver operating characteristic curve; AUC PR = Area under the precision-recall (PPV-sensitivity) curve; ICI = Integrated calibration index; E50 = median of the absolute difference between observed and predicted probabilities; E90 = 90th percentile of the absolute difference between observed and predicted probabilities; Emax = maximal absolute difference between observed and predicted probabilities of the outcome | |

##### Figure S77: Calibration curve


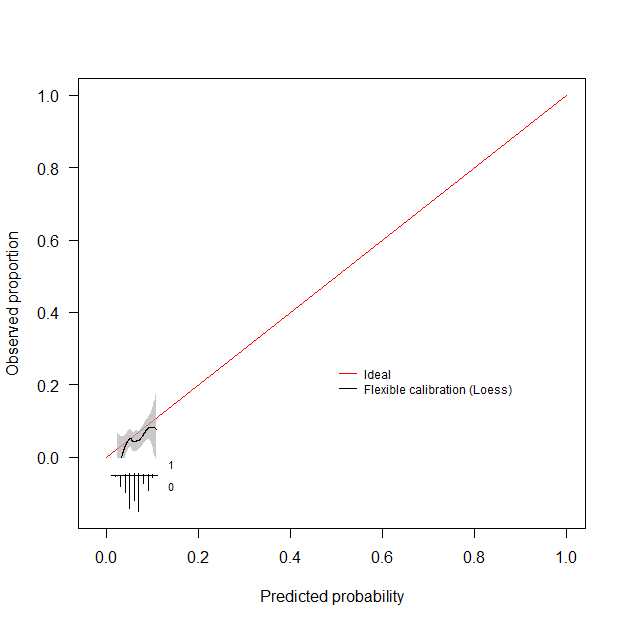


##### Table S77: Coefficients

| (Intercept) | 0.067 |
| --- | --- |
| Age | 0.941 |
| Sex | 1.130 |
| Remoteness area (major cities of Australia) | 1.150 |
| Remoteness area (outer regional or remote Australia) | 0.920 |
| Pensioner concession card | 1.230 |

#### Combined outcome all predictors full model evaluation - best overall (combined dataset, discharge, random forest (Top k (227/10 = 22))) – Figures S78-S79, Table S78, Figure S80, Table S79

##### Figure S78: Training set ROC curve


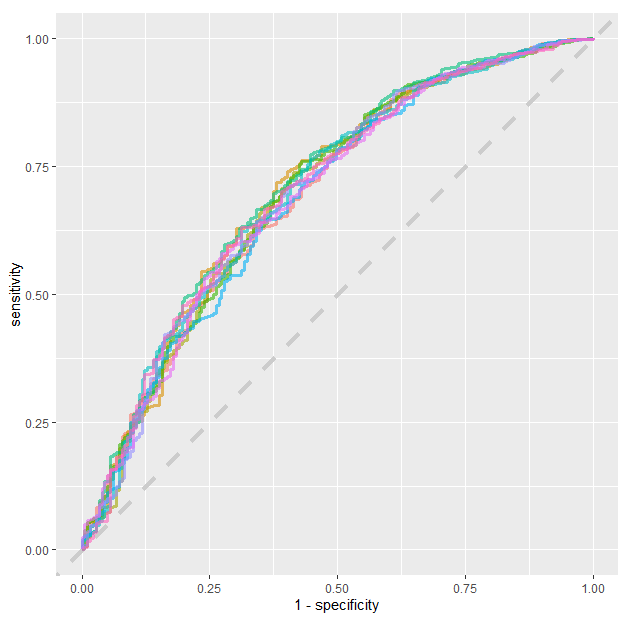


Test set ROC curve is located in main text

##### Figure S79: Precision-recall (PPV-sensitivity) curve


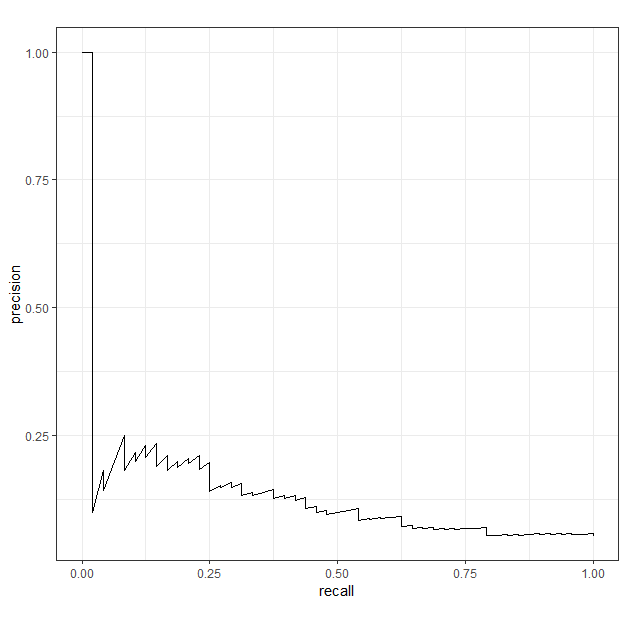


##### Table S78: Performance metrics

| **Thresholds** | **Performance metrics** |
| --- | --- |
| Optimal threshold to maximise MCC = 0.223 | True positives = 11  True negatives = 832  False negatives = 37  False positives = 41  MCC = 0.175  Sensitivity (recall) = 0.229  Specificity = 0.953  Youden index = 0.182  F statistic = 0.220  PPV (precision) = 0.212  NPV = 0.957 |
| Threshold-free metrics | Discrimination:  AUC ROC (training set) = 0.700  AUC ROC (test set) = 0.658 (0.570 to 0.746)  AUC PR = 0.133  Calibration:  Slope = 0.632 (0.332 to 0.932)  Intercept = -0.363 (-0.663 to -0.064)  ICI = 0.030  E50 = 0.018  E90 = 0.073  Emax = 0.117 |
| MCC = Matthews Correlation Coefficient; PPV = positive predictive value; NPV = negative predictive value; AUC ROC = Area under the receiver operating characteristic curve; AUC PR = Area under the precision-recall (PPV-sensitivity) curve; ICI = Integrated calibration index; E50 = median of the absolute difference between observed and predicted probabilities; E90 = 90th percentile of the absolute difference between observed and predicted probabilities; Emax = maximal absolute difference between observed and predicted probabilities of the outcome | |

##### Figure S80: Calibration curve


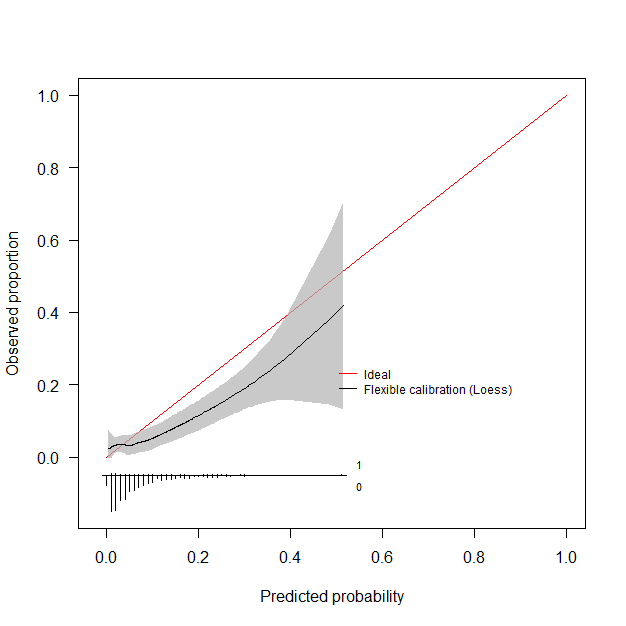


##### Table S79: Variable importance factors

| Length of stay | 27.870 |
| --- | --- |
| VR-12 mental score | 23.818 |
| BMI | 23.014 |
| Preoperative haemoglobin | 20.208 |
| VR-12 physical score | 19.473 |
| Duration of operation | 17.800 |
| Age | 16.245 |
| Surgeon ID | 12.732 |
| Historical knee procedures | 10.557 |
| SEIFA | 10.055 |
| Transfusion during surgery | 5.506 |
| VR-12 pain score | 5.695 |
| ASA class | 3.468 |
| Pre-discharge complication | 3.288 |
| Charlson Comorbidity Index | 4.101 |
| Factor V Leiden | 2.600 |
| Warfarin therapy | 2.691 |
| Cancer (any) | 2.655 |
| Prior VTE | 2.634 |
| Pensioner concession card | 2.274 |
| Sex | 2.551 |
| Hypertension | 2.528 |

#### Combined outcome all predictors full model evaluation - best administrative database – initial consultation (regression) – Figures S81-S83, Tables S80-S81

##### Figure S81: Training set ROC curve


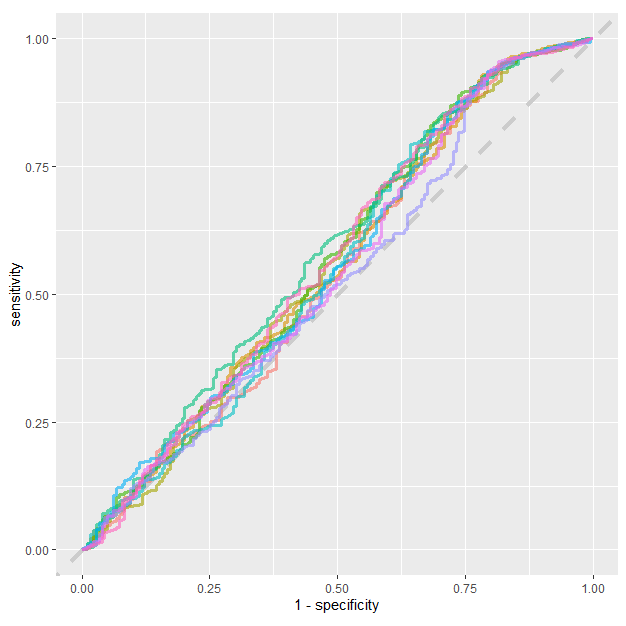


##### Figure S82: Test ROC curve


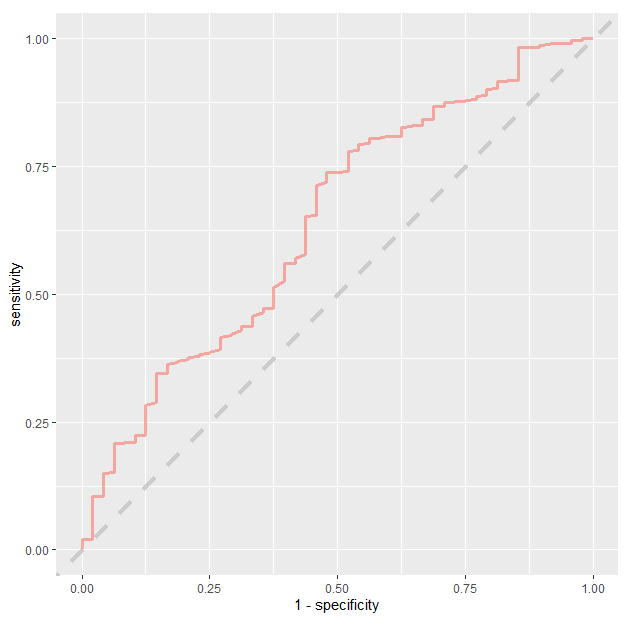


##### Figure S83: Precision-recall (PPV-sensitivity) curve


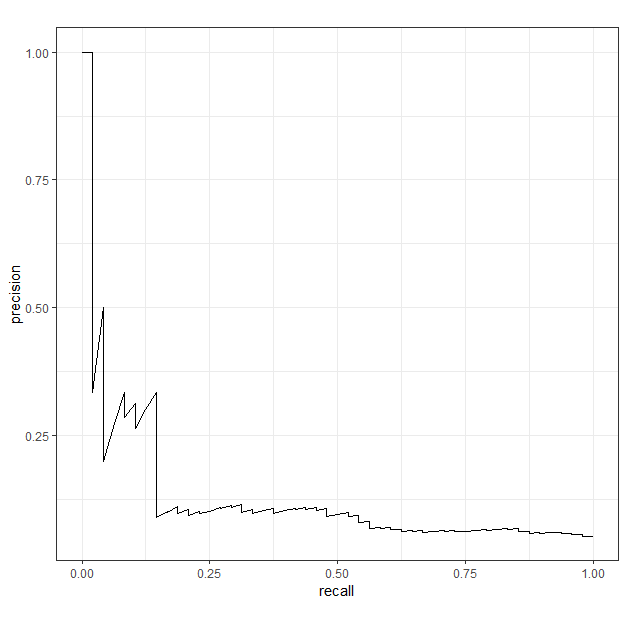


##### Table S80: Performance metrics

| **Thresholds** | **Performance metrics** |
| --- | --- |
| Optimal threshold to maximise MCC = 0.233 | True positives = 7  True negatives = 859  False negatives = 41  False positives = 14  MCC = 0.193  Sensitivity (recall) = 0.146  Specificity = 0.984  Youden index = 0.130  F statistic = 0.203  PPV (precision) = 0.333  NPV = 0.954 |
| Threshold-free metrics | Discrimination:  AUC ROC (training set) = 0.565  AUC ROC (test set) = 0.640 (0.557 to 0.723)  AUC PR = 0.129  Calibration:  Slope = 0.970 (0.497 to 1.443)  Intercept = -0.221 (-0.515 to 0.073)  ICI = 0.012  E50 = 0.009  E90 = 0.020  Emax = 0.497 |
| MCC = Matthews Correlation Coefficient; PPV = positive predictive value; NPV = negative predictive value; AUC ROC = Area under the receiver operating characteristic curve; AUC PR = Area under the precision-recall (PPV-sensitivity) curve; ICI = Integrated calibration index; E50 = median of the absolute difference between observed and predicted probabilities; E90 = 90th percentile of the absolute difference between observed and predicted probabilities; Emax = maximal absolute difference between observed and predicted probabilities of the outcome | |

Calibration curve is located in main text

##### Table S81: Coefficients

| (Intercept) | 0.063 |
| --- | --- |
| Age | 0.974 |
| Sex | 1.090 |
| Remoteness area (major cities of Australia) | 1.120 |
| Remoteness area (outer regional or remote Australia) | 0.894 |
| Pensioner concession card | 1.150 |
| Admissions in the past 12 months (one) | 0.971 |
| Admissions in the past 12 months (two | 0.895 |
| Admissions in the past 12 months (three or more) | 0.981 |
| Emergency presentations in the past 12 months (one) | 1.180 |
| Emergency presentations in the past 12 months (two or more) | 1.050 |
| Historical knee procedures (one) | 0.896 |
| Historical knee procedures (two) | 0.961 |
| Historical knee procedures (three or more) | 1.340 |

# References

1. Chicco D, Jurman G. The advantages of the Matthews correlation coefficient (MCC) over F1 score and accuracy in binary classification evaluation. BMC genomics. 2020;21(1):1-13.

2. Ramaswamy A, Marchese M, Cole AP, Harmouch S, Friedlander D, Weissman JS, et al. Comparison of Hospital Readmission After Total Hip and Total Knee Arthroplasty vs Spinal Surgery After Implementation of the Hospital Readmissions Reduction Program. JAMA Network Open. 2019;2(5):e194634.

3. Zmistowski B, Restrepo C, Hess J, Adibi D, Cangoz S, Parvizi J. Unplanned readmission after total joint arthroplasty: rates, reasons, and risk factors. Journal of Bone & Joint Surgery - American Volume. 2013;95(20):1869-76.

4. Li T, Zhang H, Chan PK, Fung WC, Fu H, Chiu KY. Risk factors associated with surgical site infections following joint replacement surgery: a narrative review. Arthroplasty. 2022;4(1):1-8.

5. Ramkumar PN, Chu C, Harris J, Athiviraham A, Harrington M, White D, et al. Causes and rates of unplanned readmissions after elective primary total joint arthroplasty: a systematic review and meta-analysis. Am J Orthop. 2015;44(9):397-405.

6. Curtis GL, Jawad M, Samuel LT, George J, Higuera-Rueda CA, Little BE, et al. Incidence, causes, and timing of 30-day readmission following total knee arthroplasty. The Journal of arthroplasty. 2019;34(11):2632-6.

7. Anthony CA, Peterson RA, Sewell DK, Polgreen LA, Simmering JE, Callaghan JJ, et al. The Seasonal Variability of Surgical Site Infections in Knee and Hip Arthroplasty. Journal of Arthroplasty. 2018;33(2):510-4.e1.

8. White RH, Henderson MC. Risk factors for venous thromboembolism after total hip and knee replacement surgery. Current opinion in pulmonary medicine. 2002;8(5):365-71.

9. Krauss ES, Segal A, Cronin M, Dengler N, Lesser ML, Ahn S, et al. Implementation and validation of the 2013 Caprini score for risk stratification of arthroplasty patients in the prevention of venous thrombosis. Clinical and Applied Thrombosis/Hemostasis. 2019;25:1076029619838066.

10. D'Apuzzo M, Westrich G, Hidaka C, Jung Pan T, Lyman S. All-Cause Versus Complication-Specific Readmission Following Total Knee Arthroplasty. Journal of Bone & Joint Surgery - American Volume. 2017;99(13):1093-103.

11. Alvarez AP, Demzik AL, Alvi HM, Hardt KD, Manning DW. Risk factors for postoperative urinary tract infections in patients undergoing total joint arthroplasty. Advances in orthopedics. 2016;2016.

12. Bohl DD, Sershon RA, Fillingham YA, Della Valle CJ. Incidence, risk factors, and sources of sepsis following total joint arthroplasty. The Journal of arthroplasty. 2016;31(12):2875-9. e2.

13. Bohl DD, Saltzman BM, Sershon RA, Darrith B, Okroj KT, Della Valle CJ. Incidence, risk factors, and clinical implications of pneumonia following total hip and knee arthroplasty. The Journal of arthroplasty. 2017;32(6):1991-5. e1.

14. Bovonratwet P, Bohl DD, Malpani R, Nam D, Della Valle CJ, Grauer JN. Incidence, Risk Factors, and Impact of Clostridium difficile Colitis Following Primary Total Hip and Knee Arthroplasty. Journal of Arthroplasty. 2018;33(1):205-10.e1.

15. Nguyen AQ, Foy MP, Sood A, Gonzalez MH. Preoperative risk factors for postoperative urinary tract infection after primary total hip and knee arthroplasties. The Journal of arthroplasty. 2021;36(2):734-8.

16. Adenikinju AS, Feng JE, Namba CA, Luthringer TA, Lajam CM. Gastrointestinal complications warranting invasive interventions following total joint arthroplasty. The Journal of arthroplasty. 2019;34(11):2780-4.

17. Massaglia J, Yayac M, Star A, Deirmengian G, Courtney PM, Saxena A. Gastrointestinal complications following total joint arthroplasty are Rare but have severe Consequences. The Journal of arthroplasty. 2021;36(8):2974-9.

18. Elsiwy Y, Jovanovic I, Doma K, Hazratwala K, Letson H. Risk factors associated with cardiac complication after total joint arthroplasty of the hip and knee: a systematic review. Journal of orthopaedic surgery and research. 2019;14(1):1-12.

19. Statistics ABO. Socio-economic indexes for areas (SEIFA). Canberra: Australian Bureau of Statistics. 2011.
